# Comparing YAP^WW1^ and TAZ^WW^: Similar Binding Sites but Different Stability and Conformational Dynamics

**DOI:** 10.3390/biom16091355

**Published:** 2026-09-17

**Authors:** Clara Merlen, Yannick Mesrouze, Suzanne Chau, Benjamin A. Diehl, Alexandra Hinniger, Catherine Zimmermann, Marco Meyerhofer, Patrizia Fontana, Svenya Groebke, Jürgen Hinrichs, Wassim Abdul Rahman, Sascha Gutmann, César Fernández, Dušan Petrović, Patrick Chène

**Affiliations:** 1 Oncology Disease Area, Novartis Biomedical Research, CH-4056 Basel, Switzerland; 2 Discovery Sciences, Novartis Biomedical Research, CH-4056 Basel, Switzerland; 3 Global Discovery Chemistry, Novartis Biomedical Research, CH-4056 Basel, Switzerland

**Keywords:** YAP, TAZ, WW domain, conformational flexibility, molecular recognition, PPxY motif

## Abstract

The two paralogs YAP and TAZ act through TEAD transcription factors and bind PPxY motif proteins in the Hippo pathway via WW domains. YAP has up to two WW domains, whereas TAZ has one. Because YAP^WW1^ and TAZ^WW^ are the most similar in sequence, they can be considered as corresponding modules in these two proteins. This study shows that, despite their similarity, they differ strongly in conformational stability and in how they bind flexible ligands. Nano-differential scanning fluorimetry, circular dichroism, and NMR indicate that both isolated domains populate partially folded or exchanging states in solution, but TAZ^WW^ is more thermally stable and has a larger folded population. Peptide binding stabilizes both domains, producing sharper NMR signals. Surface plasmon resonance measurements with PPxY peptides show micromolar affinities and generally modest differences between YAP^WW1^ and TAZ^WW^, although these differences increase for conformationally plastic ligands. A high resolution LATS2:YAP^WW1^ crystal structure and molecular dynamics simulations suggest that preorganized peptides bind more tightly and less selectively, while flexible peptides incur larger binding penalties that dynamic YAP^WW1^ compensates more effectively. These observations indicate that differences in WW-domain stability contribute to subtle YAP/TAZ binding preferences despite conserved binding surfaces and evolutionary relatedness between the paralogs.

## 1. Introduction

The Hippo pathway, which plays a crucial role in organ morphogenesis [1,2], has received significant attention due to its role in cancer [3,4]. Ongoing drug discovery efforts aim to develop molecules that target tumors with deregulated Hippo pathway activity by inhibiting the interaction between the TEAD transcription factors [5] and their regulators, the YAP/TAZ proteins [6,7,8] typically by binding to the TEAD surface, resulting in the inactivation of this transcription factor and triggering an anti-tumor response [9,10,11].

YAP/TAZ also interact with other proteins through their WW domains, which are distinct from their TEAD-binding domain [12]. WW domains are small protein modules, about 40 residues in length, that form a three-stranded antiparallel β-sheet structure [13,14,15,16]. These domains mediate protein–protein interactions, with those in YAP/TAZ specifically recognizing regions with the PPxY motif, where x represents any amino acid. Thus, whether bound to TEAD or not, YAP/TAZ can interact with other proteins through their WW domains. The human proteome contains numerous protein sequences harboring a PPxY motif [17,18], and several proteins with this motif have been identified as interacting with YAP through its WW domains [19,20,21,22,23].

Humans possess twenty-three isoforms of YAP [24,25]. These isoforms feature one or two WW domains, referred to as WW1 (YAP^WW1^) and WW2 (YAP^WW2^) based on their position from the N-terminus. A short flexible linker, approximately 25 residues long, separates these two WW domains. Extensive research has been conducted to elucidate the characteristics of this WW tandem in target recognition [19,20,22,23]. It has been established that the coupling between these domains is minimal, resulting in low binding synergism [21]. Additionally, negative cooperativity has been observed between the WW domains [22]. Both domains function independently with distinct specificities, indicating that the presence of a second WW domain may play a role in modulating target recognition [19]. Notably, YAP^WW1^ has a more significant role in protein recognition than YAP^WW2^ [19,23].

**Figure 1:**
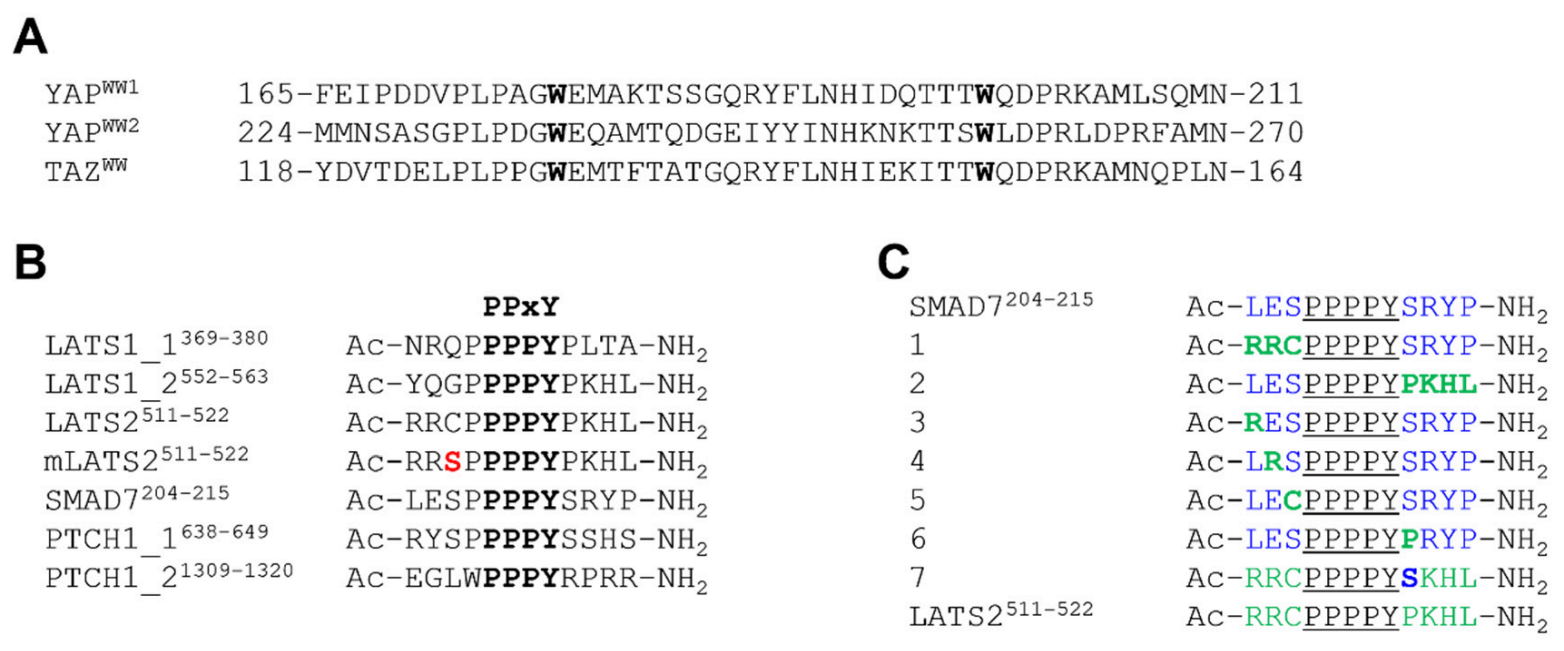
Amino acid sequences of the WW domains of YAP/TAZ and of the peptides used in this study. (**A**) Amino acid sequence of the WW domains present in YAP (UniProt P46937) and TAZ (UniProt Q9GZV5). The two conserved tryptophans are shown in bold. (**B**) Amino acid sequences of the peptides derived from human proteins. The PPxY motif is in bold. The serine mutation in LATS2 is indicated in red. The sources of the amino acid sequences are: LATS1 (UniProt O95835), LATS2 (UniProt Q9NRM7), SMAD7 (UniProt O15105), PTCH1 (UniProt Q13635). (**C**) Mutations of the SMAD7 peptide. The PPPPY region, conserved between SMAD7 and LATS2, is underlined and colored black. The other residues from SMAD7 and LATS2 are shown in blue and green, respectively. The mutations in **6** and **7** are highlighted in bold.

Unlike YAP, human TAZ contains only one WW domain (TAZ^WW^) [26], indicating that TAZ relies on a single WW domain for its interactions with other proteins. YAP^WW1^ and TAZ^WW^ share 62% sequence identity (sequences are shown in Figure 1A), whereas YAP^WW2^ and TAZ^WW^ share 53% identity. Therefore, YAP^WW1^ and TAZ^WW^ can be considered counterparts within these two paralogs. Despite their high sequence homology, these proteins function in different structural contexts. TAZ^WW^ operates as a single WW domain, whereas YAP^WW1^ forms part of a tandem WW domain in most isoforms [25].

Given that the properties of YAP^WW1^ and TAZ^WW^ have not been extensively compared (but see [27]), this study aims to elucidate the similarities and differences between the isolated YAP^WW1^ and TAZ^WW^ using biochemical and structural methods. We tested whether intrinsic differences in WW-domain stability and conformational dynamics modulate recognition of peptide ligands with varying degrees of conformational preorganization. Therefore, despite their sequence similarity and structurally similar PPxY-binding sites, these two isolated protein modules exhibit different biophysical properties and ligand-dependent recognition differences.

## 2. Material and Methods

### 2.1. Peptides

The synthetic N-acylated and C-amidated peptides were purchased from Biosyntan (Berlin, Germany). They were stored in 90:10 (*v*/*v*) dimethyl sulfoxide (DMSO):water. Their purity (>90%) and chemical integrity were determined by liquid chromatography and mass spectrometry (Supplementary Figure S11). The concentration of the peptides in the DMSO stock solutions was measured by HPLC (UV detection at 210 nm using a calibration curve generated from calcitonin standards).

### 2.2. Protein Expression and Purification: Production of Biotinylated YAP^WW1^ and TAZ^WW^ Domains

Genes encoding YAP^WW1^ (R161-Q209) or TAZ^WW^ (R114-P162), with either amino- or carboxy-terminal Avi-tags, were fused at the N-terminus with a polyhistidine tag followed by a Lipoyl-tag and a 3C-protease cleavage site and inserted in a bacterial expression vector. The protein of interest was co-expressed with BirA ligase in the BL21 (DE3) *Escherichia coli* strain at 18 °C overnight with 0.13 mM biotin and 1 mM isopropyl β-D-1-thiogalactopyranoside (IPTG). The cells were harvested by centrifugation at 6000× *g* for 20 min at 4 °C and the cell pellet was resuspended in lysis buffer (50 mM Tris-HCl pH 7.8, 300 mM NaCl, 30 mM imidazole, cOmplete protease inhibitor (Roche Diagnostics GmbH, Mannheim, Germany), 40 U/mL Turbonuclease from Sigma-Aldrich (St. Louis, MI, USA) and 1 mM biotin). Bacterial cells were lysed by three passages through a high-pressure homogenizer Avestin Emulsiflex C3 (Avestin Inc., Ottawa, ON, Canada) at 1000 bar and the lysate was centrifuged for 40 min at 40,000× *g*. The clarified lysate was loaded onto a HisTrap HP column (Cytiva, Marlborough, MA, USA). After washing the column, the protein was eluted by applying a 30–300 mM imidazole gradient in IMAC buffer (50 mM Tris-HCl pH 7.8, 300 mM NaCl). The eluted protein was mixed with His-MBP-tagged 3C protease and the mixture was dialyzed overnight at 4 °C against dialysis buffer (50 mM Tris pH 7.8, 300 mM NaCl). After cleavage, the mixture was loaded onto a HisTrap HP column and the flow-through containing the cleaved protein of interest was collected. The protein sample was concentrated with a 3-kDa cutoff Amicon Ultra concentrator (Millipore, Burlington, MA, USA) and loaded onto a HiLoad Superdex 75 16/600 column (Cytiva, Marlborough, MA, USA) pre-equilibrated with SEC buffer (50 mM HEPES pH 7.4, 100 mM KCl, 0.25 mM TCEP, 1 mM EDTA, 0.05% Tween 20). The eluted protein was collected, concentrated with a 3-kDa cutoff Amicon Ultra concentrator (Sigma-Aldrich, St. Louis, MI, USA) and snap-frozen. The final product was analyzed by SDS-PAGE and reversed-phase HPLC to determine protein concentration and purity. LC-MS was used to confirm protein identity.

### 2.3. Protein Expression and Purification: Production of Non-Biotinylated YAP^WW1^ and TAZ^WW^ Domains

Genes encoding either YAP^WW1^ (F165-Q209) or TAZ^WW^ (Y118-P162) with an AMG N-terminal extension were fused at the N-terminus with a polyhistidine tag followed by a Lipoyl tag and a TEV-protease cleavage site and inserted into a bacterial expression vector. Protein expression was performed in the NiCo21 (DE3) *E. coli* strain at 18 °C overnight with 0.2 mM IPTG. For ^15^N- and ^13^C-labeled proteins, the expression was performed in M9 minimal medium containing ^15^NH_4_Cl and ^13^C-glucose as the sole nitrogen and carbon sources, respectively [28] and the induction was achieved using the same conditions as for the unlabeled proteins. Cells were harvested by centrifugation at 6000× *g* for 20 min at 4 °C. The cell pellet, from a 2 L culture, was resuspended in lysis buffer (50 mM Tris-HCl pH 7.8, 300 mM NaCl, 30 mM imidazole, cOmplete protease inhibitor (Roche Diagnostics GmbH, Mannheim, Germany) and 40 U/mL Turbonuclease from Sigma-Aldrich (St. Louis, MI, USA)). Bacterial cells were lysed by three passages through a high-pressure homogenizer Avestin Emulsiflex C3 (Avestin Inc., Ottawa, ON, Canada) at 1000 bar and the lysate was centrifuged for 40 min at 40,000× *g*. The clarified lysate was loaded onto a HisTrap HP column (Cytiva, Marlborough, MA, USA). The column was washed and the protein was eluted by applying a 30–300 mM imidazole gradient in IMAC buffer (50 mM Tris-HCl pH 7.8, 300 mM NaCl). The eluted protein was mixed with TEV protease and the mixture was dialyzed overnight at 4 °C against dialysis buffer (50 mM Tris-HCl pH 7.8, 300 mM NaCl, 0.1 mM EDTA, 0.5 mM TCEP). After cleavage, the mixture was supplemented with 1 mM MgCl_2_ and loaded onto a HisTrap HP column (Cytiva, Marlborough, MA, USA) and the flow-through containing the cleaved protein of interest was collected. The protein sample was concentrated with a 3-kDa cutoff Amicon Ultra concentrator (Sigma-Aldrich, St. Louis, MI, USA) and loaded onto a HiLoad Superdex 75 16/600 column (Cytiva, Marlborough, MA, USA) pre-equilibrated with SEC buffer A (50 mM HEPES pH 7.4, 100 mM NaCl, 1 mM TCEP) for NMR experiments or SEC buffer B (20 mM Tris-HCl pH 8.0) for crystallization experiments. The eluted protein was collected, concentrated with 3-kDa cutoff Amicon Ultra concentrator (Sigma-Aldrich, St. Louis, MI, USA) and snap-frozen. The final product was analyzed by SDS-PAGE and reversed-phase HPLC to determine protein concentration and purity. LC-MS was used to confirm protein identity. To minimize potential effects of the tag, additional residues were inserted between the Avi-tag and the WW domains in some constructs. The N-terminal GAMG extension of the untagged proteins was introduced to mimic previously described YAP^WW1^ constructs [29,30] (see text below).

### 2.4. Nano-Differential Scanning Fluorimetry (NanoDSF)

NanoDSF was performed using a Prometheus NT.48 device (NanoTemper Technologies GmbH, Munich, Germany). Proteins were diluted to 10 µM in assay buffer (50 mM Tris pH 7.5, 50 mM KCl, 1 mM TCEP, 1% (*v*/*v*) DMSO) prior to being loaded into high-sensitivity capillaries (NanoTemper Technologies GmbH, Germany). The temperature was increased from 20 °C to 90 °C at 1 °C/min and the intrinsic fluorescence emission after UV excitation at 280 nm was collected at 330 nm. The melting temperatures (T_m_) were determined by analyzing the thermal denaturation curves with Panta Analysis software (NanoTemper Technologies GmbH, Germany).

### 2.5. Circular Dichroism (CD)

CD spectra were recorded on a J-815 spectropolarimeter (Jasco, Pfungstadt, Germany), using a 1 mm quartz cell, standard sensitivity, a 1 nm bandwidth, a scan rate of 10 nm min^−1^, a digital integration time of 1 s and a step resolution of 1 nm; the sample chamber was maintained under nitrogen. Proteins were dialyzed overnight at 4 °C in 20 mM monosodium/disodium phosphate pH 7.4, 100 mM KF, 0.25 mM TCEP, and subsequently diluted in the same buffer. Each spectrum was recorded as an average of eight scans. After baseline correction, the CD data were converted into mean residue ellipticity using the equation: [Ɵ]_MRW,λ_ = (MRW*Ɵ_λ_)/(10*d*c) (where MRW is the mean residue weight defined as MRW = M/(N − 1) (M is the molecular mass, N is the number of amino acids), Ɵ_λ_ is the observed ellipticity in degrees at wavelength λ, d is the path length of the cell in cm, and c is the protein concentration in g mL^−1^. The graphs shown in this study correspond to the average of 2 to 3 independent CD experiments.

### 2.6. Surface Plasmon Resonance (SPR)

All the experiments were carried out with a Biacore 8K optical biosensor and Series S sensor Chip SA (Cytiva, Marlborough, MA, USA). Chips were washed three times with 1 M NaCl/50 mM NaOH. Biotinylated N-Avi-tagged proteins were injected at a flow rate of 5 µL/min in SPR immobilization buffer (50 mM Tris pH 7.5, 50 mM KCl, 1 mM TCEP, 0.05% (*v*/*v*) Tween 20, 0.05% (*w*/*w*) BSA) for 165 s. The experiments were performed at 25 °C with a flow rate of 50 µL/min in SPR running buffer (SPR immobilization buffer containing 2% (*v*/*v*) DMSO). The tested analytes were diluted in SPR running buffer. After baseline equilibration with a series of buffer blanks, a DMSO correction series (from 1 to 3%) was performed. All data were referenced against a blank streptavidin reference surface and blank injections of running buffer to minimize the influence of baseline drift upon binding. Each cycle consisted of an injection phase of peptide (50 s) and a dissociation phase (100 s).

A three-step workflow was applied to obtain the dissociation constants measured at equilibrium (K_d_^eq^). Step 1: sensorgrams were globally fitted with a 1:1 interaction model using the Biacore 8K Evaluation software (Cytiva, Marlborough, MA, USA). Step 2: sensorgrams were visually inspected and low-quality experiments (e.g., large bulk effects, unstable signal at equilibrium, inappropriate peptide concentration range, etc.) were not further analyzed. Step 3: experiments with a standard error on fitted K_d_^eq^ higher than 20% of K_d_^eq^ or an experimental fitted maximum binding capacity (R_max_^exp^) < 60% of the theoretical R_max_ (R_max_^th^) were discarded, with R_max_^th^ = (MW^peptide^/MW^protein^)*R_protein_*n, where MW^peptide^ and MW^protein^ are the molecular weights of peptides and proteins, respectively, R_protein_ is the level of immobilization of proteins in response units (RU) and n is the stoichiometry; here n = 1.

### 2.7. Nuclear Magnetic Resonance (NMR)

For resonance assignments, uniformly ^13^C,^15^N-labeled YAP^WW1^ and TAZ^WW^ samples were prepared in 20 mM d_18_-HEPES, 150 mM NaCl, 1 mM d_16_-TCEP, 5% D_2_O, pH 7.0 and concentrated to 500 µM. All NMR spectra were measured in 3 mm NMR tubes with sample volumes of 170 µL, either at 5 °C or 23 °C, on Bruker Avance III HD 800 MHz or 600 MHz spectrometers (Bruker, Fällanden, Switzerland) equipped with four radio-frequency channels and a 5 mm ^1^H[^13^C,^15^N]-triple resonance cryogenic probe. Backbone assignments were obtained using 2D ^15^N-HSQC and 3D HNCA, CBCANH and CBCA(CO)NH experiments [31,32], complemented with a 3D ^15^N-edited [^1^H,^1^H]-NOESY (120 ms mixing time). The ^13^C- and ^15^N-HSQC spectra of the YAP^WW1^ and TAZ^WW^ complexes with the LATS1_2 and the LATS2 peptides were identical and thus allowed us to transfer the resonance assignments to the corresponding complexes with the help of 3D HNCA, CBCANH and CBCA(CO)NH. All the NMR data were processed with Topspin 3.6 software (Bruker, Fällanden, Switzerland) and analyzed with CCPNMR (v 3.0, https://doi.org/10.1002/prot.20449; https://doi.org/10.1007/s10858-016-0060-y).

For subsequent NMR binding experiments using ^13^C and ^15^N-HSQC spectra, samples containing 100 µM ^13^C,^15^N-labeled YAP^WW1^ or 100 µM ^13^C,^15^N-labeled TAZ^WW^ and 800 µM LATS2 peptides were prepared in the same buffer as used for resonance assignments.

### 2.8. X-Ray Diffraction Data Collection and Processing

Crystals of YAP^WW1^ (residues 165–209) in complex with mLATS2 were obtained at 20 °C in 96-well SWISSCI SD-2 crystallization plates with sitting drops. The final crystallization conditions were created by mixing 0.2 µL of the well solution containing 0.2 M ammonium acetate, 0.1 M Bis-Tris pH 6.5, and 25% (*w*/*v*) PEG3350 with 0.2 µL of the protein solution consisting of 3.6 mM YAP^WW1^ and 4 mM mLATS2 in 0.02 M Tris-HCl pH 8.0. Crystals appeared after 3–5 days.

For data collection, the crystals were transferred to the respective reservoir solution supplemented with 10% (*w*/*v*) PEG3350 for cryoprotection, mounted onto cryoloops (Hampton Research), and flash-cooled in liquid nitrogen. The crystals were measured at the ESRF (Grenoble, France) on beamline ID23-1.

Diffraction data were processed with autoPROC (v1.1.7, Global Phasing Ltd., Cambridge, UK) [33] and Aimless (v1.12.12) [34]. The structure of YAP^WW1^ in complex with mLATS2 was solved by molecular replacement using Phaser 2.7.17 [35] and an unpublished in-house structure of YAP^165–209^ as the search model. Models were built with Coot 0.9.8.7 [36] and refined with phenix.refine (1.21.2-5419) [37]. Geometric correctness and Ramachandran plots were assessed using the phenix.table_one tool implemented in phenix.refine. The final structure was submitted to the RCSB Protein Data Bank. PDB ID: 32dh. Details on data collection and refinement statistics are given in Supplementary Figure S1. All structure figures were generated using PyMOL (PyMOL Molecular Graphics System version 2.1.0; Schrödinger LLC, Cambridge, MA, USA).

### 2.9. Molecular Dynamics Simulations

Starting structure coordinates for the YAP^WW1^-bound SMAD7 and LATS2 peptides were obtained from PDB 2ltw and 32dh (present work), respectively. In the absence of experimental structures of TAZ^WW^ bound to the two peptides, we modeled TAZ^WW^-SMAD7 and TAZ^WW^-LATS2 complexes using the deep learning-based co-folding method Boltz-2 [38]. Five samples were produced for each structure, and both models were predicted with high confidence metrics (LATS2 complex confidence_score = 0.84, ptm = 0.95, protein_iptm = 0.94, complex_plddt = 0.82, complex_iplddt = 0.87, complex_pde = 0.27, complex_ipde = 0.31; SMAD7 complex confidence_score = 0.87, ptm = 0.95, protein_iptm = 0.94, complex_plddt = 0.86, complex_iplddt = 0.91, complex_pde = 0.27, complex_ipde = 0.32). The complexes were in excellent agreement with the YAP^WW1^-based structures (Supplementary Figure S2). Consequently, the resulting models were considered suitable starting points for comparative MD simulations of the YAP^WW1^ and TAZ^WW^ complexes, although the interpretation of TAZ^WW^-specific dynamical features should be viewed in the context of their model-derived starting structures. In addition to the protein-peptide bound simulations, we also investigated the peptide-only systems. To that end, the coordinates of the SMAD7 and LATS2 peptides were extracted from the YAP^WW1^-bound structures (PDB 2ltw and 32dh, respectively). Additionally, we artificially created a linear starting conformation of SMAD7, as well as peptides **6** and **7**, in such a way as to resemble the conformation of the LATS2 peptide. Prior to the simulation, protein structures were prepared using Schrödinger’s Protein Preparation Wizard (release 2025-1, Cambridge, MA, USA), with protonation states of titratable residues assigned to a pH of 7.4 based on PROPKA calculations [39]. The proline peptide bond cis/trans isomerization proceeds over high activation barriers and is not sampled on microsecond MD timescales; furthermore, to our knowledge, cis-Pro states have not been observed for these peptides in available experimental structural data. For this reason, all simulations were initiated in the experimentally observed trans configuration, and the resulting ensembles reflect this isomerization state.

For MD simulations, the GROMACS 2024.2 suite [40,41,42] was used with the Amber 99SB-ILDNP force field [43] in combination with the explicit TIP3P water model [44]. The protein system was centered in a dodecahedral box, at least 10 Å away from any edge, solvated, and the charge was neutralized with NaCl and further adjusted to a salt concentration of 0.15 M. The system was minimized using the steepest descent algorithm (maximal force of 500 kJ mol^−1^ nm^−1^) before equilibration. The system was then heated to 298 K over 0.1 ns and equilibrated for 0.2 ns using the v-rescale thermostat [45] (NVT ensemble). At this stage, positional restraints were applied to all protein atoms (force constant of 1000 kJ mol^−1^ nm^−2^). During 2.5 ns of NPT equilibration, where the pressure was kept at 1 bar using the Berendsen barostat [46], the restraints were gradually reduced to 5 kJ mol^−1^ nm^−2^. The system was modeled under periodic boundary conditions, with the electrostatic interactions treated with the particle mesh Ewald method [47]. The short-range nonbonded interactions were calculated with a cutoff of 10 Å, and bonds were constrained using the LINCS algorithm [48]. An integration step of 2.0 fs was used. Unrestrained production simulations were run in the NPT ensemble with the Parrinello-Rahman barostat [49], with the system coordinates saved every 0.1 ns. All simulations were 1 μs long (or 2 μs for the Boltz-2-derived models of TAZ^WW^-SMAD7 and TAZ^WW^-LATS2 complexes to allow for more sampling in the absence of experimentally derived structures) and were performed in triplicate. The trajectories were analyzed using GROMACS tools v 2005.5 and custom Python v3.13 scripts.

## 3. Results and Discussion

YAP^WW1^ and TAZ^WW^ are small protein modules (Figure 1A), whose properties may be influenced by tags typically added to proteins for use in biochemical or biophysical assays. Consequently, three different protein constructs were created for each WW domain: Avi-tagged at the N-terminus, Avi-tagged at the C-terminus, and untagged protein (Supplementary Figure S3A). These six protein variants were purified to homogeneity (Supplementary Figure S3B) for subsequent biochemical and structural analyses.

The presence of two tryptophan residues within the WW domains enables measurement of their thermal stability by monitoring changes in intrinsic fluorescence at 330 nm upon excitation at 280 nm (Supplementary Figure S4). These fluorescence changes reflect alterations in the environment surrounding the tryptophan residues during unfolding. Consistent with previous findings for YAP^WW1^ [50], the melting curves of the WW domain constructs can be fitted with a two-state folding model from which a melting temperature (T_m_) was determined for each construct (Table 1). The presence of an Avi-tag at the N-terminus of YAP^WW1^ has the largest effect on thermal stability (ΔT_m_ = T_m_^tagged^ − T_m_^untagged^ = 2.8 °C). In contrast, the effect of the tag on T_m_ is less pronounced in the other constructs. Notably, our results reveal that TAZ^WW^ displays a higher T_m_ compared to YAP^WW1^, with an 8.3 °C difference observed between the untagged proteins. Consequently, under our experimental conditions, TAZ^WW^ is substantially more stable than YAP^WW1^.

**Figure 2:**
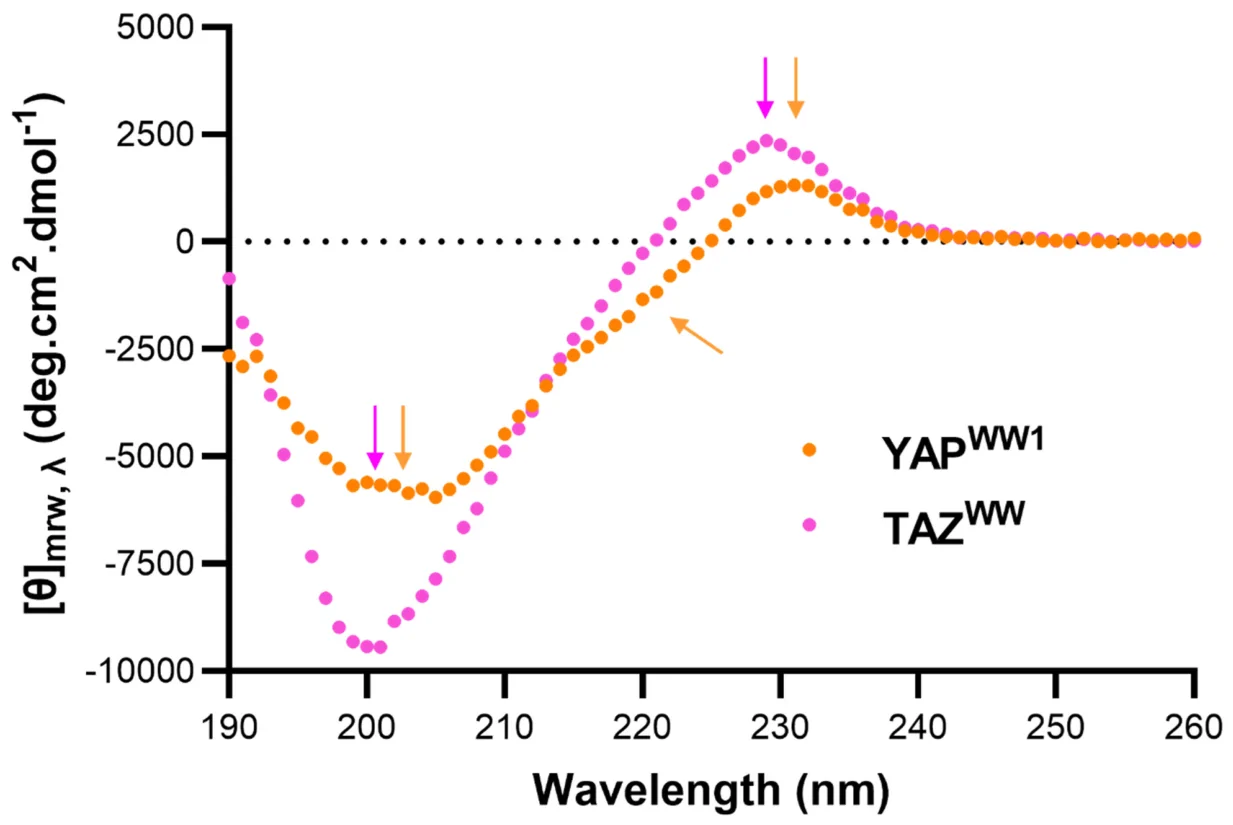
Analysis of YAP^WW1^ and TAZ^WW^ using far-UV circular dichroism. The spectra of YAP^WW1^ and TAZ^WW^ measured at 25 °C are overlaid. The arrows indicate the position of the bands mentioned in the text.

The structure of the different constructs was studied using far-ultraviolet circular dichroism (CD). The CD spectra were found to be similar for the three constructs of each protein, indicating that the Avi-tag has no major effect on YAP^WW1^ or TAZ^WW^ (Supplementary Figure S5A). The CD spectra obtained at 25 °C and 4 °C for the two untagged proteins were also similar (Supplementary Figure S5B). However, when the CD spectra obtained at 25 °C were superimposed, some differences between untagged YAP^WW1^ and TAZ^WW^ became evident (Figure 2). Both proteins exhibited a positive band around 230 nm (λ_max_ TAZ^WW^ 229 nm; λ_max_ YAP^WW1^ 231 nm), which is associated with the contribution of the tryptophan residues [51], but the intensity of this band was higher for TAZ^WW^. A negative band around 220 nm, which is more prominent in YAP^WW1^, corresponds to a β-sheet contribution [52]. Additionally, an intense negative band around 201 nm (λ_min_ TAZ^WW^ 200 nm; λ_min_ YAP^WW1^ 202 nm) was present, indicating a random coil contribution [52]. This band appeared less intense and exhibited a broader minimum region in YAP^WW1^. These CD spectra resemble those obtained with other WW domains (see [22,30]), and the presence of bands at 201 and 230 nm is consistent, under our experimental conditions, with partially folded ensembles of YAP^WW1^ and TAZ^WW^.

**Figure 3:**
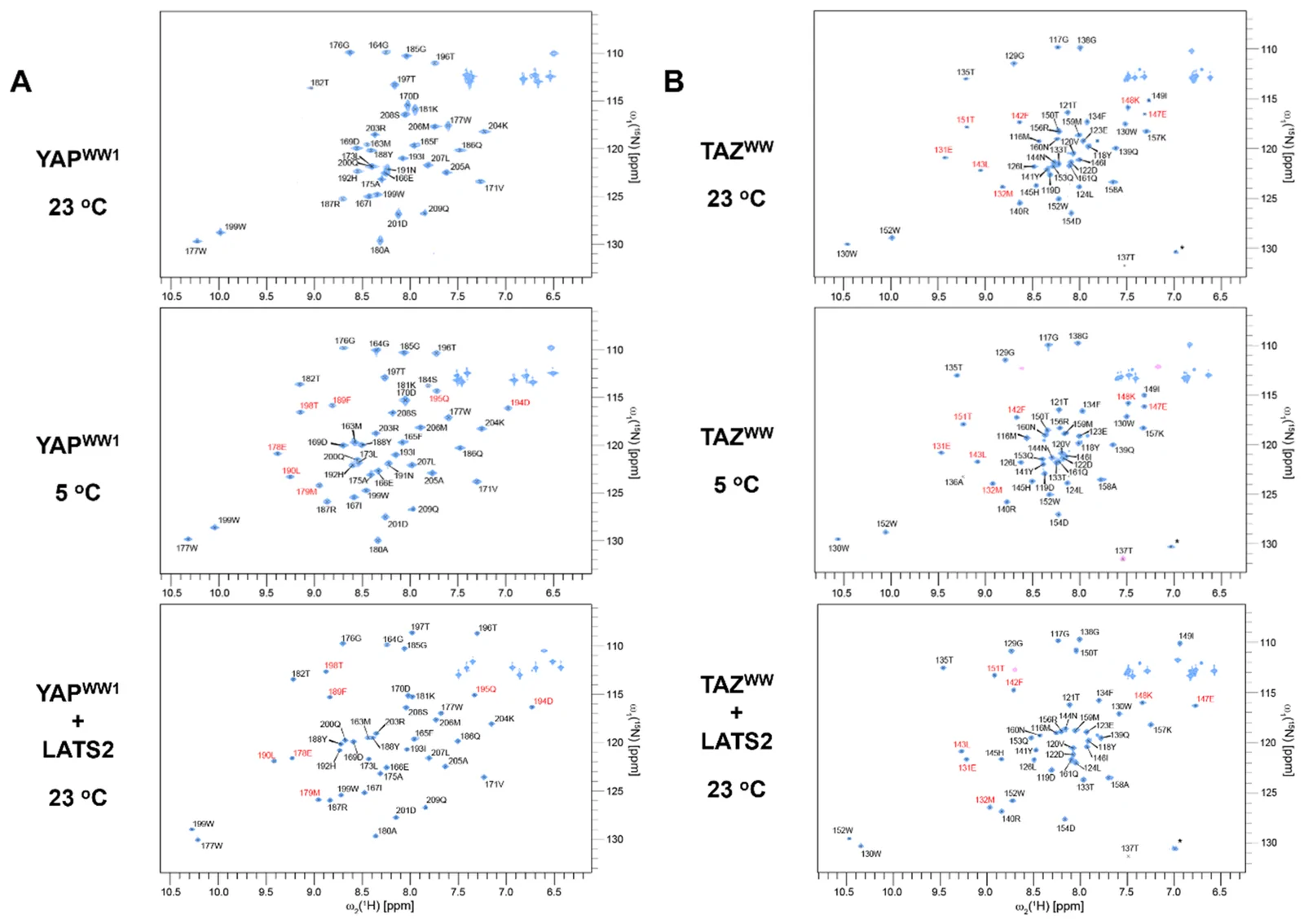
Nuclear magnetic resonance study of YAP^WW1^ and TAZ^WW^. (**A**) 2D ^15^N-HSQC spectra of ^13^C,^15^N-labeled YAP^WW1^. (**B**) 2D ^15^N-HSQC spectra of ^13^C,^15^N-labeled TAZ^WW^. All spectra were recorded under identical conditions using a 100 µM protein concentration, transformed and plotted identically (positive contours in blue, negative contours in magenta). Top panel: experiments at 23 °C without peptide. Middle panel: experiments at 5 °C without peptide. Bottom panel: experiments at 23 °C in the presence of 800 μM LATS2. In the different spectra, the peaks belonging to assigned backbone and indole amide resonances are labeled with their corresponding residue numbers, otherwise they are marked with an asterisk. Peaks of residues that are broadened beyond detection in apo YAP^WW1^ at 23 °C ((**A**) top panel), as well as their sequence-related residues in TAZ^WW^, are highlighted with red labels in the corresponding panels.

Nuclear magnetic resonance (NMR) experiments with YAP^WW1^ and TAZ^WW^ were conducted to gain further insight into the structural properties of these two isolated WW domains. As a basis for these studies, backbone resonance assignments were performed for both ^13^C,^15^N-labeled proteins (Figure 3). For YAP^WW1^ at 5 °C and for the YAP^WW1^/peptide complexes at 23 °C, we achieved backbone amide resonance assignments for 42 of 43 expected residues (98%). Unfavorable spin relaxation prevented us from assigning residue Ser183^YAPWW1^. For YAP^WW1^ at 23 °C, 35 of 43 (81%) of the expected backbone amide resonances were assigned as seven additional peaks were broadened beyond detection by conformational exchange, corresponding to residues Glu178^YAPWW1^, Met179^YAPWW1^, Phe189^YAPWW1^, Leu190^YAPWW1^, Asp194^YAPWW1^, Gln195^YAPWW1^ and Thr198^YAPWW1^. For TAZ^WW^ at 5 °C and for the TAZ^WW^/peptide complexes at 23 °C, we achieved complete amide resonance assignments. For TAZ^WW^ at 23 °C, 42 of 43 (98%) of the expected backbone amide resonances were assigned. Unfavorable spin relaxation prevented us from assigning residue Ala136^TAZWW^.

In YAP^WW1^, the peaks corresponding to group-1 residues [Asp194^YAP^, Gln195^YAP^] and group-2 residues [Glu178^YAP^, Met179^YAP^, Phe189^YAP^, Leu190^YAP^, Thr198^YAP^] were not visible in the 2D ^15^N-HSQC spectrum obtained with ^13^C,^15^N-labeled YAP^WW1^ at 23 °C (Figure 3A, upper panel). However, these peaks became visible at 5 °C (Figure 3A, middle panel, residues labeled in red). This observation suggests that at 23 °C these seven residues are in an intermediate exchange state on the NMR time scale between different conformations, which broadens the NMR signals beyond detection. This could imply a dynamic equilibrium between folded and partially unfolded forms of YAP^WW1^. The group-1 residues are in the loop (residues 191–195) connecting the β2 and β3 strands, while the group-2 residues are part of the three-stranded antiparallel β-sheet, which is the main structural element of the WW domain (Supplementary Figure S6). Hence, the core of YAP^WW1^ exhibits significant conformational flexibility. Webb et al. also reported weaker peaks in the ^15^N-HSQC spectra of YAP^WW1^, either isolated or in tandem with YAP^WW2^, which they attributed to conformational exchange between different forms of this domain [26]. These findings are also reminiscent of a molecular dynamics simulation study, in which the unfolding of YAP^WW1^ starts with the disruption of a hydrogen bond between β2 and β3 [53].

The 2D ^15^N-HSQC spectrum obtained with TAZ^WW^ closely resembles that of YAP^WW1^, as expected from their high sequence homology. However, the backbone amide peaks corresponding to the group-1 and group-2 residues, which were not visible in the YAP^WW1^ spectrum at 23 °C (Figure 3A, upper panel), are visible at this temperature in TAZ^WW^ (peaks corresponding to group-1 residues [Glu147^TAZ^, Lys148^TAZ^] and group-2 residues [Glu131^TAZ^, Met132^TAZ^, Phe142^TAZ^, Leu143^TAZ^, Thr151^TAZ^]) although they appear broader compared to the other amide peaks in the spectrum (Figure 3B, upper panel, residues labeled in red).

The NMR experiments conducted at 23 °C suggest that YAP^WW1^ and TAZ^WW^ are dynamic protein modules that exist in solution in an equilibrium between different conformations and that the exchange between these conformations at 23 °C differs between YAP^WW1^ and TAZ^WW^. These data are consistent with a model in which TAZ^WW^ is more stable than YAP^WW1^ at room temperature, with a higher population of folded conformations in TAZ^WW^ relative to YAP^WW1^.

We next measured the interaction between YAP^WW1^/TAZ^WW^ and different peptides containing a PPxY motif (Figure 1B). These peptides are derived from human proteins and their binding to YAP^WW1^ has been previously studied [19,22]. However, to our knowledge, their affinity for TAZ^WW^ (except for LATS1_1 (Kd = 15 μM) and LATS1_2 (Kd = 4.2 μM) [27]) has not been reported previously. The cysteine residue in the LATS2 peptide becomes oxidized upon storage in DMSO, but this process is reversible in the presence of the reducing agent tris(2-carboxyethyl)phosphine (TCEP) present in our assays. Nevertheless, we also tested a mutant version of the LATS2 peptide (mLATS2), in which the cysteine is replaced by a serine. The biotinylated-Avi-tagged constructs of the WW domains were immobilized on streptavidin-coated sensor chips for the surface plasmon resonance (SPR) experiments. We tested both N- and C-terminal Avi-tag constructs for each WW domain, as the position of the Avi-tag could influence the interaction with the peptides (Supplementary Figure S7). We found that both constructs bind to the peptides with comparable affinity (Table 2), showing that in SPR the position of the Avi-tag has a minimal effect on the interaction. The comparable affinity of LATS2 and mLATS2 for YAP^WW1^/TAZ^WW^ indicates that LATS2 is stable under our assay conditions.

Schuchardt et al., who measured the heat capacity changes (ΔC_p_) associated with the binding of different peptides to YAP^WW1^, showed that it is partially unstructured in solution and adopts its folded structure upon peptide binding [22]. Since this change in conformation resembles our NMR data obtained at different temperatures, we investigated the effect of LATS2 binding on YAP^WW1^ and TAZ^WW^. Binding of the LATS2 peptide induces significant sharpening of peaks in the ^15^N-HSQC spectrum. In particular, the peaks corresponding to the seven residues that were previously invisible in the ^15^N-HSQC spectrum of ^13^C,^15^N-labeled YAP^WW1^ at 23 °C become visible in the presence of the peptide at this temperature (Figure 3A, lower panel). A similar sharpening of peaks is observed in ^13^C,^15^N-labeled TAZ^WW^ upon peptide binding, albeit to a lesser extent than for YAP^WW1^ (Figure 3B, lower panel). These findings, consistent with the results obtained by Schuchardt et al., suggest that the binding of LATS2 stabilizes YAP^WW1^/TAZ^WW^ in a folded conformation.

The affinities of the peptides range from single to triple-digit micromolar, with more than a 10-fold difference between LATS2 (the most potent peptide) and SMAD7 (the least potent peptide, Table 2). As LATS2 and SMAD7 share the same PPPPY central region with different flanking residues (Figure 1B), the different affinity of these peptides for YAP^WW1^/TAZ^WW^ should be attributed to residues located at either their N-terminus and/or C-terminus. To check this hypothesis, we replaced the SMAD7 residues located at the N- (**1**) or C-terminus (**2**) of the PPPPY region with the corresponding LATS2 residues (Figure 1C, Table 3). The presence of the LATS2 residues at the C-terminus of SMAD7 moderately decreases K_d_ (**2** YAP^WW1^ 1.6-fold; TAZ^WW^ 3.4-fold, Table 3), while the mutation of the N-terminus provides a more significant gain in affinity (**1** YAP^WW1^ 5.9-fold; TAZ^WW^ 8.5-fold, Table 3). This indicates that the different affinity between LATS2 and SMAD7 for YAP^WW1^/TAZ^WW^ mainly arises from the three residues located at the N-terminus of the PPPPY region. These amino acids were individually replaced in SMAD7 by the corresponding LATS2 residues (Peptides **3–5**, Figure 1C). Each mutation improves K_d_ (Table 3), but the largest effect was observed with **4**, suggesting that either the presence of an arginine (Arg512^LATS2^) and/or the absence of a glutamate (Glu205^SMAD7^) at the second position in these peptides is important for the different affinity between SMAD7 and LATS2.

**Figure 4:**
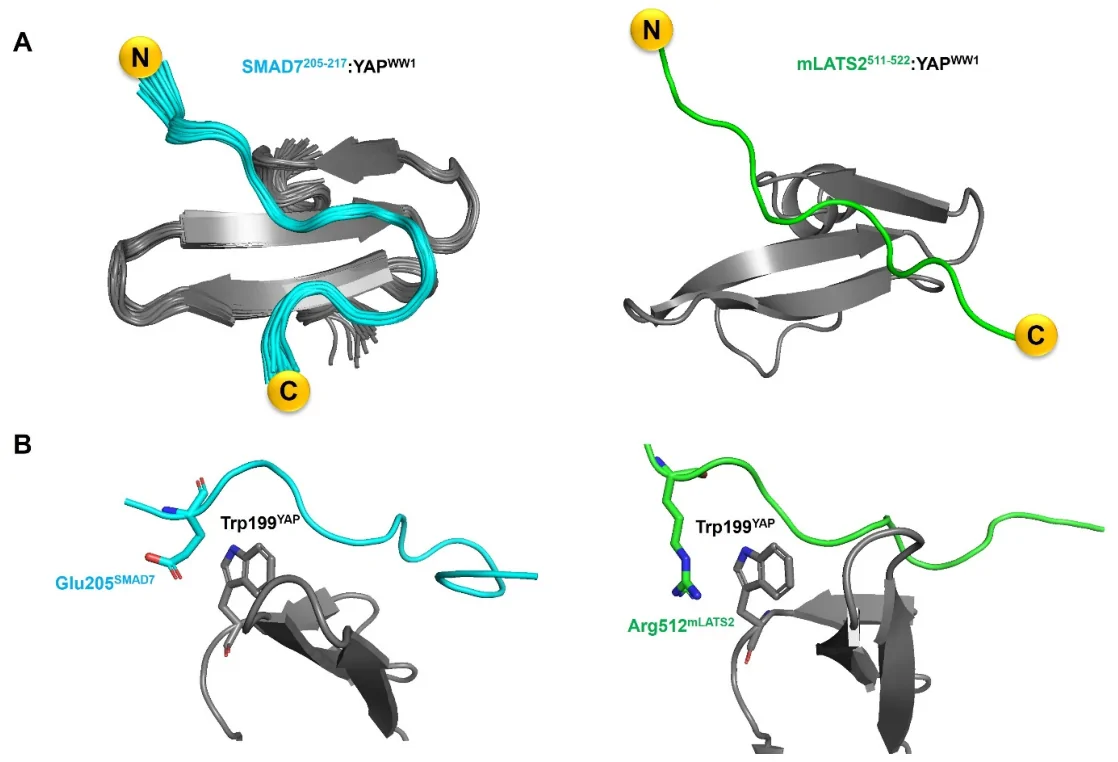
Structural details of SMAD7 and LATS2 binding to YAP^WW1^. (**A**) The structures of the SMAD7:YAP^WW1^ (PDB 2ltw) and mLATS2:YAP^WW1^ (PDB 32dh) complexes are displayed in the same orientation. Thirty different conformers obtained by NMR for the SMAD7:YAP^WW1^ complex are represented. The N– and C–termini of SMAD7/mLATS2 are indicated. (**B**) Close-up view of the region surrounding Glu205^SMAD7^–Trp199^YAPWW1^ in the SMAD7:YAP^WW1^ complex (left panel) and Arg512^LATS2^-Trp199^YAPWW1^ in the mLATS2:YAP^WW1^ complex (right panel). The guanidinium moiety of Arg512^LATS2^ is located 3.5 Å away from the indole ring of Trp199^YAPWW1^.

The structure of the SMAD7:YAP^WW1^ complex has been previously determined (PDB 2ltw [54]), whereas the structure of the LATS2:YAP^WW1^ complex had not been reported prior to this work. Therefore, we have elucidated the structure of this complex using X-ray crystallography. Our high-resolution structure (1.03 Å) provides the experimentally observed reference conformation and enables the direct comparison between the two peptide binders, revealing a significant difference in the binding conformation of mLATS2 and SMAD7 to YAP^WW1^. The SMAD7-bound form assumes an Ω-like shape, whereas bound mLATS2 adopts an extended conformation (Figure 4A). This bound conformation of mLATS2 is similar to the one observed by NMR for a LATS1 derived peptide (PDB 5ydx [27]) and for another peptide carrying a PPxY motif (PDB 1jmq [55]). Despite this difference, the residues of the PPxY motif from mLATS2/SMAD7 occupy a similar position at the binding interface (Supplementary Figure S8). The guanidinium group of Arg512^mLATS2^ and the indole ring of Trp199^YAP^ are positioned 3.5 Å apart potentially forming a cation-pi interaction (Figure 4B). However, this interaction alone does not account for the higher potency of LATS2, as LATS1_2, which shares a high sequence homology with LATS2 (nine conserved residues) but lacks an arginine at this position (it contains glutamine instead) (Figure 1B), exhibits a similar potency (~3-fold difference, Table 2). Hence, the difference in binding affinity to YAP^WW1^ between LATS2 and SMAD7 may result from a favorable contribution to binding from the guanidinium group of Arg512^LATS2^ as well as from an unfavorable effect from the carboxylic moiety of Glu205^SMAD7^ located near the electron-rich π system of the indole ring of Trp199^YAP^ (Figure 4B).

**Figure 5:**
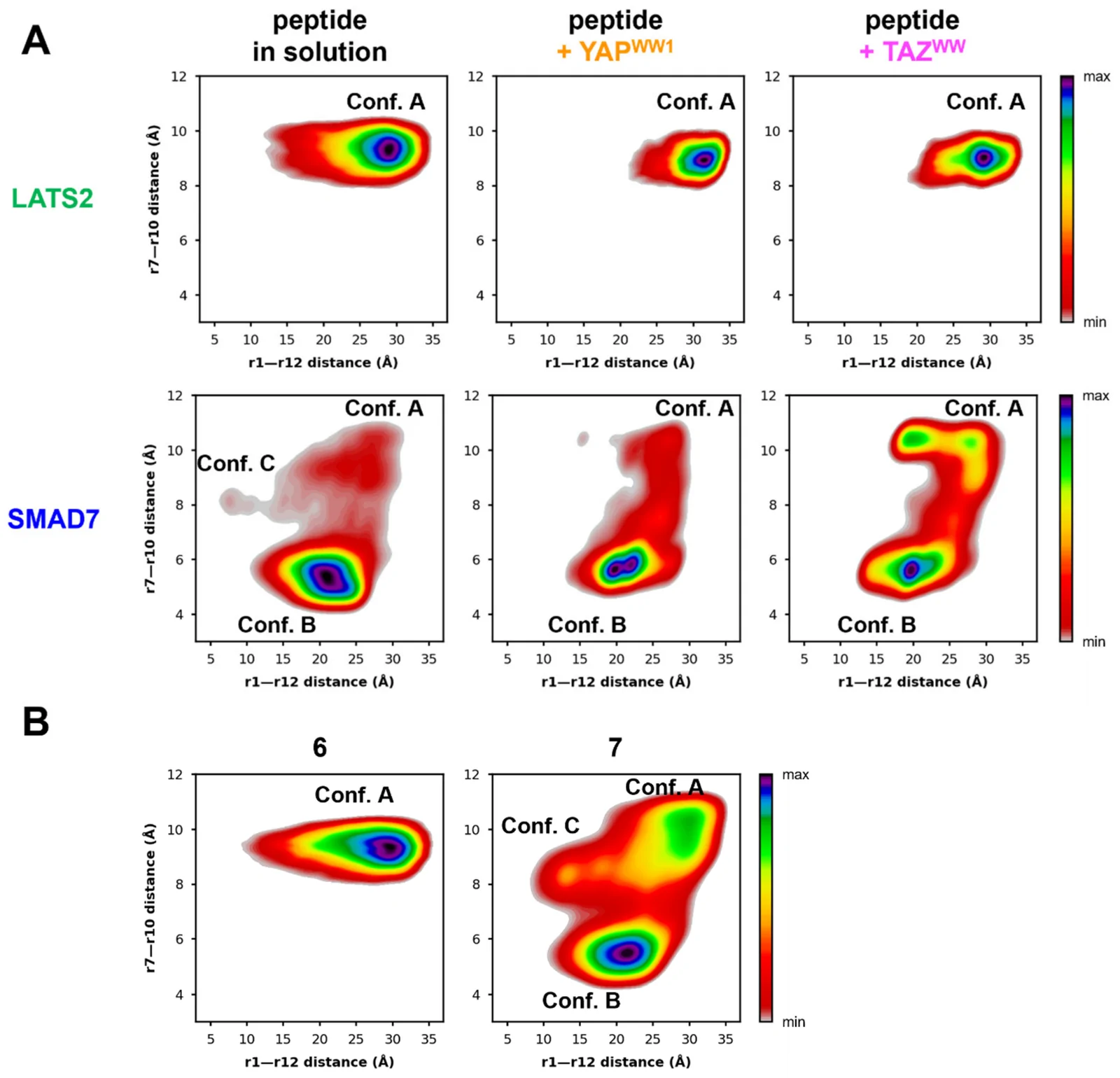
Peptide conformational flexibility from MD simulations. (**A**) Conformational sampling of the SMAD7 and LATS2 peptides in solution and when bound to YAP^WW1^ and TAZ^WW^. (**B**) Conformational flexibility of peptides **6** and **7** in solution. The x-axis shows the N-to-C terminal distance (peptide residues 1 and 12), measured between the Cα atoms of the corresponding amino acids. The y-axis shows the distance between residues 7 and 10, reporting on the narrow gap that defines the Ω-conformation. Each system was simulated in three independent 1 µs replicas (extended to 2 µs for the TAZ^WW^-bound co-folding-modeled complexes). Plots combine data across all replicas, and sampling of the three individual replicas for each system is shown in Supplementary Figure S10. The color scale reflects sampling density, with red indicating rarely sampled regions and blue showing conformational states most frequently visited. Specific conformational states are labeled as: Conformation A, where both r1–r12 and r7–r10 distances are maximal, corresponding to the extended conformation seen in the LATS2 crystal structure (PDB 32dh); Conformation B, where both distances are shorter, matching the Ω-conformation in the SMAD7 NMR structure (PDB 2ltw); and Conformation C, a rarely sampled state with a compressed N-to-C distance and an intermediate r7–r10 separation.

We next ran molecular dynamics (MD) simulations of the SMAD7 and LATS2 peptides in solution and in complex with YAP^WW1^ or TAZ^WW^. Starting from the YAP^WW1^-bound state, simulations of the LATS2 peptide in solution suggested that it adopts a conformation with large distance values between residues 1–12 and residues 7–10 (conformation A, top left panel, Figure 5A), corresponding to a linear conformation. LATS2 undergoes negligible conformational change upon binding to either YAP^WW1^ or TAZ^WW^ (top middle and right panels, Figure 5A), suggesting that this peptide is conformationally preorganized in solution. Starting from the YAP^WW1^-bound Ω-like conformation of SMAD7, MD simulations indicated that the peptide is more flexible in solution, sampling a conformation similar to conformation A observed for LATS2, as well as two additional states with smaller distance values between residues 1–12 and residues 7–10: an Ω-like conformation (conformation B) and an additional state (conformation C) (bottom left panel, Figure 5A). To assess whether the starting conformation affects MD sampling, we also performed simulations starting from an artificially generated linear conformation of SMAD7. In both the linear and crystallographic (Ω-like) starting states, the MD trajectories rapidly converged toward sampling predominantly the Ω-like configuration (Supplementary Figure S9). Although the choice of a biomolecular force field can affect the behavior of highly flexible peptides, the convergence of distinct starting structures toward similar ensembles indicates that the qualitative dynamical trends observed are robust under our simulation conditions. Binding of SMAD7 to either YAP^WW1^ or TAZ^WW^ further narrows this conformational ensemble (bottom middle and right panels, Figure 5A), with the bound peptide populating conformational states A and B. Because the TAZ^WW^-bound simulations were initiated from Boltz-2 predicted complexes rather than experimentally determined structures (like the YAP^WW1^ complexes), comparisons between the two systems should be interpreted with appropriate caution. Nevertheless, the high-confidence predictions and close structural agreement with the experimentally determined YAP^WW1^ complexes support the plausibility of the observed conformational behavior.

The amino acids located at the peptide-binding site are well conserved between YAP^WW1^ and TAZ^WW^, particularly in the region between the two tryptophans (Figure 1A), suggesting that they should bind peptides containing a PPxY motif with similar affinity. Indeed, we observe only a small difference in affinity between the two WW domains, approximately 2- to 3-fold (Table 2). However, we noticed that all peptides, excepted LATS1_1, exhibit lower affinity for TAZ^WW^. This difference does not appear to result from bias in our assays, as Verma et al. also reported a 2-fold lower affinity of LATS1_2 for TAZ^WW^ [27]. A larger difference was observed for SMAD7, which binds 4.4-fold more strongly to YAP^WW1^ than to TAZ^WW^ (Table 3). Since **1** binds better to YAP^WW1^ than to TAZ^WW^ (3.0-fold), and **2** behaves similarly to LATS2 (only 2.1-fold; Table 3), the more pronounced preference of SMAD7 for YAP^WW1^ is attributable primarily to the residues following the PPPPY region. Taken together, these data, along with the high sequence homology between YAP^WW1^ and TAZ^WW^, suggest that this difference arises, at least in part, from intrinsic properties of SMAD7.

MD simulations indicate that the LATS2 peptide is largely preorganized in solution into a linear conformation that closely resembles its YAP^WW1^-bound state. As a result, it likely incurs a smaller conformational reorganization penalty upon binding than SMAD7, which is more flexible in solution but adopts an Ω-loop-like conformation when bound. Because YAP^WW1^ is more conformationally dynamic than TAZ^WW^, SMAD7 may partially compensate for its larger conformational penalty through a mechanism akin to mutually synergistic folding [56,57]. This dynamic compatibility provides a plausible explanation for the modest preference of SMAD7 for YAP^WW1^. Based on the experimental affinity data for peptides **1** and **2**, which indicate that the selectivity originates predominantly from the peptide C-terminus, we sought to probe the role of peptide flexibility through backbone preorganization.

To do so, we designed two additional mutant peptides (**6** and **7**, Figure 1C) based on the Pro/Ser substitutions. Although this swap preserves charge and approximate side-chain volume, proline and serine differ substantially in backbone rigidity and hydrogen-bonding capacity. While proline’s pyrrolidine ring imposes a strong geometric constraint on backbone dihedral angles, therefore making conformational preorganization the dominant effect of this mutation, serine’s hydrogen-bonding capacity may also affect local non-covalent interactions. The Ser212Pro mutation in SMAD7 (**6**) introduces a conformationally restricting proline that markedly reduces sampling of the Ω-like conformation in solution, as shown by our MD simulations, resulting in an ensemble resembling that of LATS2 (left panel, Figure 5B). As predicted, this rigidification reduces the difference in affinity, with the SPR-determined K_d_^TAZ^/K_d_^YAP^ ratio dropping from 4.4 for wild-type SMAD7 to only 2.3 for **6** (Table 3). This result is consistent with the interpretation that preorganized linear peptides like LATS2 and **6** bind in a manner that does not strongly depend on receptor flexibility. Conversely, the reciprocal Pro519Ser substitution in LATS2 (**7**) increases peptide flexibility in our MD simulations, allowing the peptide to access Ω-like states and thereby moving its ensemble partway toward SMAD7-like behavior (right panel, Figure 5B). Although the conformational populations derived from 1-µs simulations are only semiquantitative, and should be interpreted cautiously, they nonetheless indicate that the shift is incomplete: peptide **7** retains a substantial linear population and samples additional conformations not prominent in SMAD7. As a result, **7** occupies a broader and more heterogeneous conformational ensemble than either parent peptide. Consistent with this mixed solution behavior, the SPR experiments show that binding selectivity of **7** (K_d_^TAZ^/K_d_^YAP^ = 2.4, Table 3) is more similar to that of LATS2 than SMAD7. We interpret this as reflecting the residual linear population of **7**, which could continue to support binding that is less sensitive to WW-domain flexibility and therefore does not reproduce the stronger selectivity characteristic of SMAD7.

Our results suggest a plausible mechanistic link between peptide conformational plasticity, intrinsic YAP/TAZ WW domain stability, and differences in affinity. Under our experimental conditions, rigid and more preorganized peptides bind more tightly and with less discrimination between YAP^WW1^ and TAZ^WW^, whereas flexible peptides bind less tightly overall and show greater sensitivity to receptor dynamic. In this framework, the lower stability and partially folded ensemble of YAP^WW1^ may allow flexible peptides such as SMAD7 to distribute the conformational cost of binding across both partners, while the more rigid TAZ^WW^ may be less able to reciprocate this adaptability, penalizing binding partners that require significant conformational reorganization to adopt their bioactive state. This mechanistic interpretation is consistent with the observed trends, although additional thermodynamic and kinetic could be conducted to assess it more directly. Finally, although experimental structures of TAZ^WW^-peptide complexes are not currently available, the high-confidence Boltz-2 models used here closely resemble the corresponding experimentally determined YAP^WW1^ complexes, supporting the robustness of the overall mechanistic interpretation.

## 4. Conclusions

In summary, despite their high sequence similarity and conserved binding interfaces, YAP^WW1^ and TAZ^WW^ exhibit distinct biophysical properties that influence ligand recognition. TAZ^WW^ is intrinsically more stable and predominantly folded, whereas YAP^WW1^ samples a broader conformational ensemble. While both domains bind PPxY-containing peptides with comparable micromolar affinities, differences emerge for conformationally flexible ligands. Our combined structural, biophysical, and computational analyses suggest that peptide binding might be shaped by the interplay between ligand preorganization and receptor dynamics. The rigid LATS2 peptide binds with similar affinity to both WW domains, whereas the flexible SMAD7 shows higher affinity for YAP^WW1^, which could potentially be attributed to its greater conformational adaptability. These findings reflect qualitative trends, and are hence a plausible mechanistic framework linking WW domain stability to ligand conformational plasticity to help explain subtle selectivity in ligand recognition by YAP and TAZ WW domains in the absence of major sequence differences at the binding site. More broadly, these findings illustrate how conformational dynamics can create selective recognition among highly homologous interaction modules even when their binding-site architectures are nearly conserved.

## Figures and Tables

**Table 1:** Thermal denaturation of the WW constructs. The sequences of the different proteins are provided in Supplementary Figure S3A. The thermal denaturation of the proteins was measured by NanoDSF following the change in fluorescence with temperature at 330 nm upon excitation at 280 nm. The values for the melting temperature (T_m_) represent the averages and standard deviations of n ≥ 2 independent experiments.

	Protein T_m_ (°C)	Avi-Protein T_m_ (°C)	Protein-Avi T_m_ (°C)
**YAP^WW1^**	44.5 ± 0.2	47.3 ± 0.6	44.6 ± 0.8
**TAZ^WW^**	52.8 ± 0.7	52.5 ± 0.4	54.0 ± 0.8

**Table 2:** Binding affinity of different peptides for YAP^WW1^ and TAZ^WW^. Peptide sequences are provided in Figure 1B. The affinities (K_d_^eq^) were measured at equilibrium by surface plasmon resonance at 25 °C. YAP and TAZ proteins were immobilized on streptavidin chips via their biotinylated Avi-tag (Avi). Data fitting was performed using the Biacore 8K Insight Evaluation software v 6.0.7.1750 (Cytiva, Marlborough, MA, USA) with a 1:1 binding model. Values represent averages and standard deviations of n ≥ 2 independent experiments.

Peptides	YAP^WW1^-Avi (K_d_^eq^; μM)	Avi-YAP^WW1^ (K_d_^eq^; μM)	TAZ^WW^-Avi (K_d_^eq^; μM)	Avi-TAZ^WW^ (K_d_^eq^; μM)
**LATS1_1**	55 ± 6	63 ± 7	61 ± 4	51 ± 6
**LATS1_2**	16 ± 1	15 ± 2	34 ± 4	25 ± 3
**LATS2**	5 ± 1	5.8 ± 0.5	11 ± 1	12 ± 1
**mLATS2**	11 ± 2	16 ± 2	22 ± 3	21 ± 3
**SMAD7**	66 ± 6	66 ± 6	286 ± 38	180 ± 10
**PTCH1_1**	27 ± 5	27 ± 2	52 ± 5	55 ± 4
**PTCH1_2**	22 ± 1	25 ± 1	78 ± 5	66 ± 2

**Table 3:** Mutations of the SMAD7 peptide. The affinities (K_d_^eq^) of the peptides were measured as described in Table 2. The conserved PPPPY region is underlined, and the mutations in SMAD7 are indicated in bold. ^a^ Values taken from Table 2. The numbers in brackets refer to the ratio between the K_d_^eq^ of the peptide and the K_d_^eq^ of the LATS2 peptide. The values represent the averages and standard deviations of n ≥ 3 independent experiments. The experimental data are provided on Supplementary Figure S12.

Peptide	Sequence	YAP^WW1^-Avi (K_d_^eq^; μM)	TAZ^WW^-Avi (K_d_^eq^; μM)	K_d_^TAZ^/K_d_^YAP^
SMAD7	Ac-LESPPPPYSRYP-NH_2_	66 ± 6 ^a^ (11.0)	286 ± 38 ^a^ (26.3)	4.4 ± 0.7
1	Ac-**RRC**PPPPYSRYP-NH_2_	11.2 ± 0.1 (2.2)	34 ± 5 (3.1)	3.0 ± 0.5
2	Ac-LESPPPPY**PKHL**-NH_2_	39 ± 3 (8)	85 ± 4 (7.6)	2.2 ± 0.2
3	Ac-**R**ESPPPPYSRYP-NH_2_	37 ± 3 (7.4)	124 ± 13 (10.6)	3.3 ± 0.5
4	Ac-L**R**SPPPPYSRYP-NH_2_	26 ± 4 (5.2)	90 ± 13 (8.2)	3.5 ± 0.7
5	Ac-LE**C**PPPPYSRYP-NH_2_	52 ± 6 (10.4)	184 ± 17 (16.4)	3.5 ± 0.5
6	Ac-LESPPPPY**P**RYP-NH_2_	66 ± 3	154 ± 12	2.3 ± 0.2
7	Ac-RRCPPPPY**S**KHL-NH_2_	13.5 ± 0.2	32.1 ± 0.4	2.4 ± 0.0
LATS2	Ac-RRCPPPPYPKHL-NH_2_	5 ± 1 ^a^ (1.0)	11 ± 1 ^a^ (1.0)	2.1 ± 0.3

## Data Availability

All additional data supporting the findings are provided in the Supplementary Information File.

## Supplementary Materials

The following supporting information can be downloaded at https://www.mdpi.com/article/10.3390/biom16091355/s1. Figure S1: Summary of X-ray data collection and refinement statistics. Figure S2: Boltz-2 predicted structures of TAZ^WW^ bound to the SMAD7 and LATS2. Figure S3: Protein constructs and analytics. Figure S4: Thermal denaturation of the different proteins used in this study. Figure S5: Far-UV circular dichroism analysis of the proteins used in this study. Figure S6: The location of unassigned residues in YAP^WW1^. Figure S7: Surface plasmon resonance analysis of peptides binding to YAP^WW1^ and TAZ^WW^. Figure S8: Interactions between the PPxY motifs of SMAD7 and LATS2 and YAP^WW1^. Figure S9: Peptide conformational sampling of the SMAD7 peptide. Figure S10: Peptide conformational flexibility from MD simulations. Figure S11: Analytics of the peptides. Figure S12: Measure of the peptides K_d_.
