# Supplementary material for: Comparing YAPWW1 and TAZWW: Similar Binding Sites but Different Stability and Conformational Dynamics
Source: Biomolecules. 2026 Sep 17;16(9):1355. doi: 10.3390/biom16091355 (PMC13604635; doi:10.3390/biom16091355)
Supplement: Supplementary file 1 [file biomolecules-16-01355-s001.zip › biomolecules-4530516-supplementary.pdf]

Comparing YAP^{WW1} and TAZ^{WW}: Similar Binding Sites but Different Stability and Conformational Dynamics

Clara Merlen [1,†], Yannick Mesrouze [1,†], Suzanne Chau [2], Benjamin A. Diehl [3], Alexandra Hinniger [2], Catherine Zimmermann [1], Marco Meyerhofer [1], Patrizia Fontana [1], Svenya Groebke [1], Jürgen Hinrichs [3], Wassim Abdul Rahman [1], Sascha Gutmann [2], César Fernández [2], Dušan Petrović [3] and Patrick Chène [1,*]

[1] Oncology Disease Area, Novartis Biomedical Research, CH-4056 Basel, Switzerland
[2] Discovery Sciences, Novartis Biomedical Research, CH-4056 Basel, Switzerland
[3] Global Discovery Chemistry, Novartis Biomedical Research, CH-4056 Basel, Switzerland
* Correspondence: patrick_chene@yahoo.com
† These authors contributed equally to this work.

Supplementary Figure S1. Summary of X-ray data collection and refinement statistics. [a] Values as reported in STARANISO. [b] Values as defined in phenix.refine. [c] Values as defined in phenix.table_one.

Supplementary Figure S2. Boltz-2 predicted structures of TAZ^{WW} bound to the SMAD7 and LATS2 peptides are in good agreement with the experimentally resolved YAP^{WW1} complexes. **A.** YAP^{WW1} (dark gray) bound to SMAD7 (dark blue), as observed in PDB 2ltw, aligned to the Boltz-2 predicted structure of TAZ^{WW} (light gray) bound to SMAD7 (cyan), with the overall C-alpha RMSD in the range of 1.08 – 1.12 Å for the five predicted complexes. **B.** YAP^{WW1} (dark gray) bound to LATS2 (dark green), as observed in PDB 32dh, aligned to the Boltz-2 predicted structure of TAZ^{WW} (light gray) bound to SMAD7 (light green), with the overall C-alpha RMSD in the range of 0.30 – 0.50 Å for the five predicted complexes. For clarity, only one predicted structure per Boltz run is shown.

Supplementary Figure S3. Protein constructs and analytics.**A.** Amino acid sequences of the protein constructs used in this study. The sequences corresponding to YAP and TAZ are in orange and magenta, respectively. The Avi-tag is cyan, and the linker regions or additional residues are black. **B.** The different proteins (1 µg) were analyzed by liquid chromatography and mass spectrometry. Representative chromatograms are presented. The calculated theoretical mass (MW_{th}) and the experimental mass (MW_{exp}) measured by mass spectrometry are indicated.

Supplementary Figure S4. Thermal denaturation of the different proteins used in this study. Nano differential scanning fluorimetry (NanoDSF) was utilized to assess the thermal stability of the proteins. Changes in fluorescence at 330 nm (upon excitation at 280 nm) were monitored as a function of temperature using a Prometheus NT.48 device (NanoTemper Technologies GmbH, Germany). The figure displays representative thermograms. The melting temperatures (T_m) measured for the experiments shown on the figure are indicated.

Supplementary Figure S5. Far-UV circular dichroism analysis of the proteins used in this study. **A**. The CD spectra of the three constructs for each WW domain are shown. **B**. The CD spectra obtained at 25 ºC and 4 ºC with the untagged proteins have been overlaid.

Supplementary Figure S6. The location of unassigned residues in YAP^{WW1} at 23 °C is shown on the crystal structure of LATS2:YAP^{WW1} (PDB 32dh). Residues that are broadened beyond detection in the ^{15}N-HSQC spectrum of the apo form of YAP^{WW1} at 23 °C (marked with red labels in the bottom left spectrum) are highlighted in red (see also Figure 3). These include residues $E178^{YAPWW1}$, $M179^{YAPWW1}$, $F189^{YAPWW1}$, $L190^{YAPWW1}$, $D194^{YAPWW1}$, $Q195^{YAPWW1}$, and $T198^{YAPWW1}$. Residue $Ser183^{YAPWW1}$, which is also unobservable in YAP^{WW1} at 5 °C and in the LATS2:YAP^{WW1} complex at 23 °C, is marked in orange. Proline residues are shown in black.

Supplementary Figure S7. Surface plasmon resonance analysis of peptides binding to YAP^{WW1} and TAZ^{WW}. Biotinylated-Avi-tagged forms of YAP^{WW1} and TAZ^{WW} were immobilized on streptavidin-coated chips. Data were analyzed using the Biacore 8K Insight Evaluation software (Cytiva, Marlborough, MA) using a 1:1 binding model to determine equilibrium binding affinities (K_d^{eq}). For each peptide, the left panel shows a representative sensorgram. The peptide concentrations and the regions of the sensorgram selected for K_d^{eq} determination are indicated. The right panel displays the corresponding binding isotherm. The fitted maximal experimental response (R_{max}^{exp}) and the calculated maximal theoretical response (R_{max}^{th}) are provided for the experiment presented on the figure.

Supplementary Figure S8. Interactions between the PPxY motifs of SMAD7 and LATS2 and YAP^{WW1}. The structures of the SMAD7:YAP^{WW1} (blue, PDB 2ltw) and LATS2:YAP^{WW1} (green, PDB 32dh) complexes have been superimposed. For clarity, only YAP^{WW1} from the LATS2:YAP^{WW1} complex (grey) is represented. The residues belonging to the PPxY motif are highlighted.

Supplementary Figure S9. Peptide conformational sampling of the SMAD7 peptide, starting from the artificially created linear conformation that resembles that of the LATS2 peptide. The x-axis shows the N-to-C terminal distance (peptide residues 1 and 12), measured between the Cα atoms of the corresponding amino acids. The y-axis shows the distance between residues 7 and 10, reporting on the narrow gap that defines the Ω-conformation. Three independent MD replicas with 1 μs sampling each were combined in this plot. The color scale reflects sampling density, with red indicating rarely sampled regions and purple showing conformational states most frequently visited. Specific conformational states are labeled as: A, where both r1–r12 and r7–r10 distances are maximal, corresponding to the extended conformation seen in the LATS2 crystal structure (PDB 32dh); B, where both distances are shorter, matching the Ω-conformation in the SMAD7 NMR structure (P DB 2ltw); and C, a rarely sampled state with a compressed N-to-C distance and an intermediate r7–r10 separation.

Supplementary Figure S10. Peptide conformational flexibility from MD simulations. Plots are shown across the three individual replicas for each system. Combined replicas are presented in Figure 5 and Supplementary Figure 9. **A.** LATS2 peptide in solution. **B.** SMAD7 peptide in solution. **C.** LATS2 peptide + YAP^{WW1}. **D.** SMAD7 peptide + YAP^{WW1}. **E.** LATS2 peptide + TAZ^{WW}. **F.** SMAD7 peptide + TAZ^{WW}. **G.** SMAD7 peptide in solution, starting from the artificially created linear conformation that resembles that of the LATS2 peptide. **H.** Peptide 6 in solution. **I.** Peptide 7 in solution.

Supplementary Figure S11. Analytics of the peptides. The chromatograms and the MS spectra are provided. The purity measured by LC is highlighted. The theoretical molecular weight of the peptides (MWth) is indicated.

Supplementary Figure S12. Measure of the peptide K_d. The individual affinity constants measured for the peptides presented on Table 3 are given. The number of duplicates ranges from 3 to 5. The calculated confidence intervals are indicated.

Supplementary Figure S1

**Protein**	**$YAP1^{165\text{-}209}$:$LATS2^{511\text{-}522\_C513S}$**
PDB entry ID	32dh
**Data collection**[a]	
Wavelength (Å)	0.8856033
Space group	P3121
a, b, c (Å)	53.036, 53.036, 37.593
α, β, γ (°)	90.0, 90.0, 120.0
Resolution range (Å)	45.93 – 1.03
Unique reflections	23712
Mean I/sigma (I)	19.3
Completeness ellipoidal	0.943
Redundancy	13.8
R_{merge}	0.055
CC (1/2)	0.999
**Refinement**[b]	
Resolution range (Å)	29.09 – 1.03
R_{work}	0.187
R_{free}	0.192
Number of non-hydrogen atoms	
Macromolecule	455
Solvent	69
r.m.s deviation	
Bond length (Å)	0.010
Bond angles (°)	1.17
Ramachandron plot[c]	
Favored (%)	98
Allowed (%)	2
Outliers (%)	0

Supplementary Figure S2

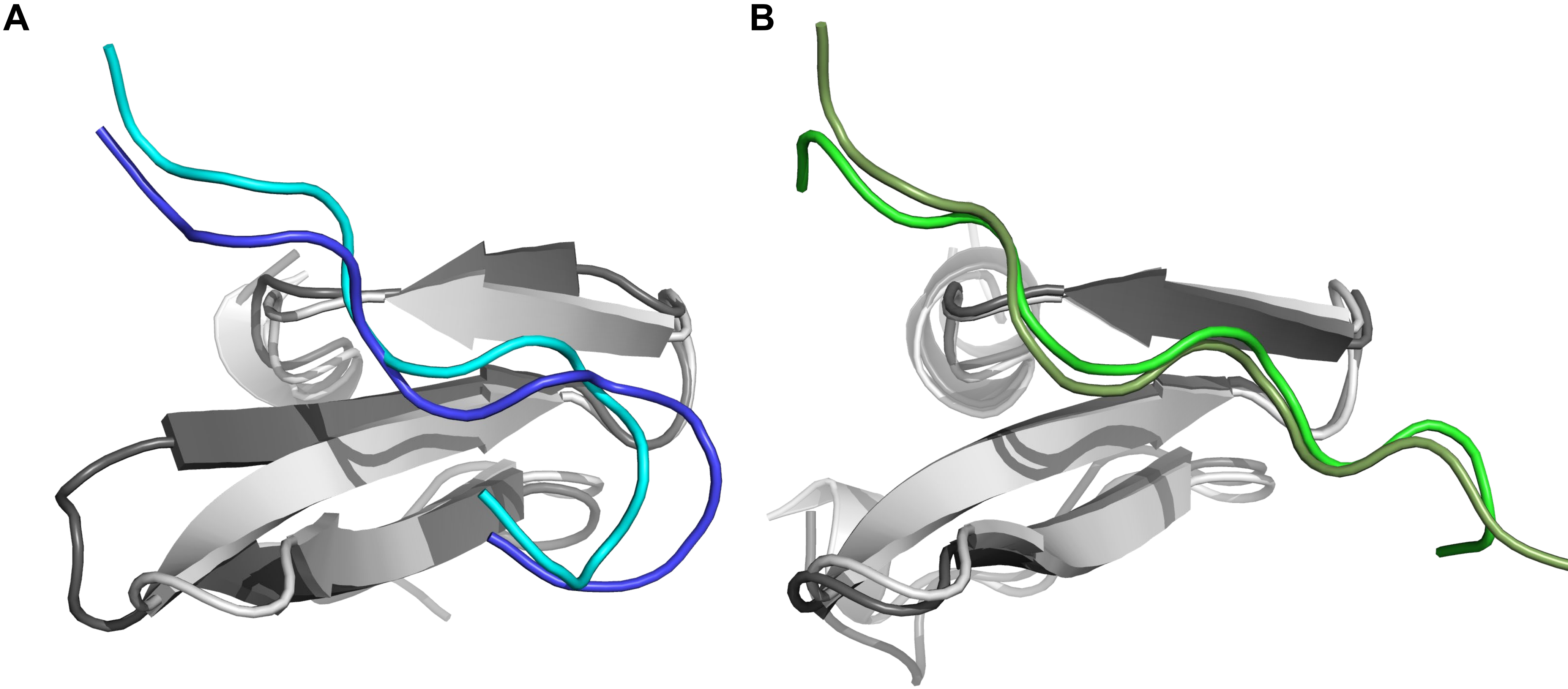

Supplementary Figure S3A

**YAP^{WW1}-Avi**	GPRQSSFEIPDDVPLPAGWEMAKTSSGQRYFLNHIDQTTTWQDPRKAMLSQGGGGSGLNDIFEAQKIEWHE
**Avi-YAP^{WW1}**	GPGLNDIFEAQKIEWHEGSSRQSSFEIPDDVPLPAGWEMAKTSSGQRYFLNHIDQTTTWQDPRKAMLSQ
**YAP^{WW1}**	GAMGFEIPDDVPLPAGWEMAKTSSGQRYFLNHIDQTTTWQDPRKAMLSQ
**TAZ^{WW}-Avi**	GPRQQSYDVTDELPLPPGWEMTFTATGQRYFLNHIEKITTWQDPRKAMNQPGGGGSGLNDIFEAQKIEWHE
**Avi-TAZ^{WW}**	GPGLNDIFEAQKIEWHEGSSRQQSYDVTDELPLPPGWEMTFTATGQRYFLNHIEKITTWQDPRKAMNQP
**TAZ^{WW}**	GAMGYDVTDELPLPPGWEMTFTATGQRYFLNHIEKITTWQDPRKAMNQP

Supplementary Figure S3B

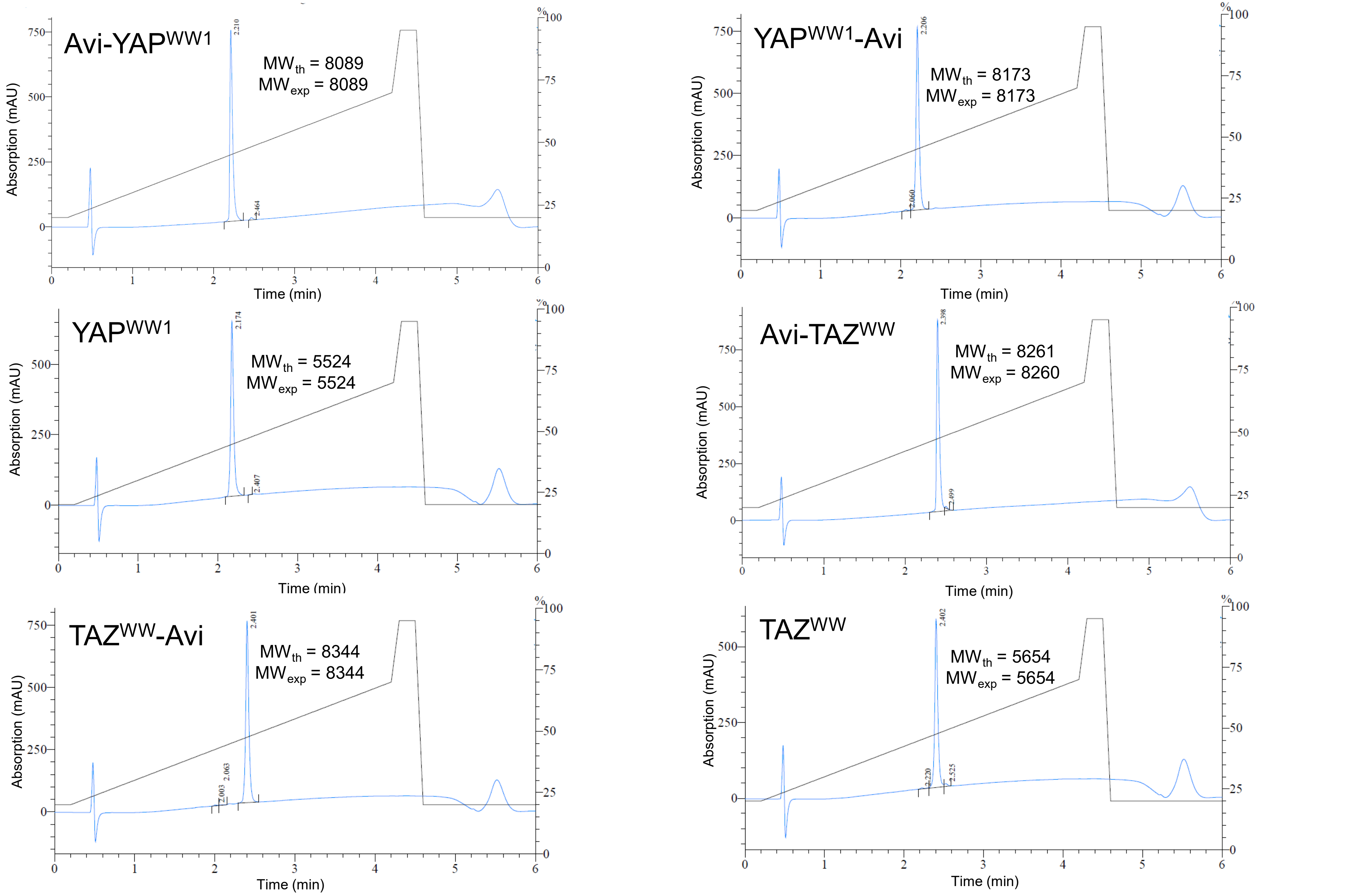

Supplementary Figure S4

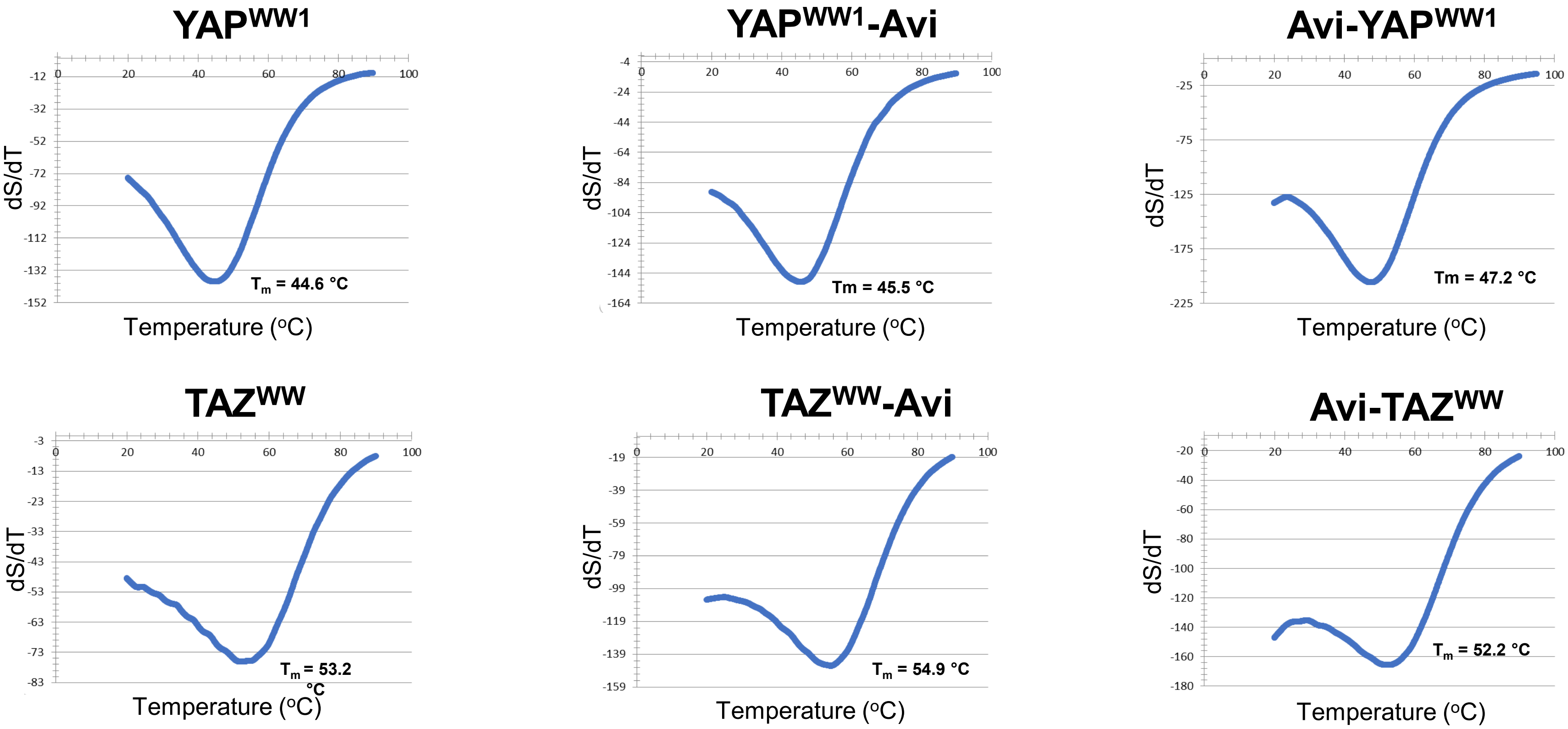

Supplementary Figure S5

A

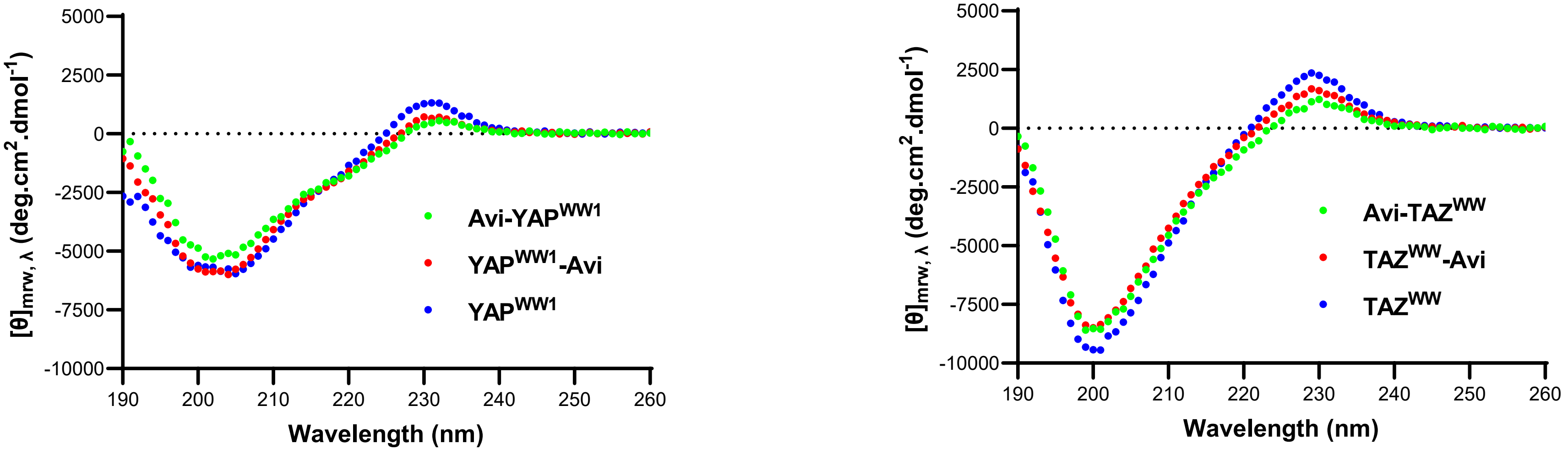

B

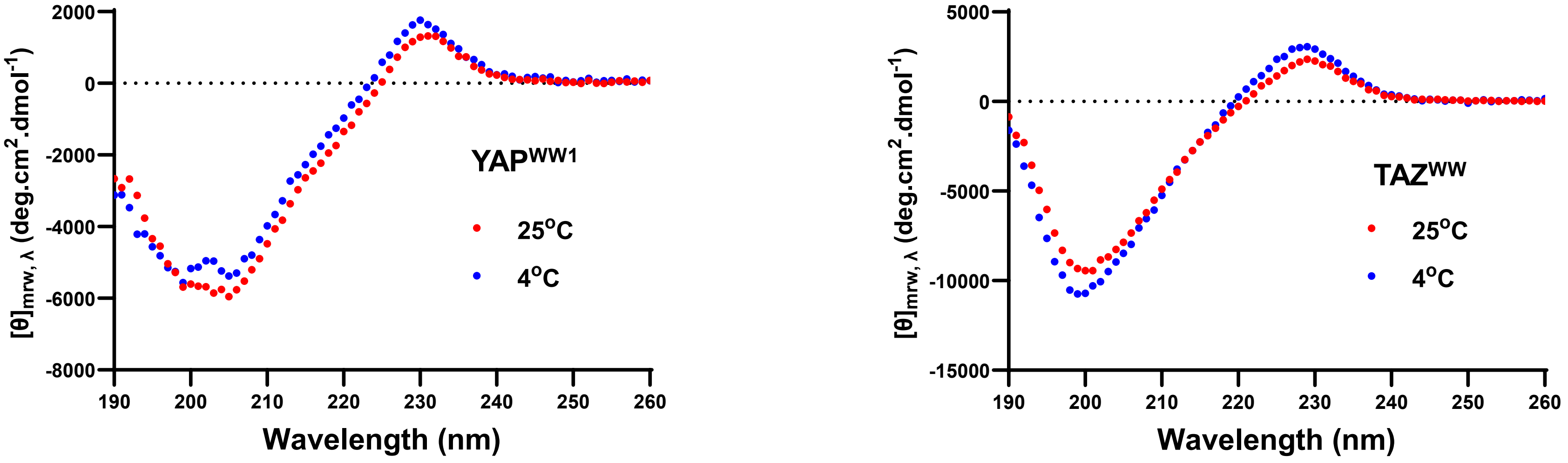

Supplementary Figure S6

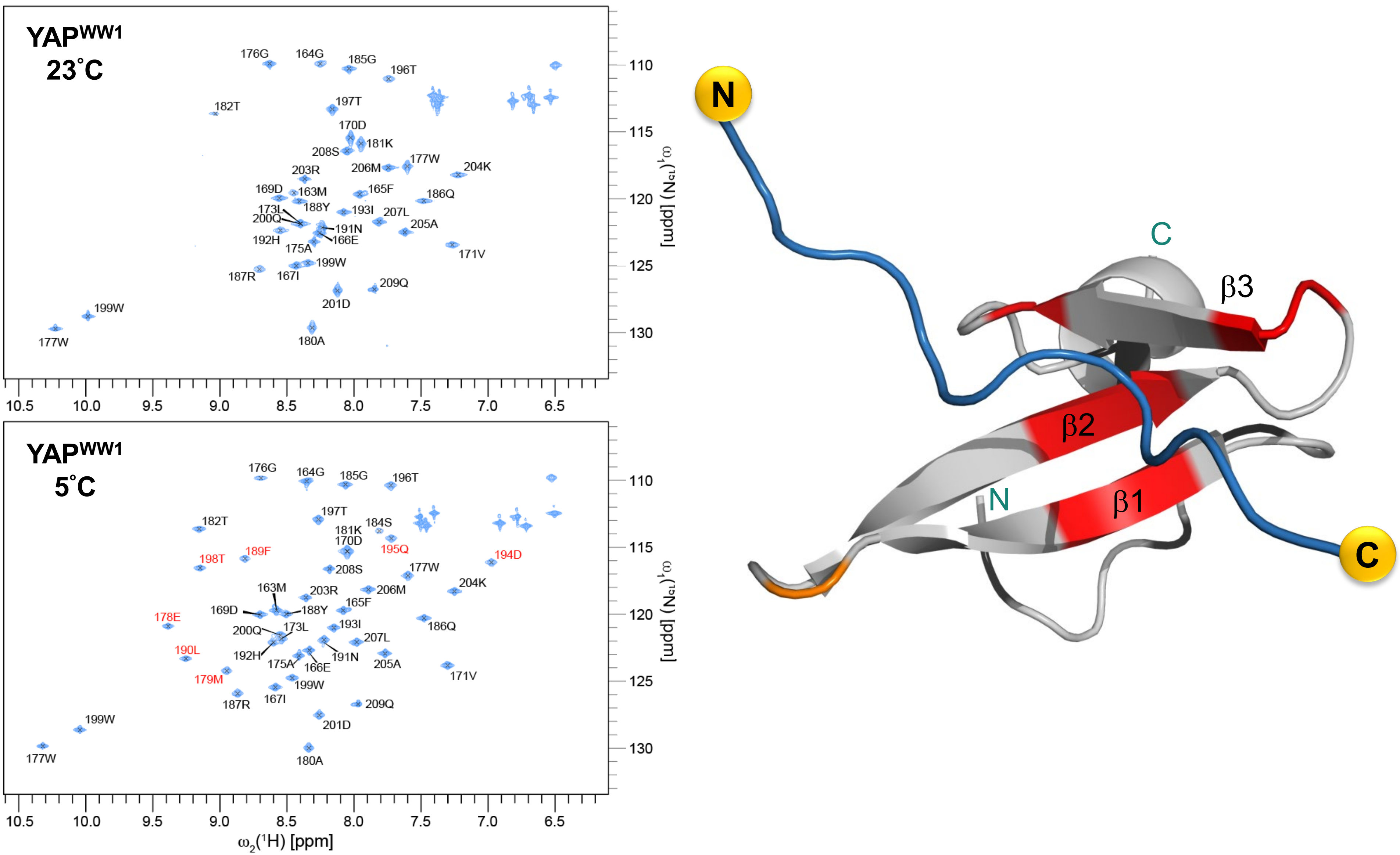

Supplementary Figure S7

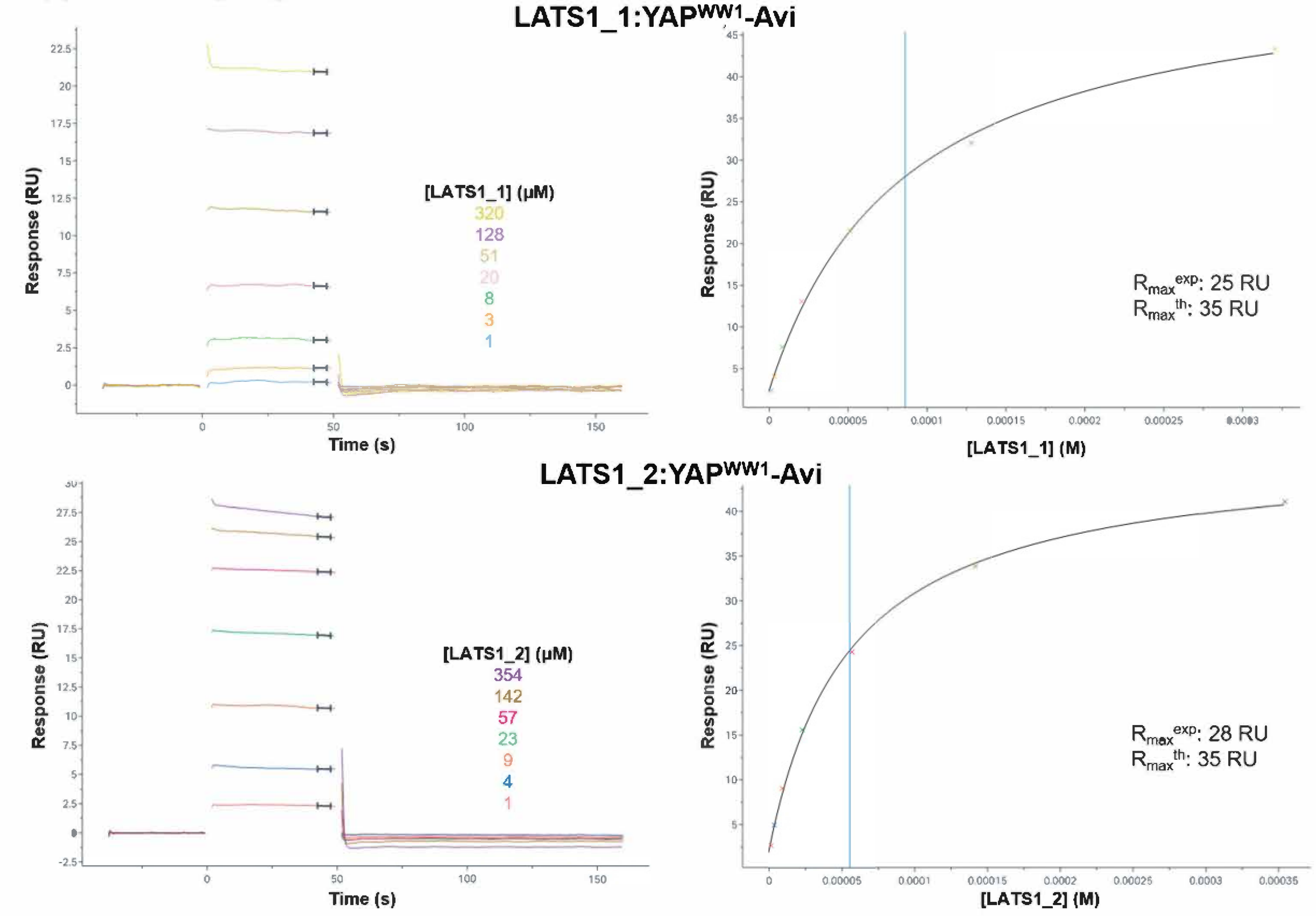

Supplementary Figure S7

mLATS2:YAP^{WW1}-Avi

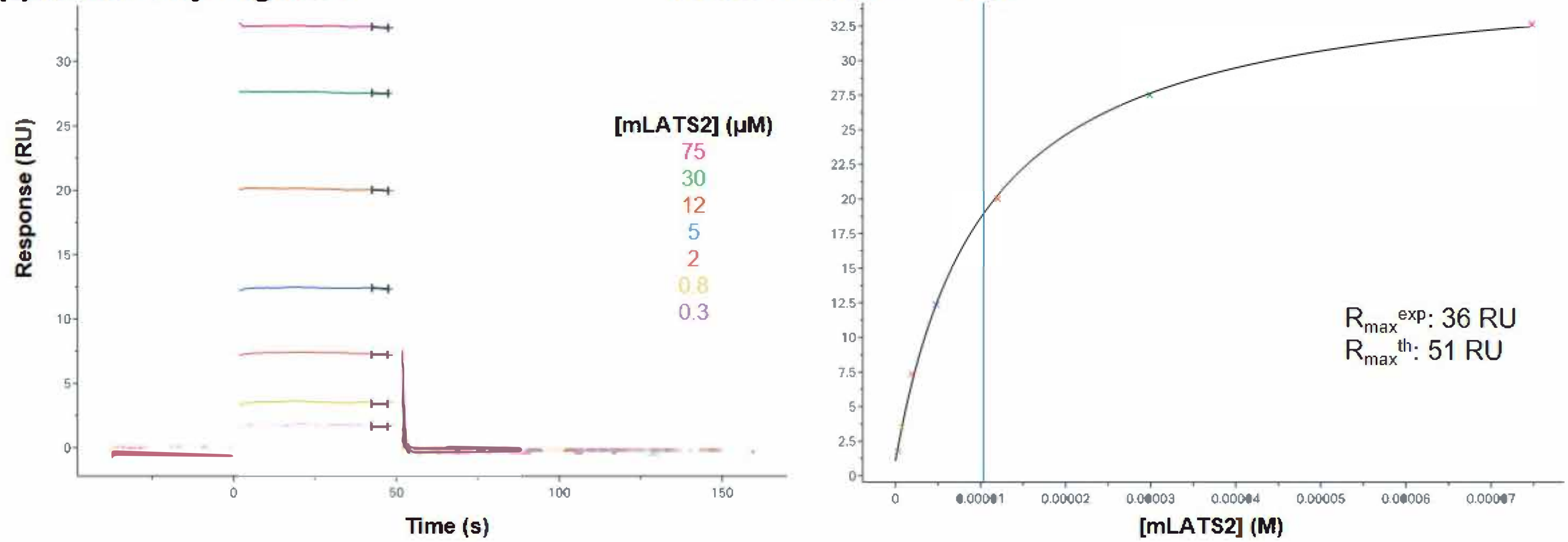

LATS2:YAP^{WW1}-Avi

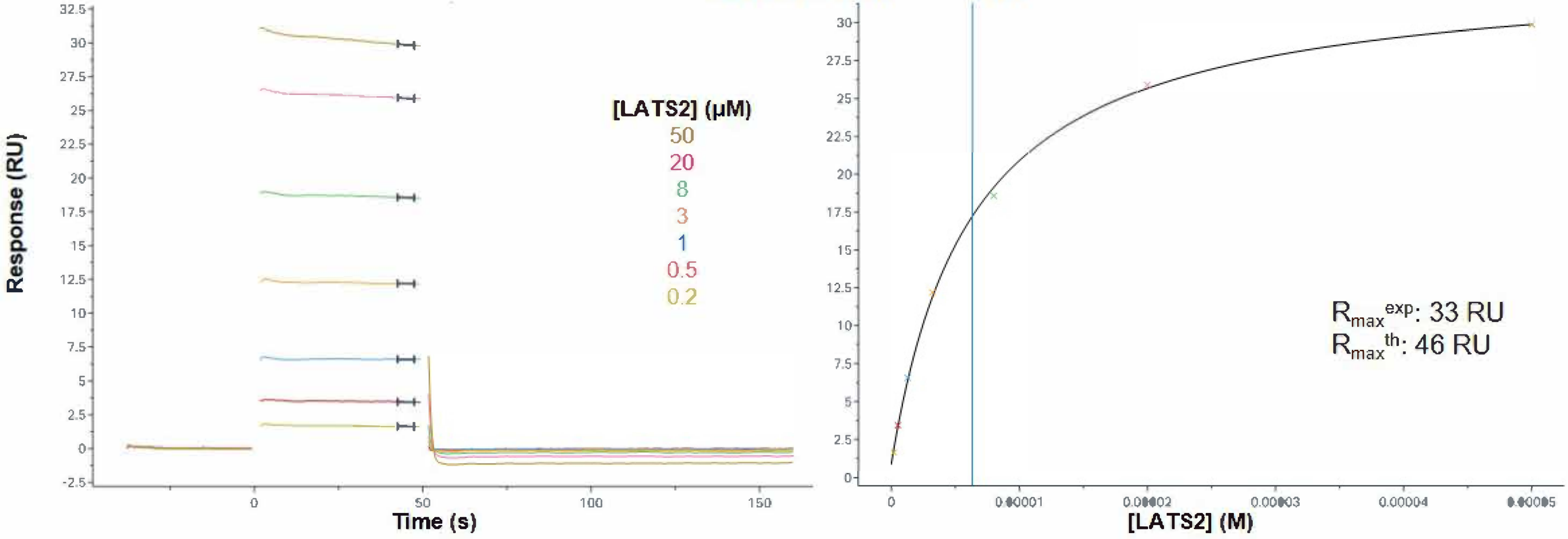

Supplementary Figure S7

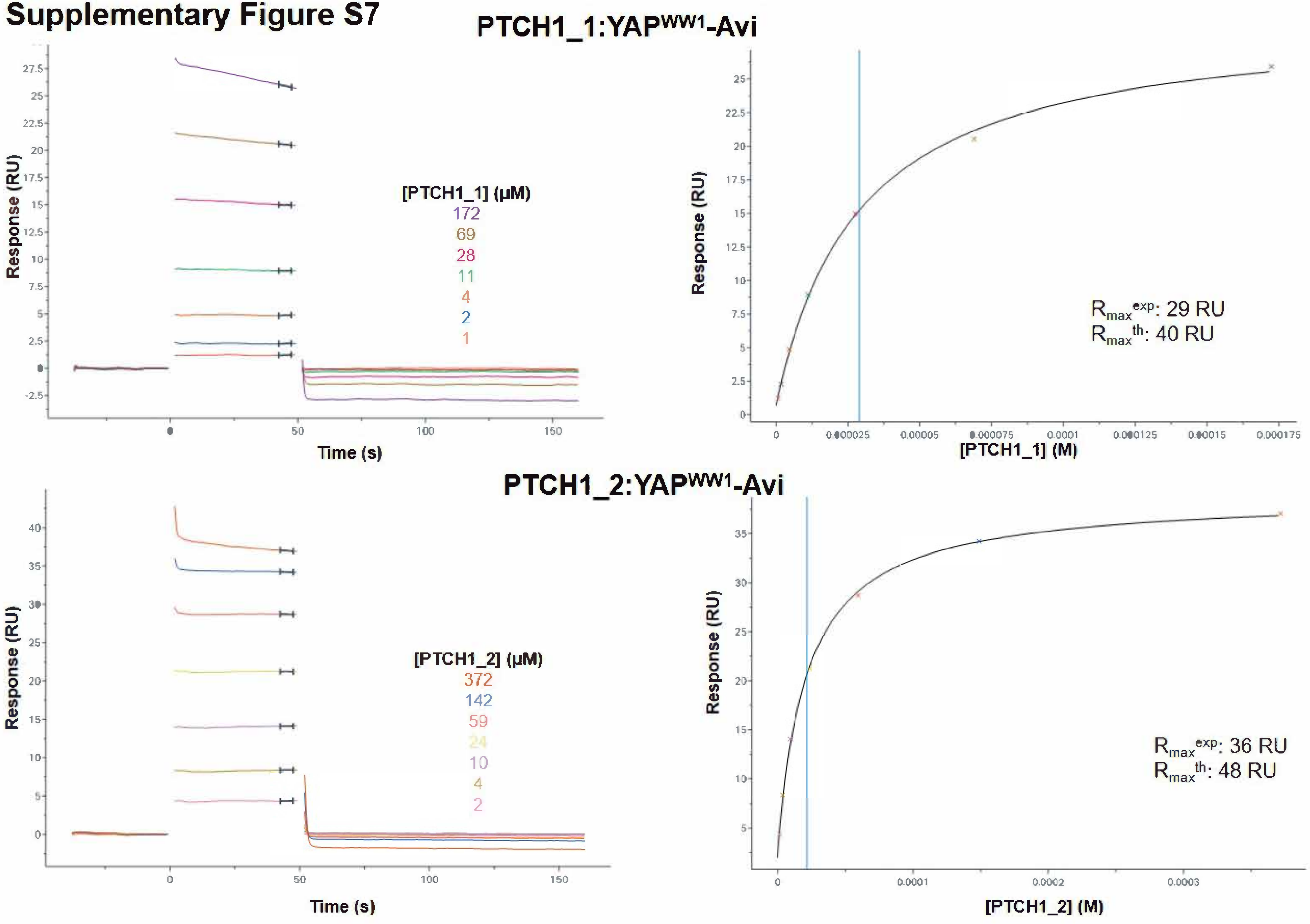

Supplementary Figure S7

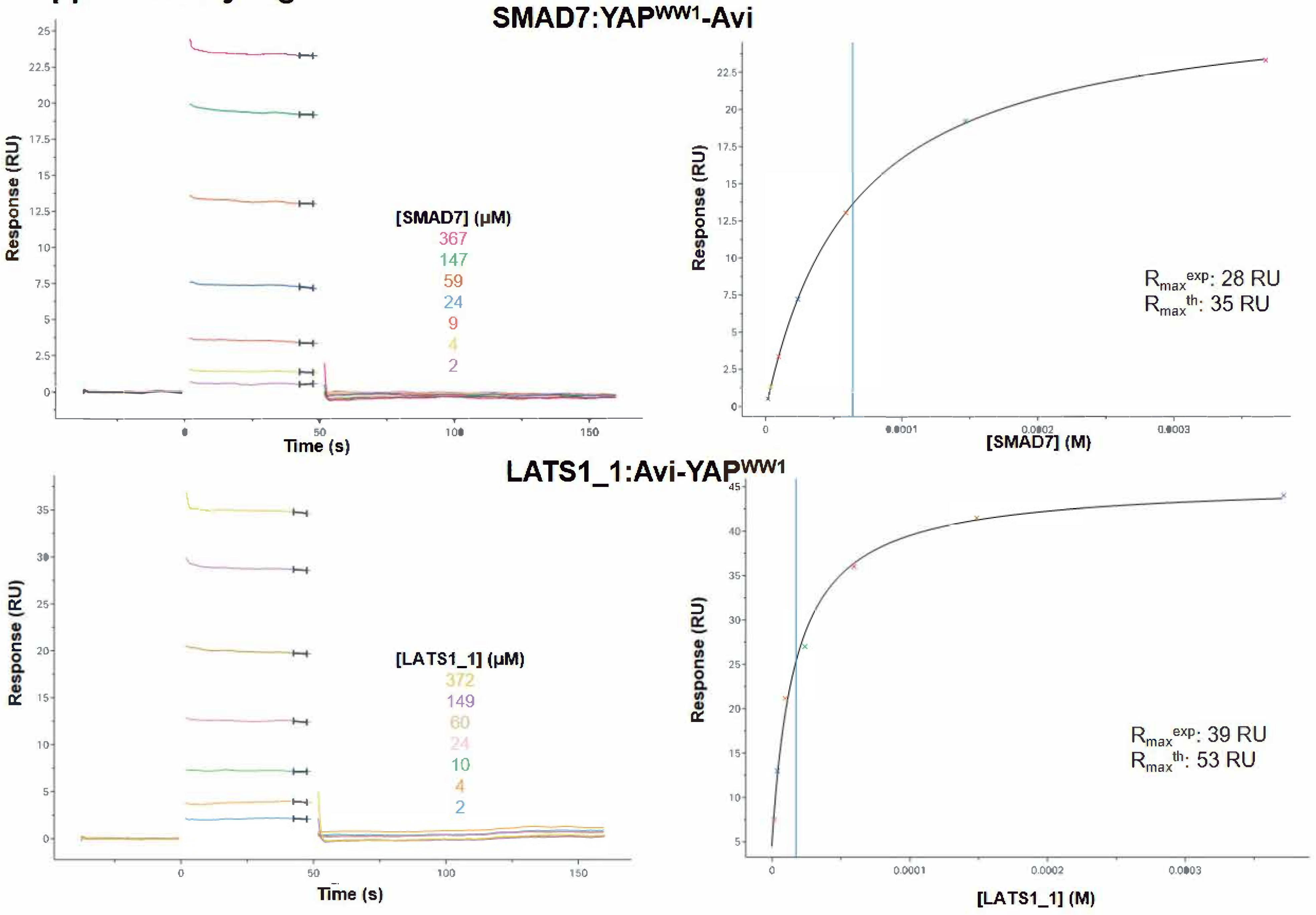

Supplementary Figure S7

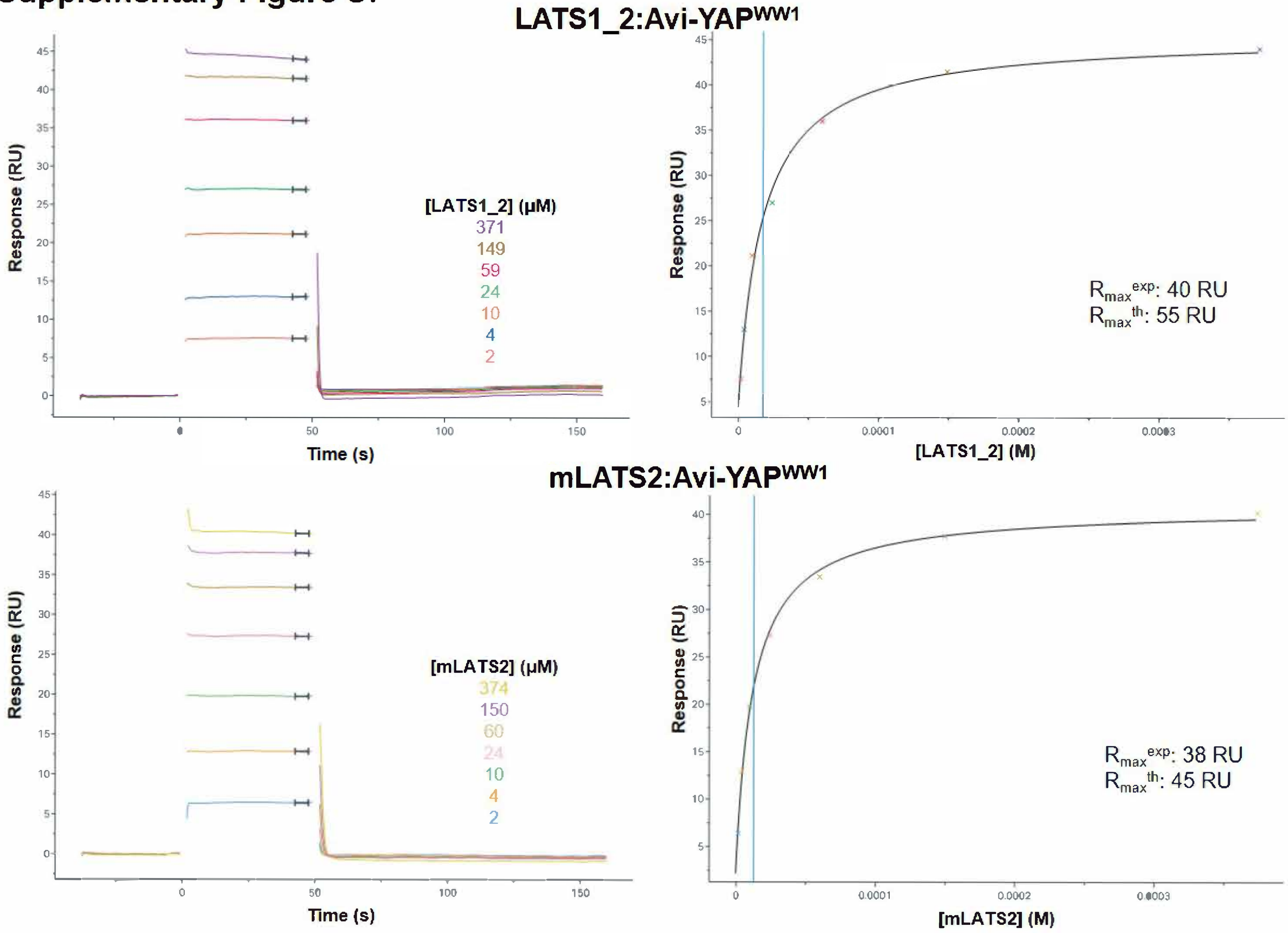

Supplementary Figure S7

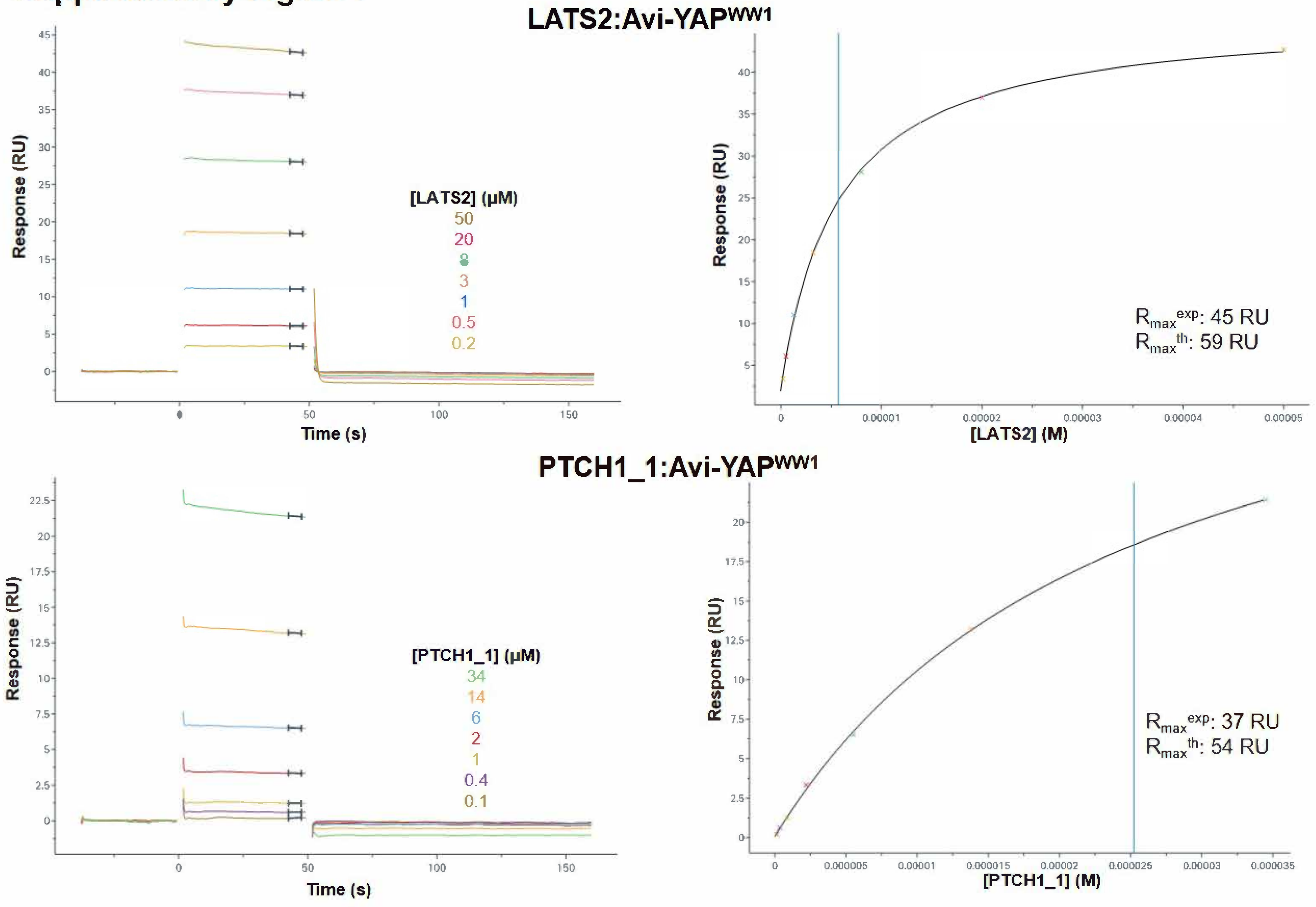

Supplementary Figure S7

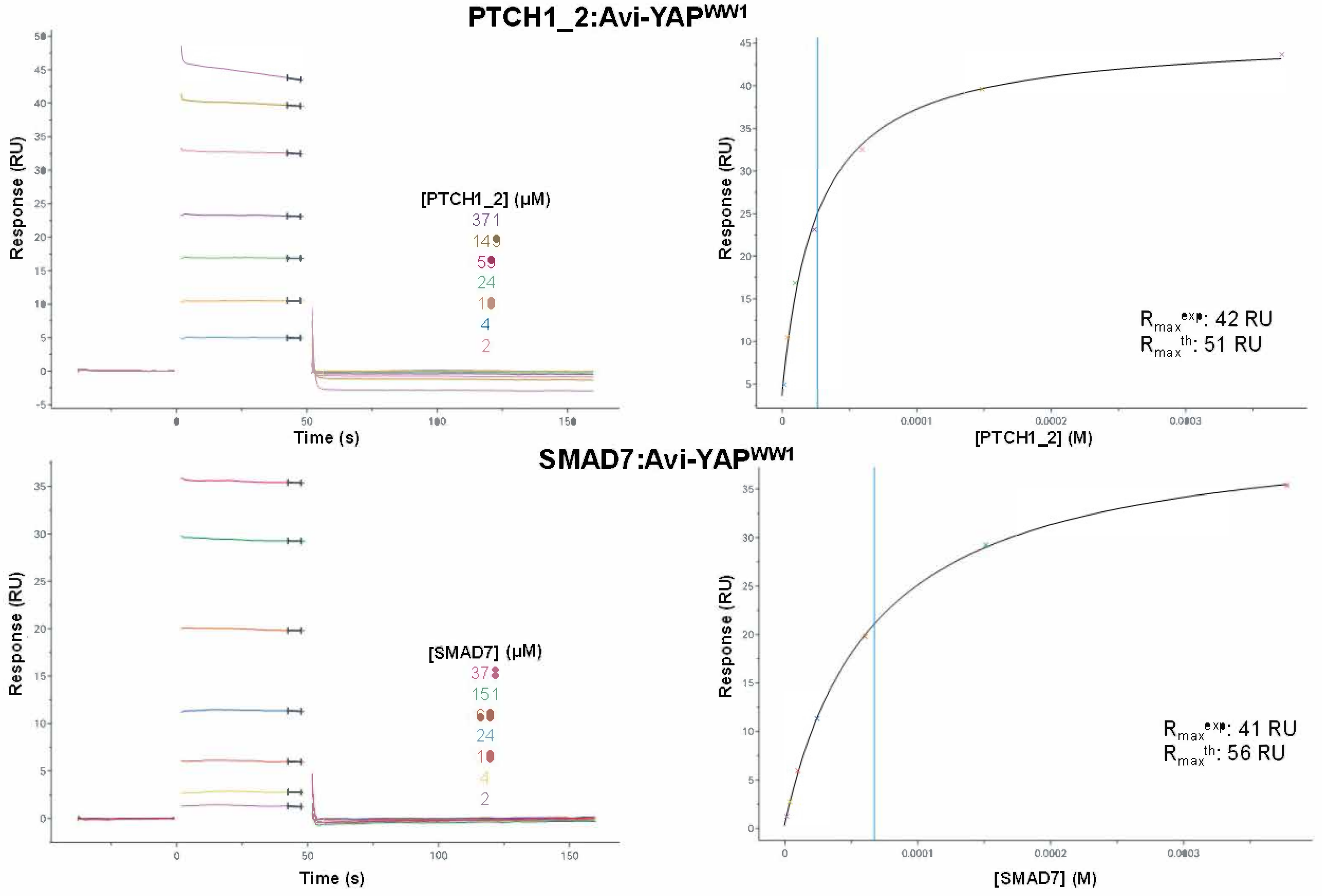

Supplementary Figure S7

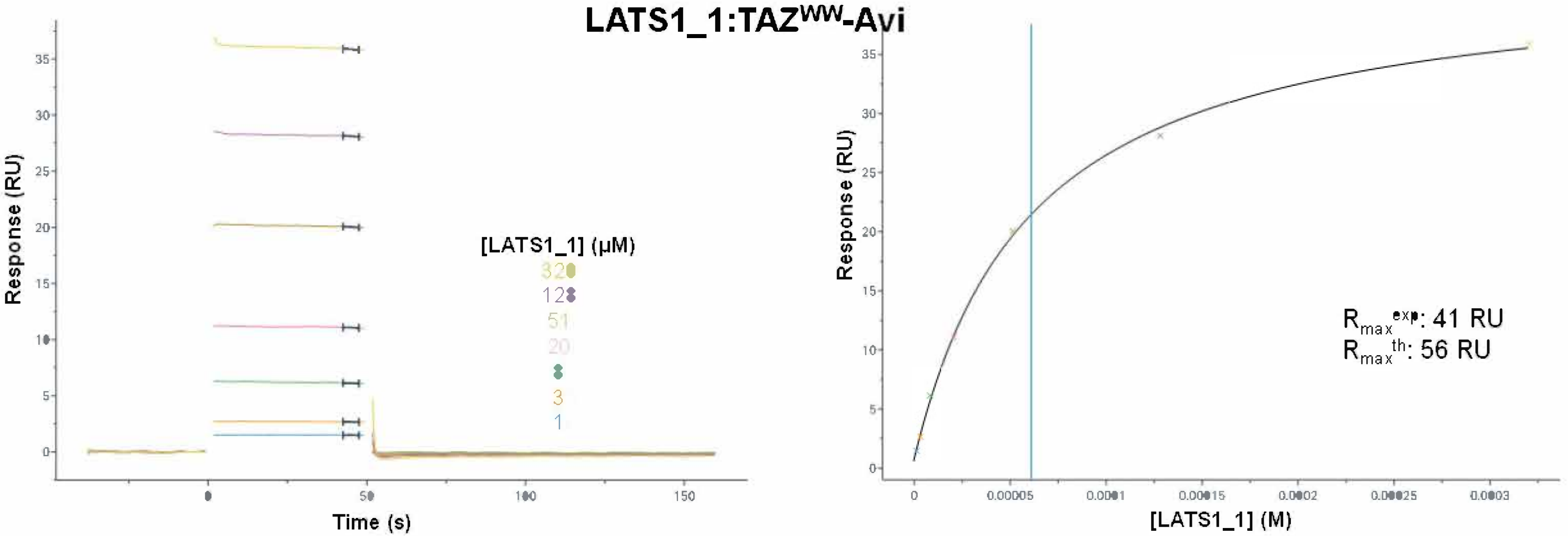

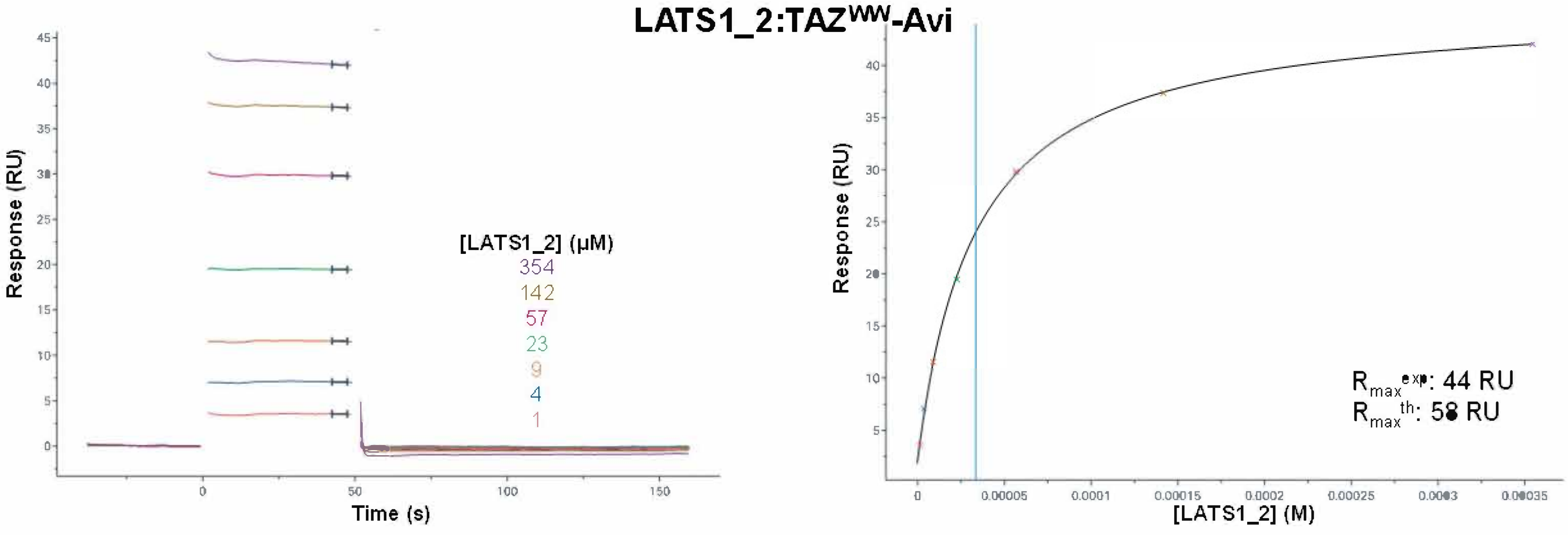

Supplementary Figure S7

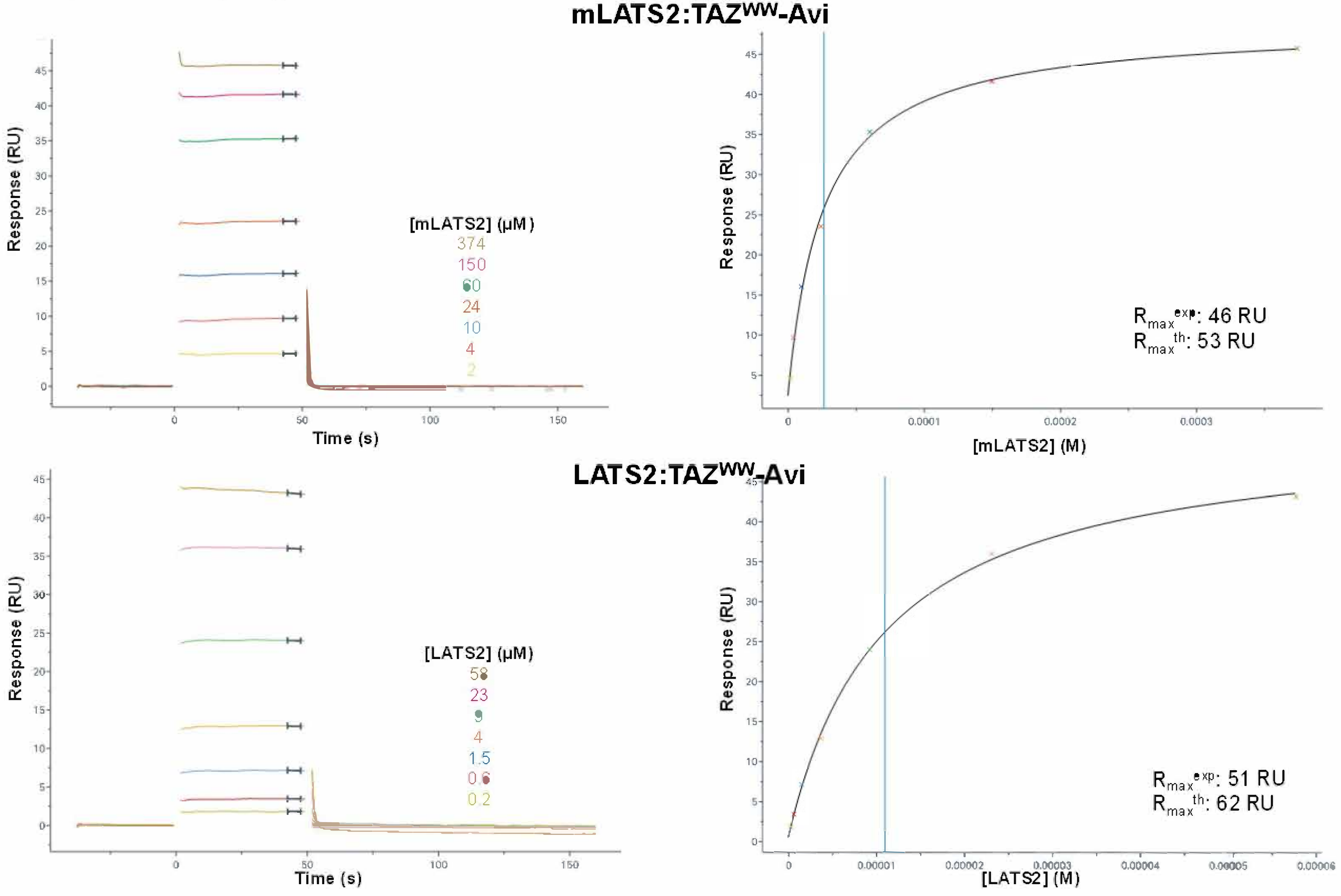

Supplementary Figure S7

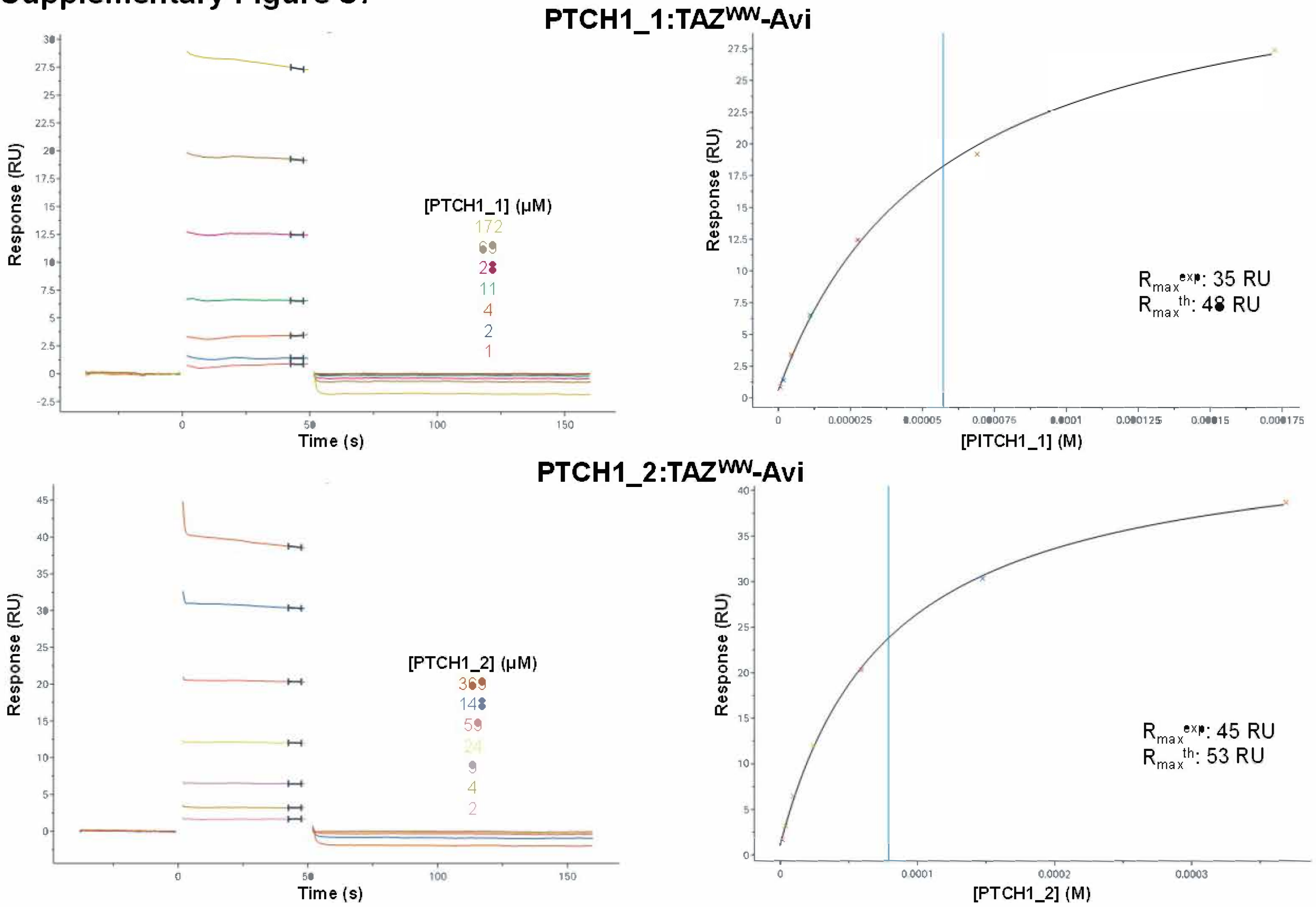

Supplementary Figure S7

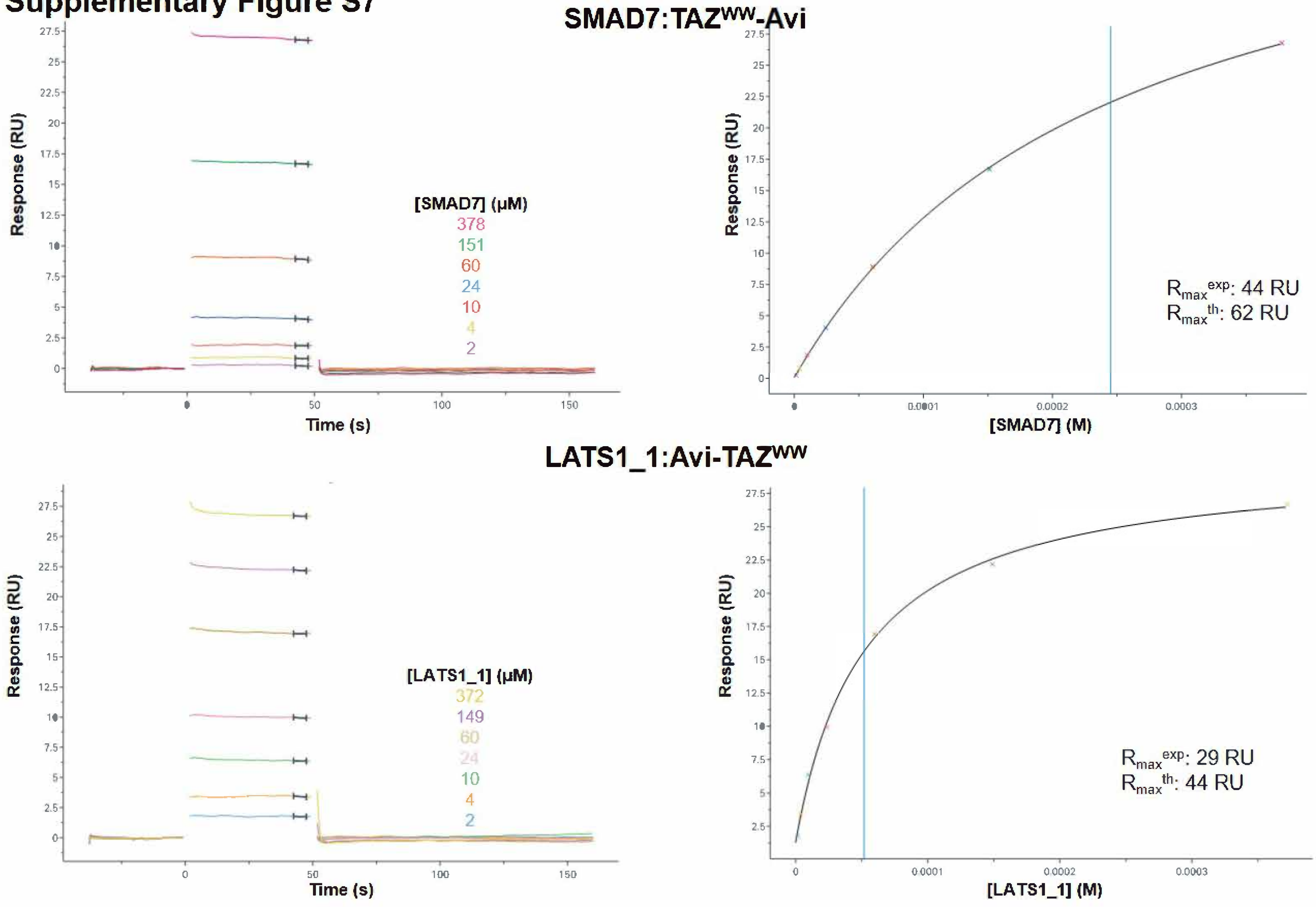

Supplementary Figure S7

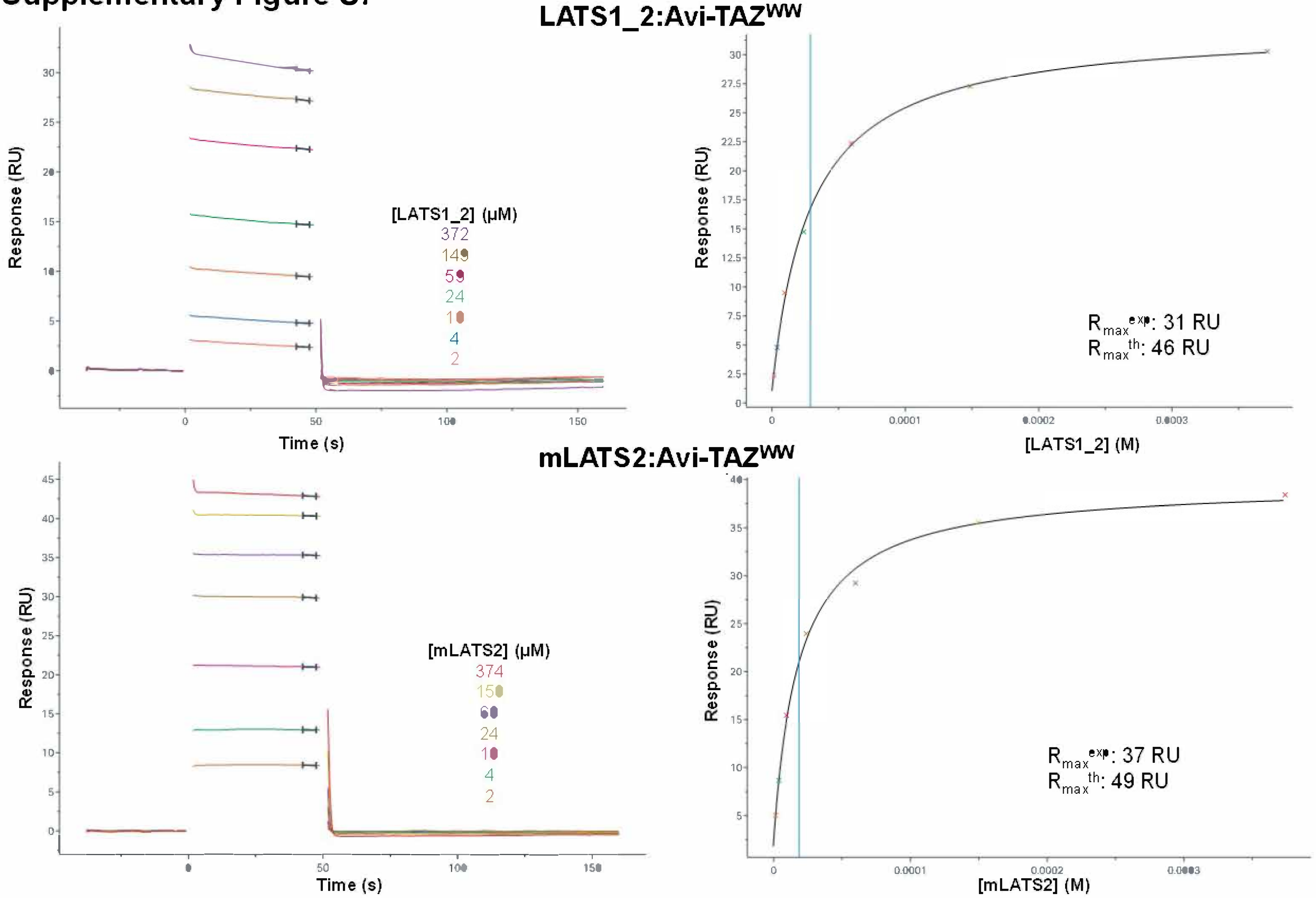

Supplementary Figure S7

LATS2:Avi-TAZ^{WW}

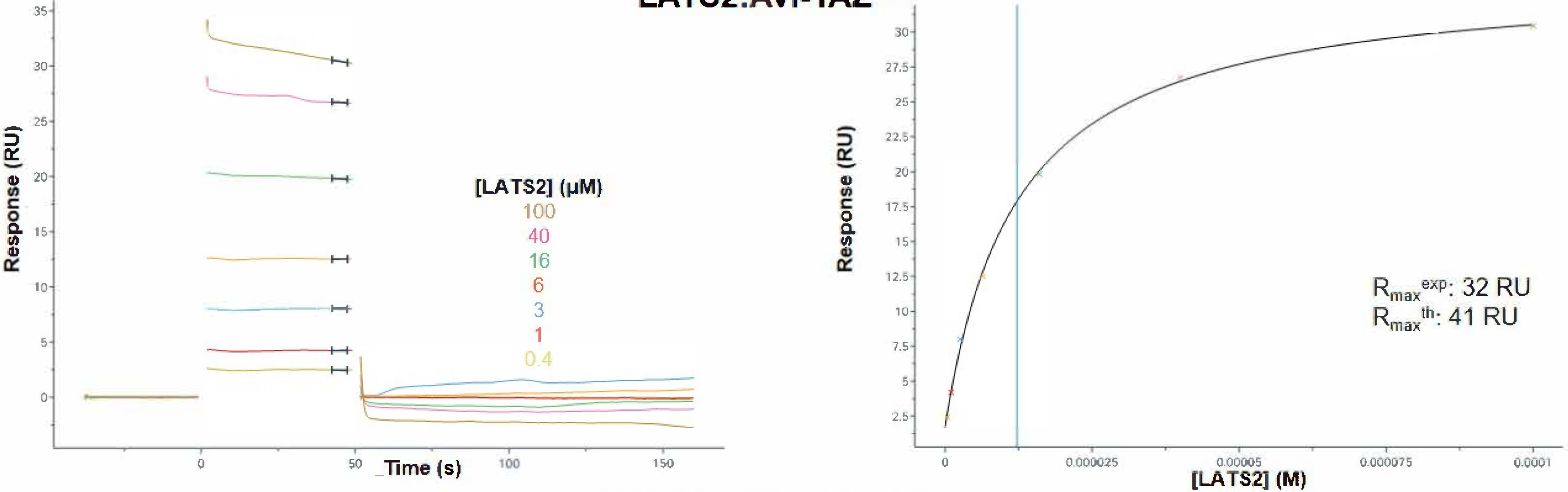

PTCH1_1:Avi-TAZ^{WW}

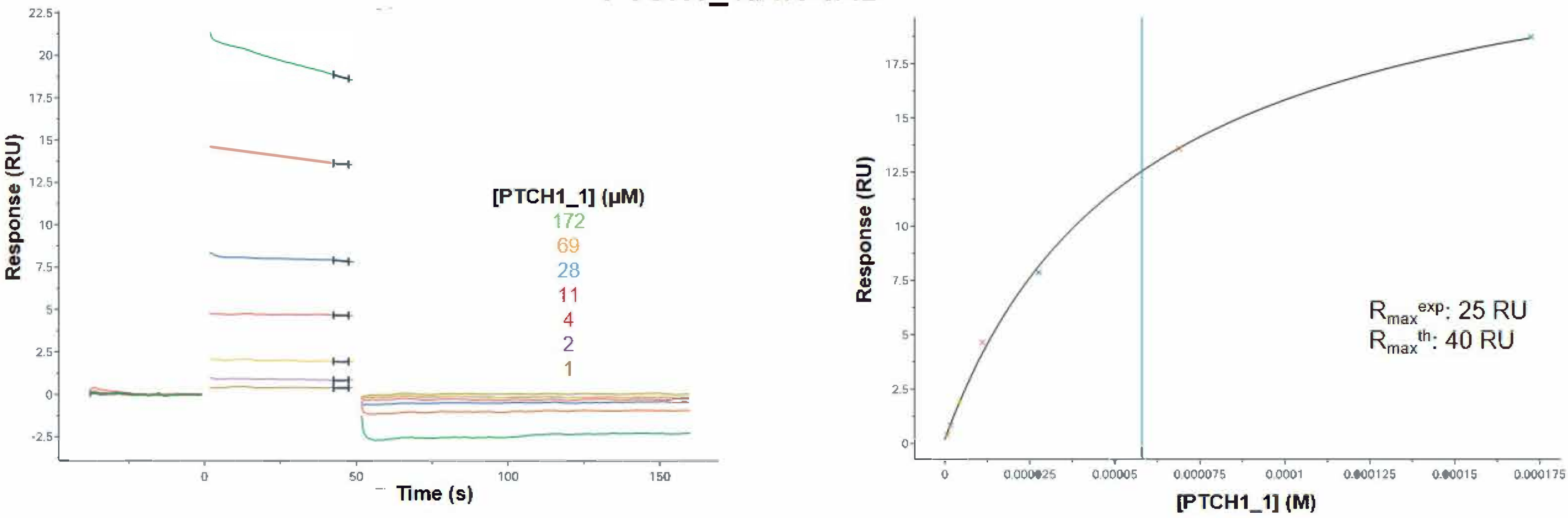

Supplementary Figure S7

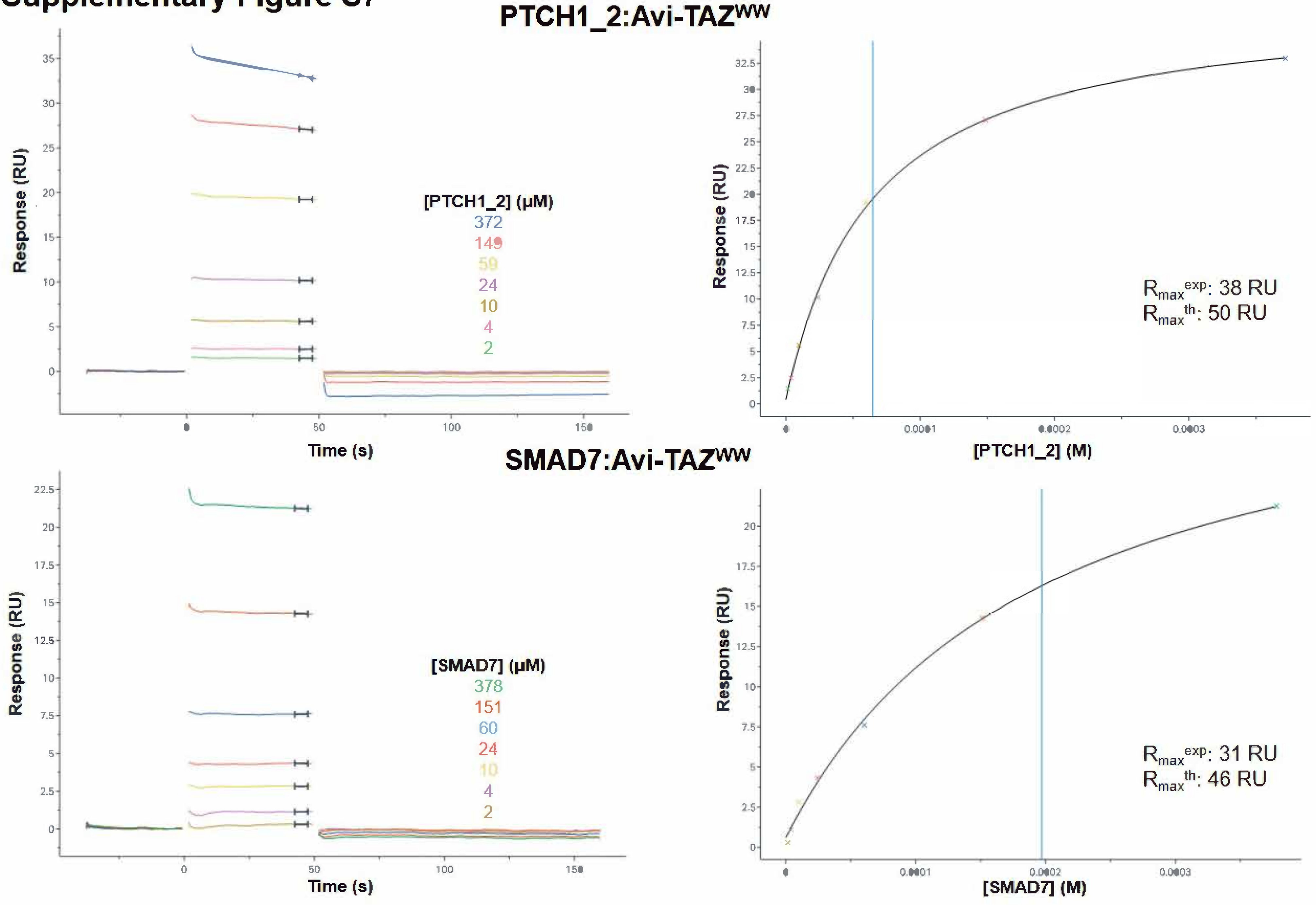

Supplementary Figure S7

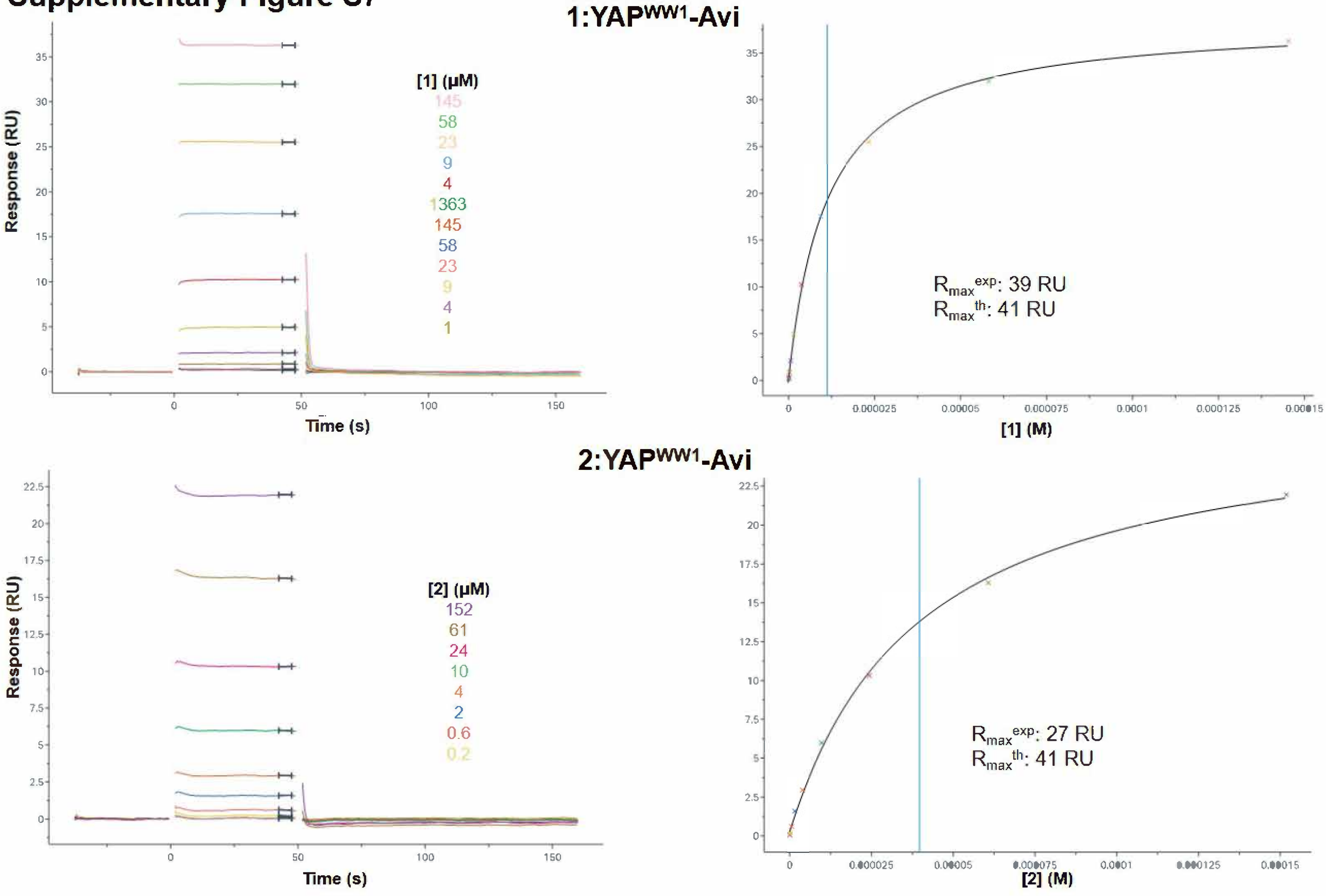

Supplementary Figure S7

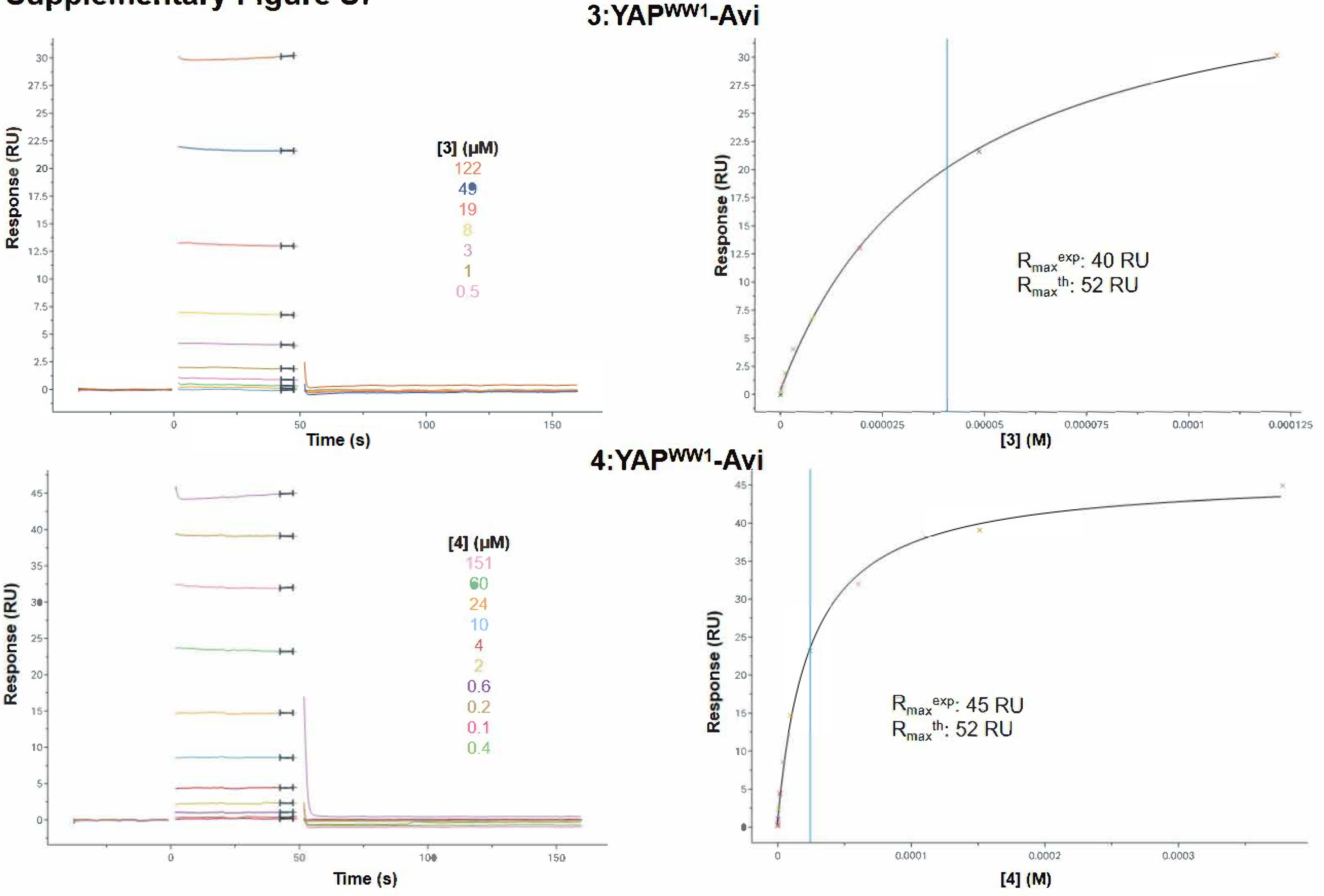

Supplementary Figure S7

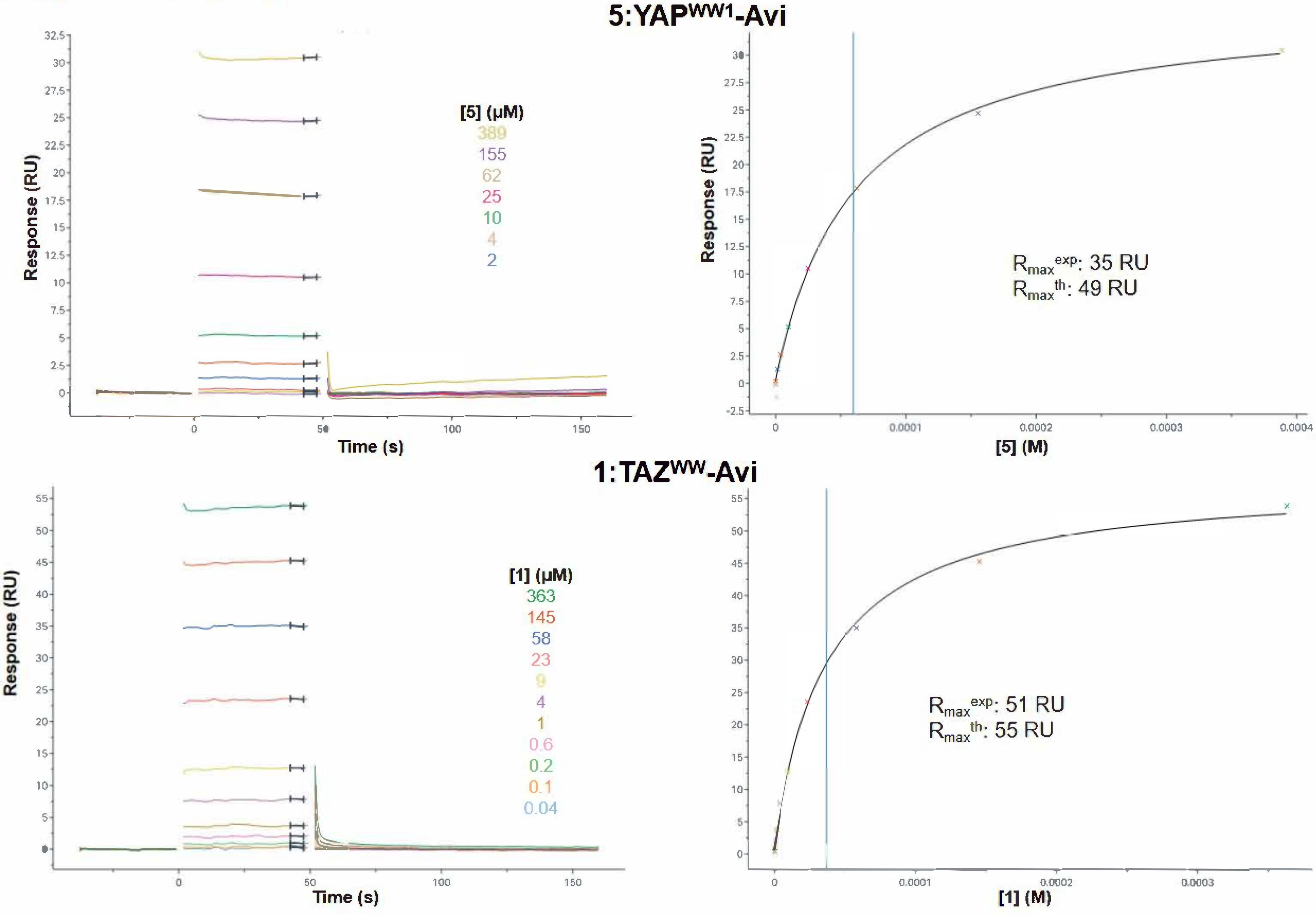

Supplementary Figure S7

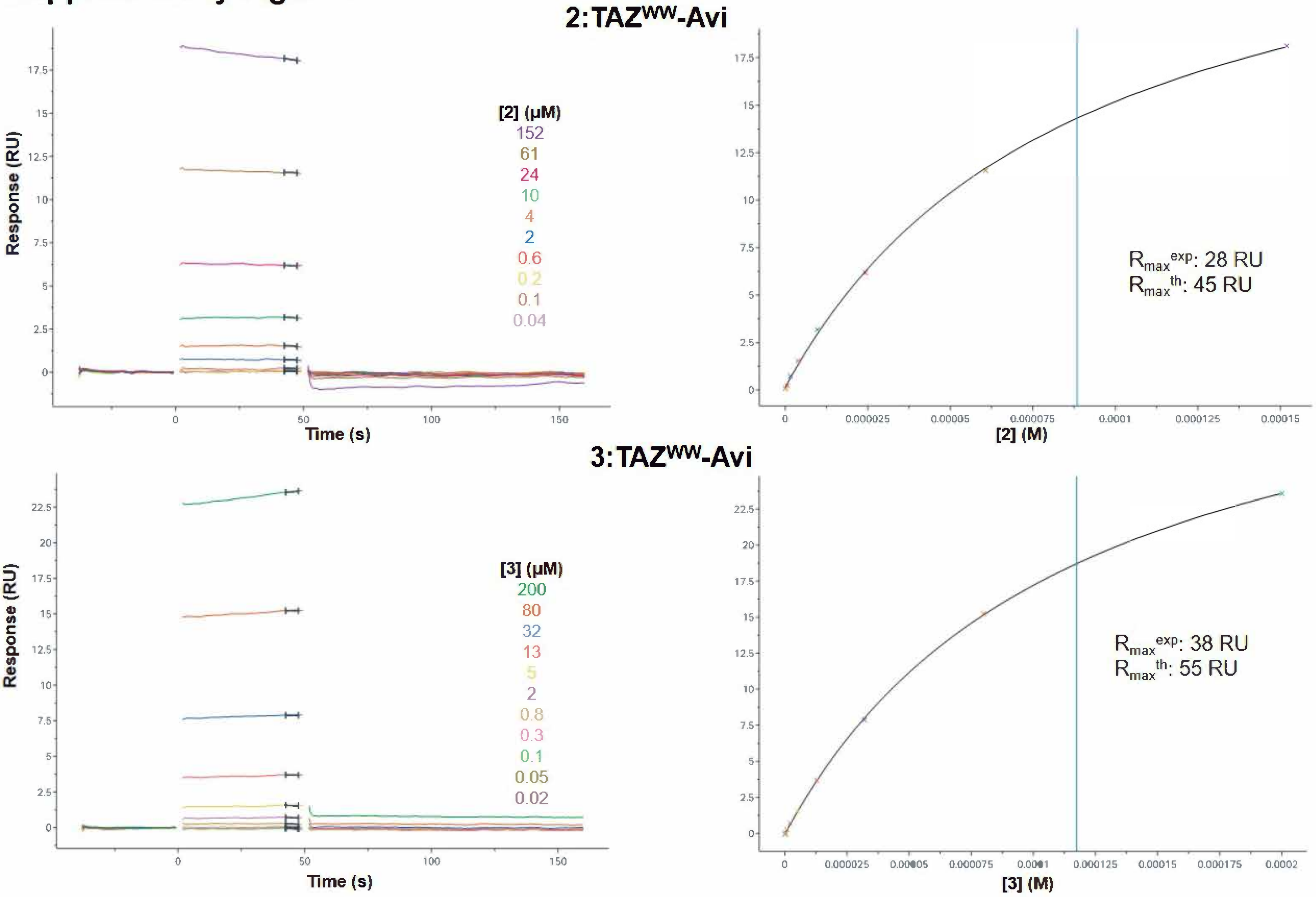

Supplementary Figure S7

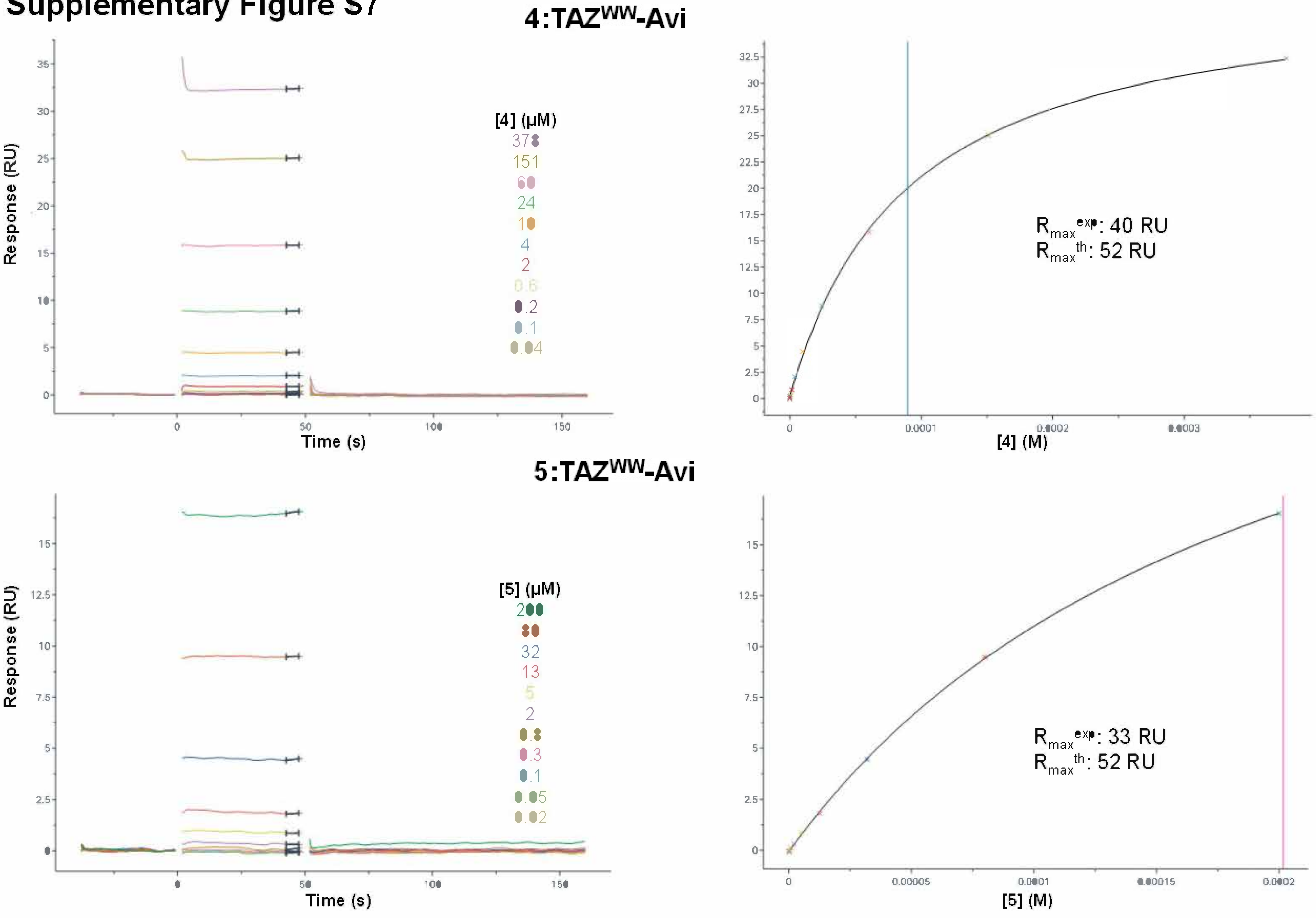

Supplementary Figure S7

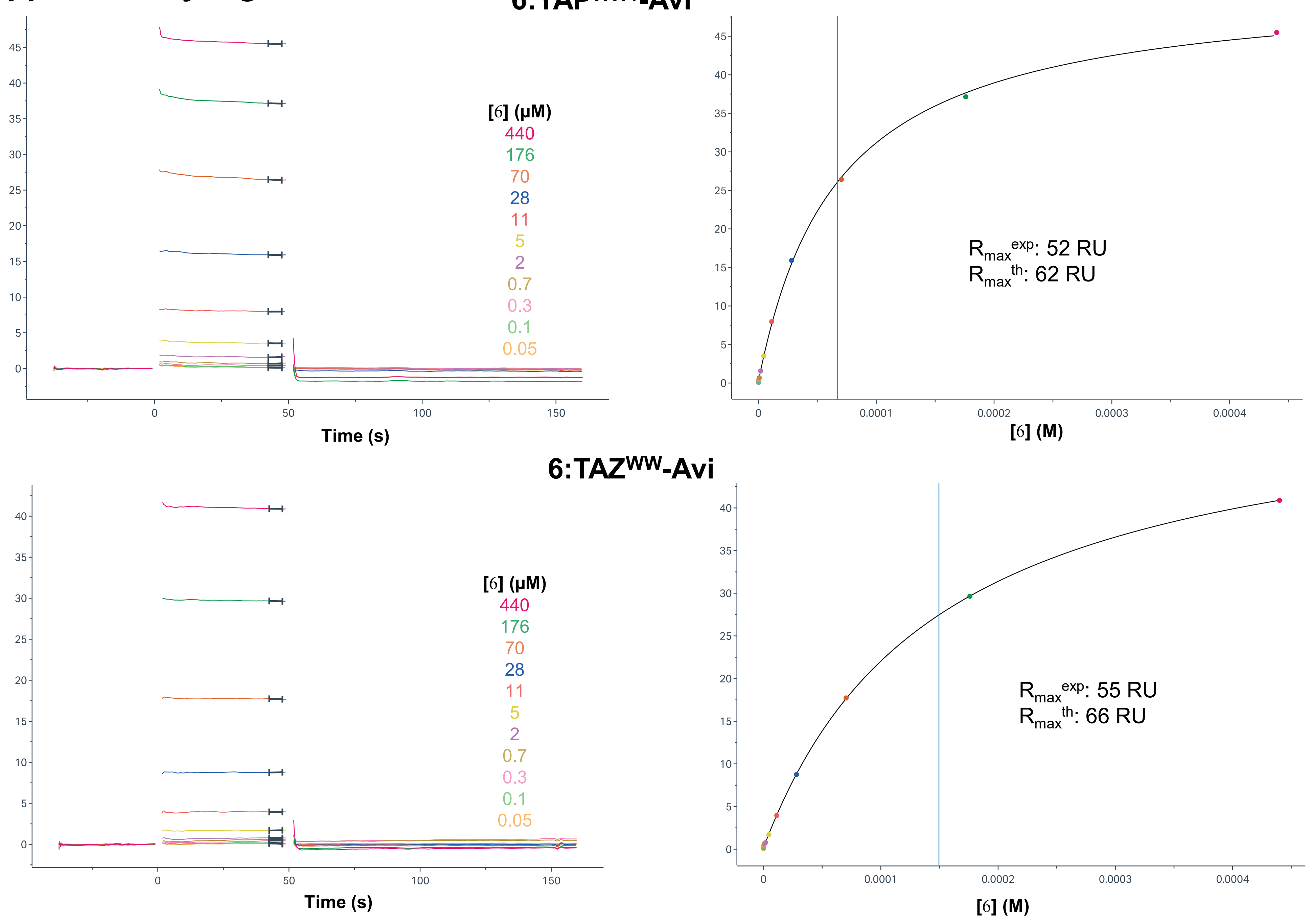

Supplementary Figure S7

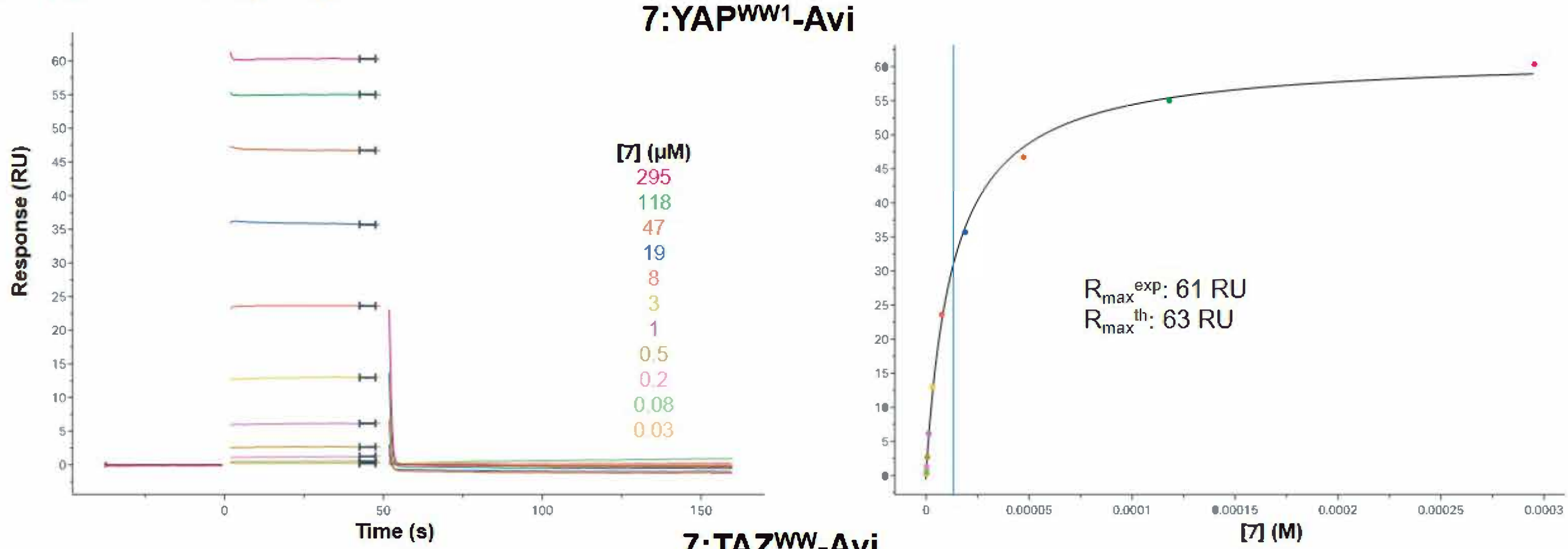

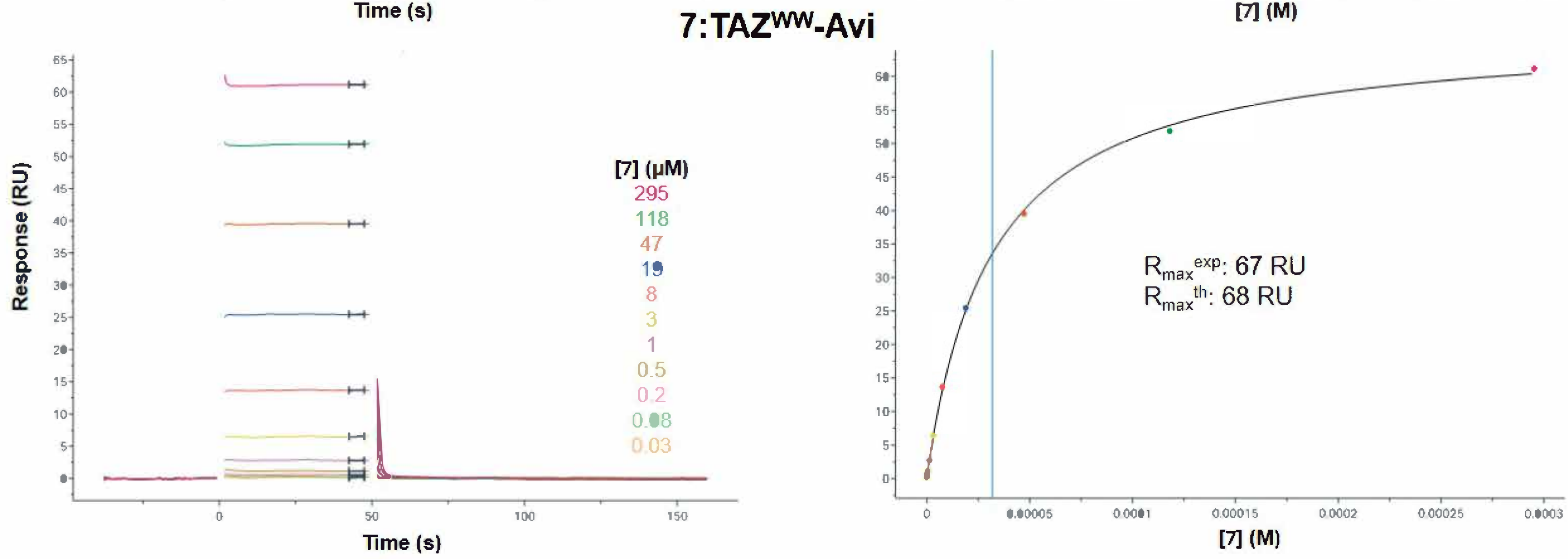

Supplementary Figure S8

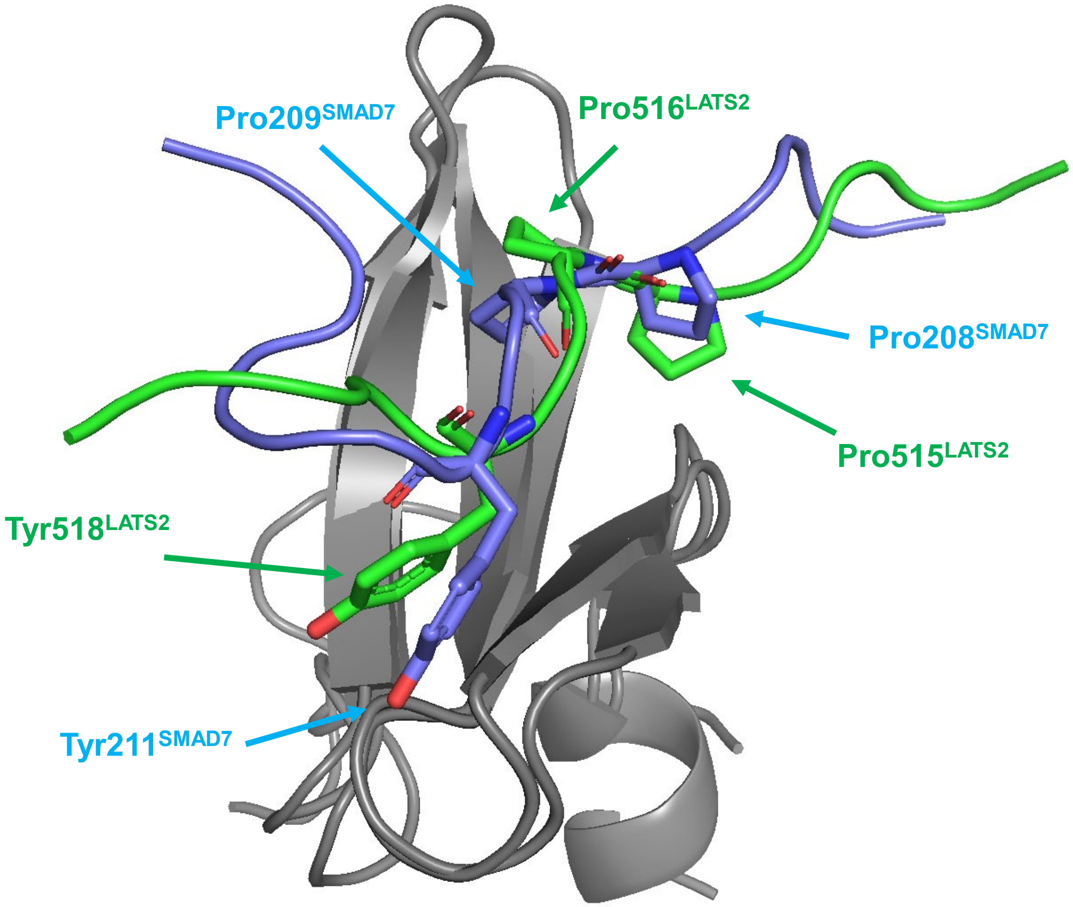

Supplementary Figure S9

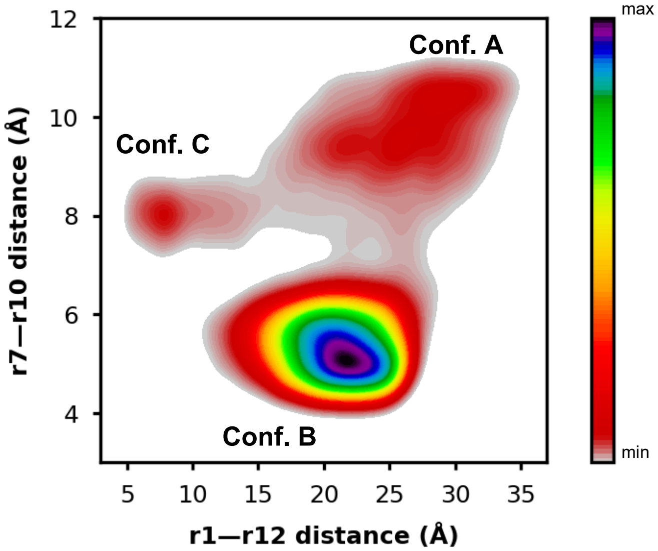

Supplementary Figure S10

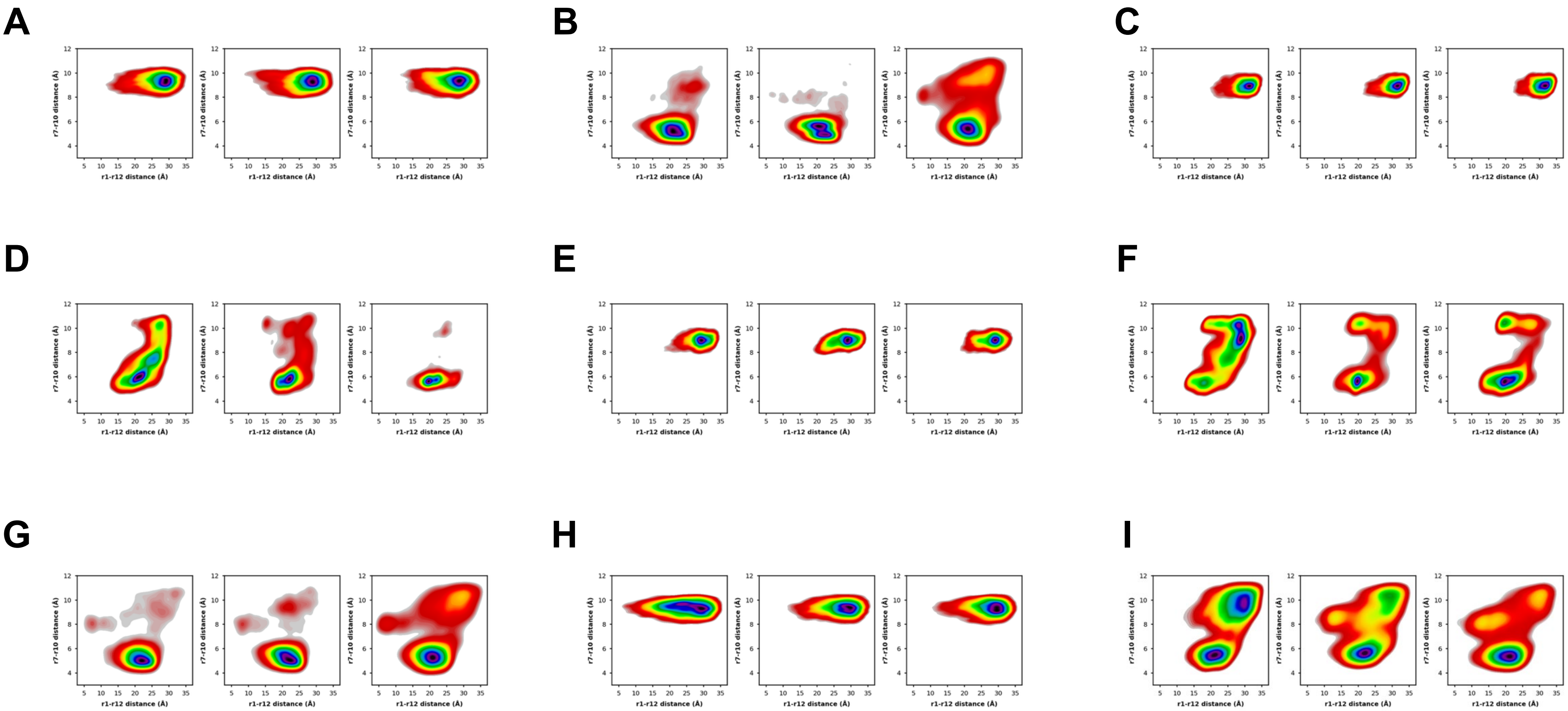

Supplementary Figure S11

LATS1_1 **HPLC-DATA-SHEET**

File: C:\CLASS-VP\PUBLIC\DAD 3\30677.P
Method: C:\CLASS-VP\Methods\ana-reprosil.met
Description: gradient: 5-80% B in 30 min, Temp.55°C
Aquired: 04.12.2023 11:44:47
Printed: 04.12.2023 12:16:41
Column: Reprosil Gold RP18 250 x 3 mm,
Eluent A: 0,05% TFA/Wasser
Eluent B: 0,05% TFA/80% AcN/Wasser
Flow Rate: 0,6 ml/min

Chromatogramm

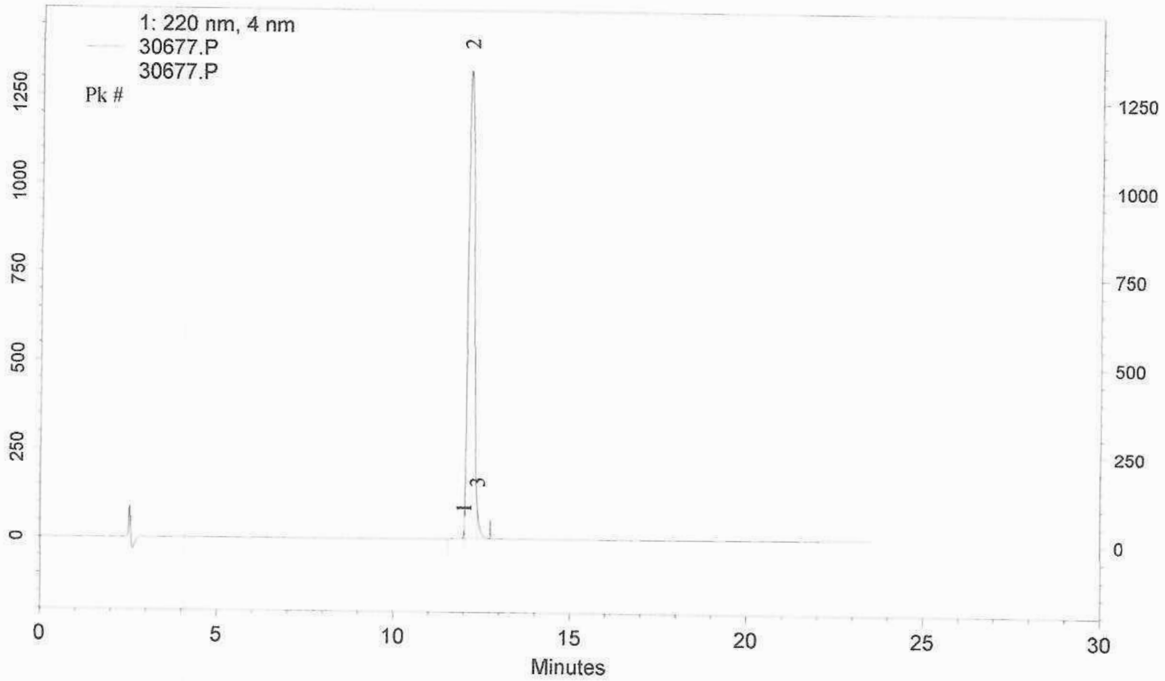

1: 220 nm, 4 nm

Pk #	Retention Time	Area	Area Percent	Name
1	12.0	27145	0.2	
2	12.1	16413587	97.7	
3	12.4	360068	2.1	

Totals				
		16800800	100.0	

TH
CHCA- 30677-P
Data: <Untitled>.E9[c] 4 Dec 2023 11:22 Cal: 25 Apr 2016 11:28
Shimadzu Biotech Axima Assurance 2.9.3.20110624: Mode Linear, Power: 31, Blanked, P.Ext. @ 1391 (bin 57)
%Int. 44 mV[sum= 1399 mV] Profiles 1-32 Smooth Gauss 100 -Baseline 100

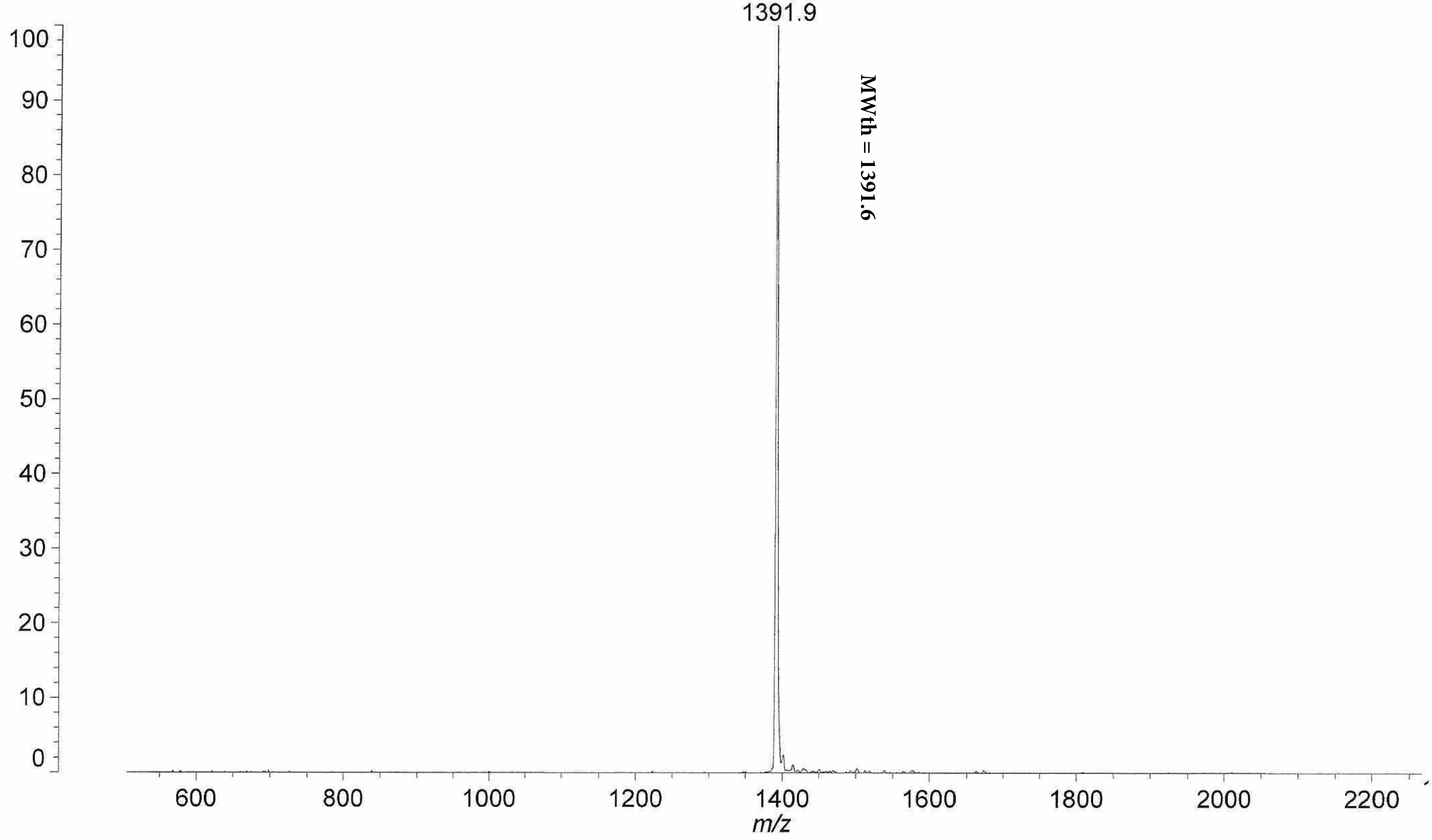

LATS1_2 **HPLC-DATA-SHEET**

File:	C:\CLASS-VP\PUBLIC\DAD 3\30678.P
Method:	C:\CLASS-VP\Methods\ana-reprosil.met
Description:	gradient: 5-80% B in 30 min, Temp.55°C
Aquired:	04.12.2023 14:12:33
Printed:	04.12.2023 14:38:49
Column:	Reprosil Gold RP18 250 x 3 mm,
Eluent A:	0,05% TFA/Wasser
Eluent B:	0,05% TFA/80% AcN/Wasser
Flow Rate:	0,6 ml/min

Chromatogramm

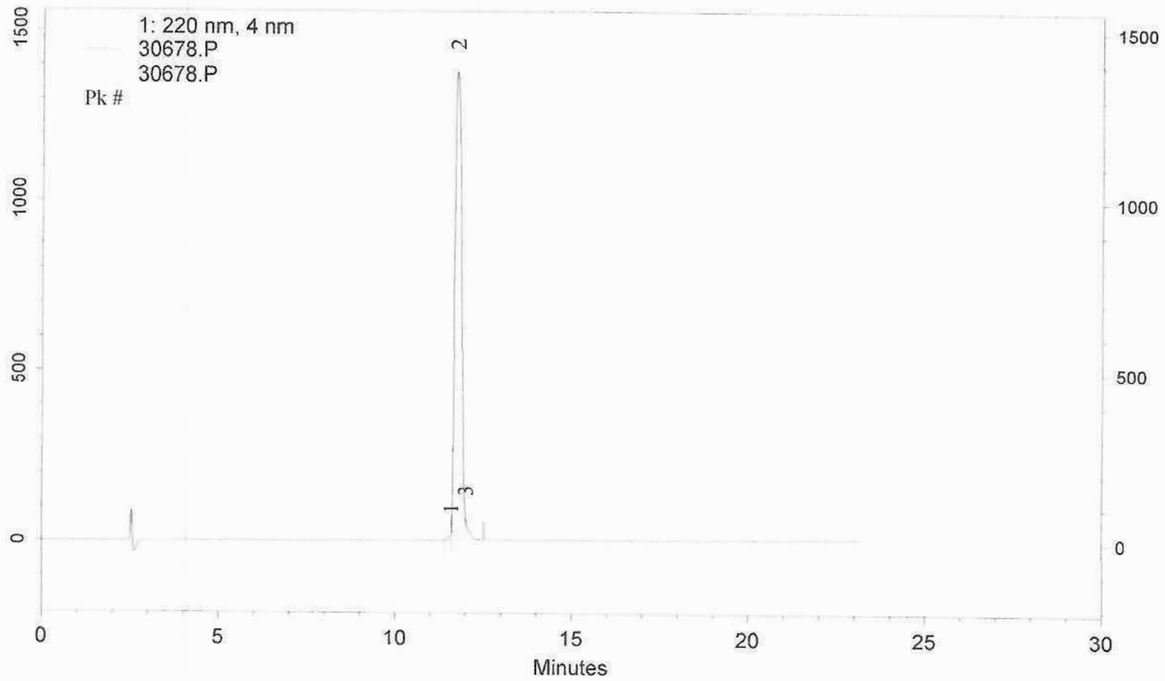

1: 220 nm, 4 nm

Pk #	Retention Time	Area	Area Percent	Name
1	11.6	92516	0.5	
2	11.7	18344628	97.5	
3	12.0	370667	2.0	

Totals		18807811	100.0	

TH
CHCA- 30678-P
Data: <Untitled>.E17[c] 4 Dec 2023 13:54 Cal: 25 Apr 2016 11:28
Shimadzu Biotech Axima Assurance 2.9.3.20110624: Mode Linear, Power: 30, Blanked, P.Ext. @ 1434 (bin 57)
%Int. 28 mV[sum= 1408 mV] Profiles 1-50 Smooth Gauss 100 -Baseline 100

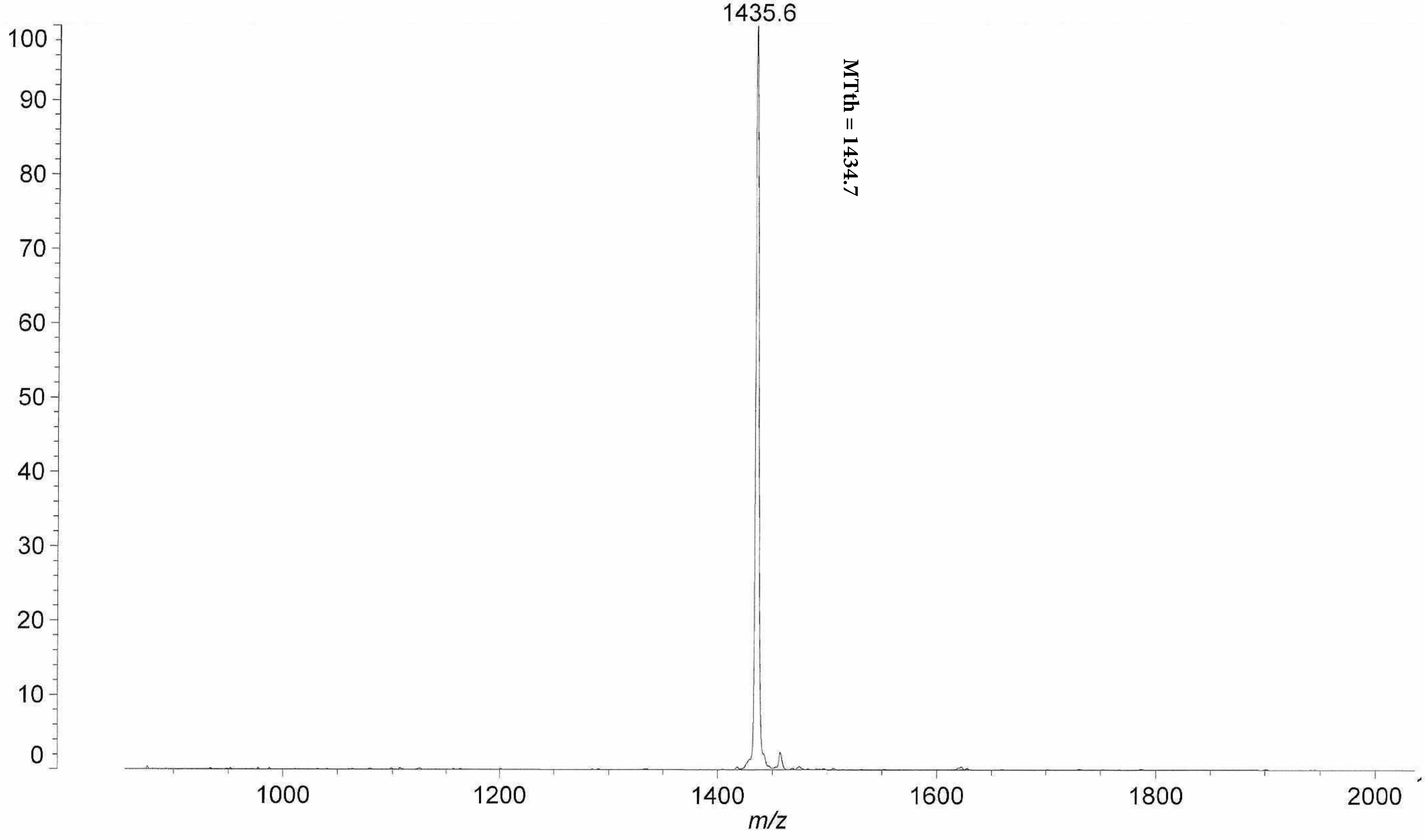

HPLC Data Sheet

LATS2

Sample Name	: 60003-Fraktion
Original Data File	: C:\LabSolutions\Data\PDA-6\60003 -P.lcd
Method File	: Reprosil-C18.lcm
Date Acquired	: 12/01/2026 15:34:44
Date Processed	: 12/01/2026 16:04:28
Comment	: Gradient: 2-77%B in 30min; Temp: 55°C

Column	: Reprosil Gold RP18 250 x 3 mm
Eluent A	: Water + 0.05% TFA
Eluent B	: 80% Acetonitrile + 0.05% TFA
Flow Rate	: 0.6 mL/min

Chromatogram
60003 -P.lcd

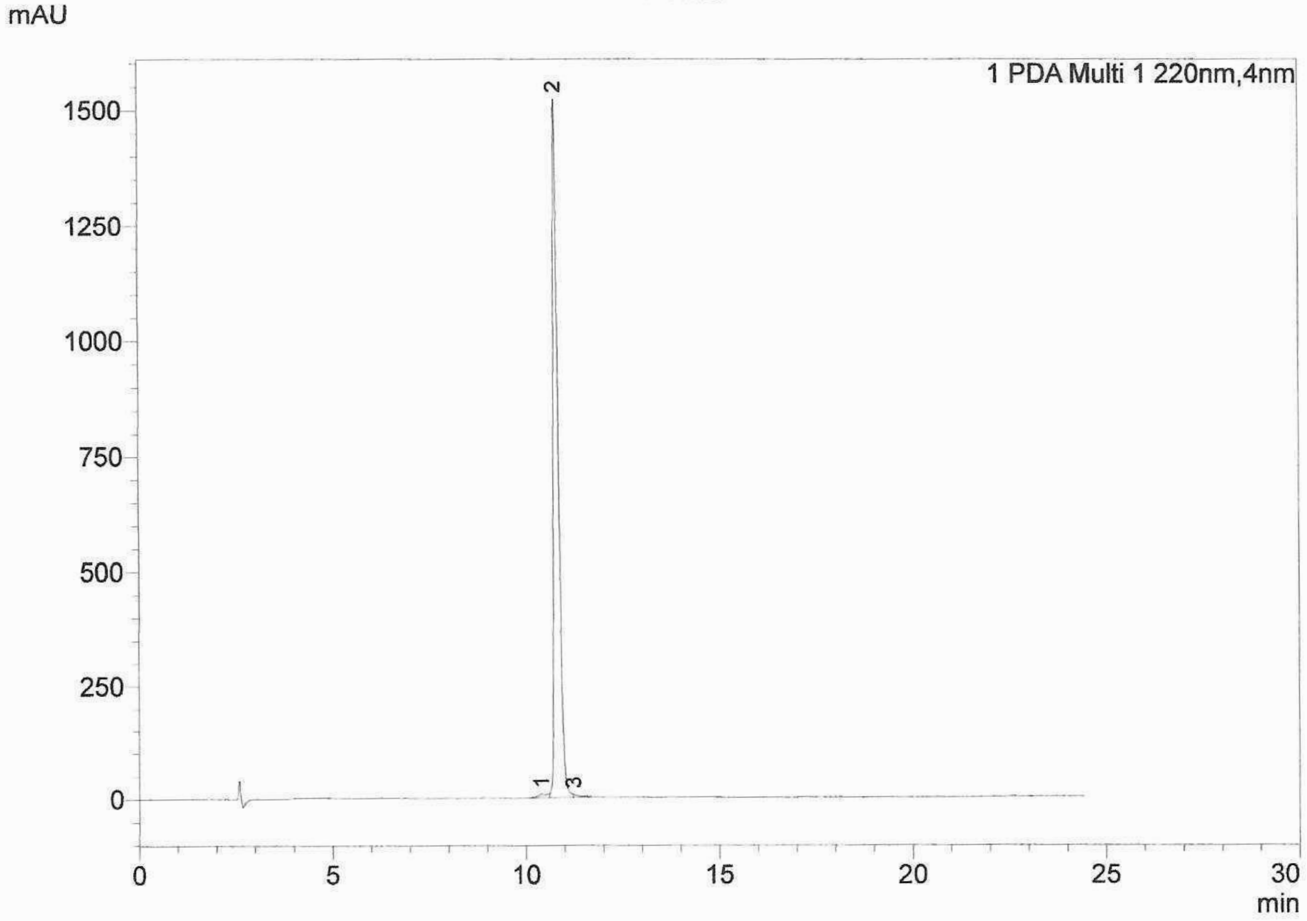

PDA Ch1 220nm

Peak#	Ret. Time	Area	Area%
1	10.398	144443	1.0
2	10.773	13651400	98.5
3	11.225	62417	0.5
Total		13858261	100.0

Created By MALDISolutionsAdmin, Data: 60003-P -CHCA -SK_0001:J3_(Manual) 12 January 2026 15:46:54 Cal:Custom Calibration by MALDISolutionsAdmin on 12 January 2026 15:47:38
Shimadzu MALDI-8030: Tuning Linear, Power 10, P.Ext at 1502 (bin 98), Ion Gate Blanking: 500.00

Processed data (averaged) : 24.8 mV [sum=2407.0 mV], Smoothed = 40, profiles # 1 - 97

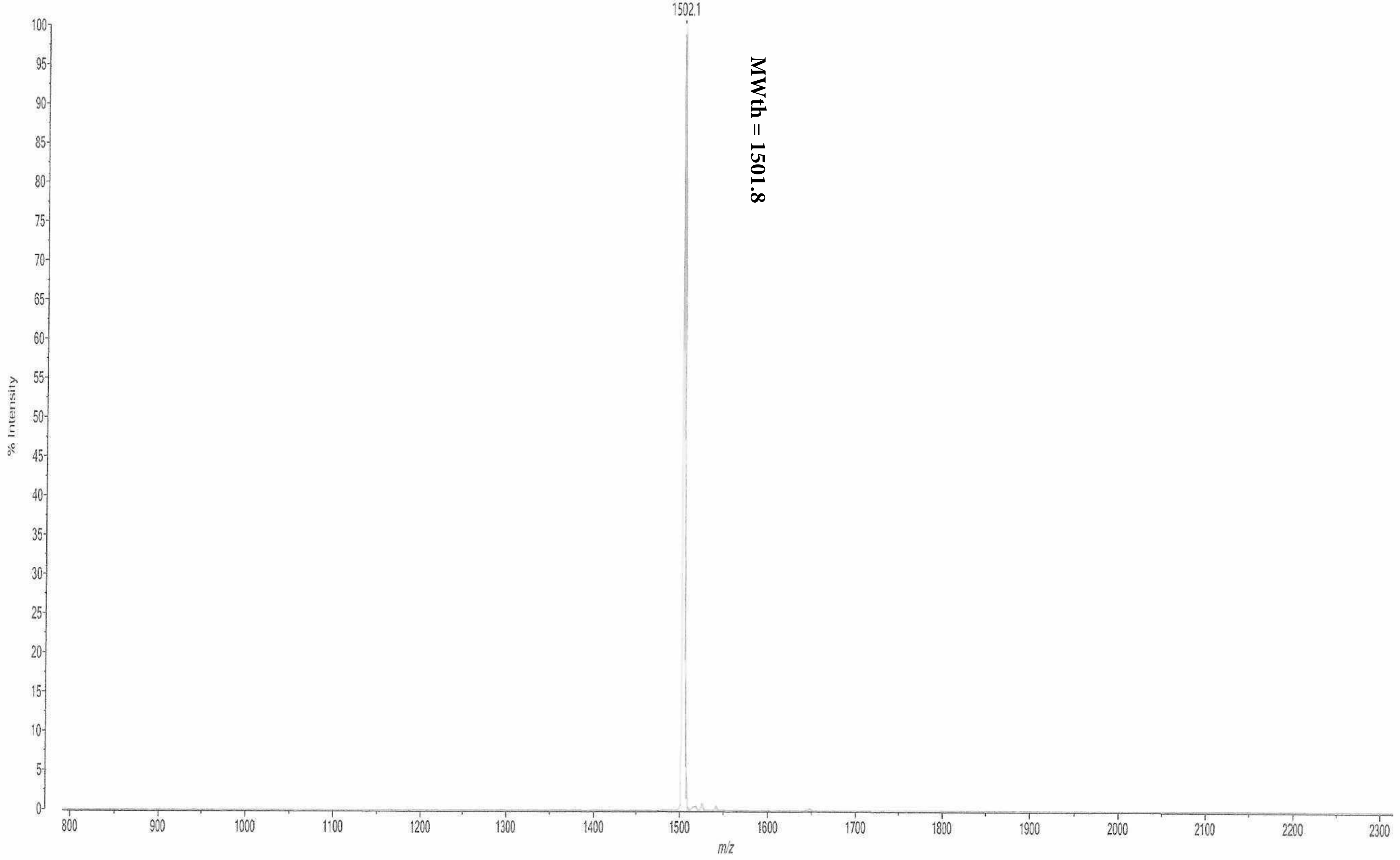

mLATS2 **HPLC-DATA-SHEET**

File:	C:\CLASS-VP\PUBLIC\DAD 3\40010-P
Method:	C:\CLASS-VP\Methods\ana-reprosil.met
Description:	gradient: 5-80% B in 30min, Temp 55°C
Aquired:	24.01.2024 13:07:08
Printed:	24.01.2024 13:29:28
Column:	Reprosil Gold RP18 250 x 3 mm,
Eluent A:	0,05% TFA/Wasser
Eluent B:	0,05% TFA/80% AcN/Wasser
Flow Rate:	0,6 ml/min

Chromatogramm

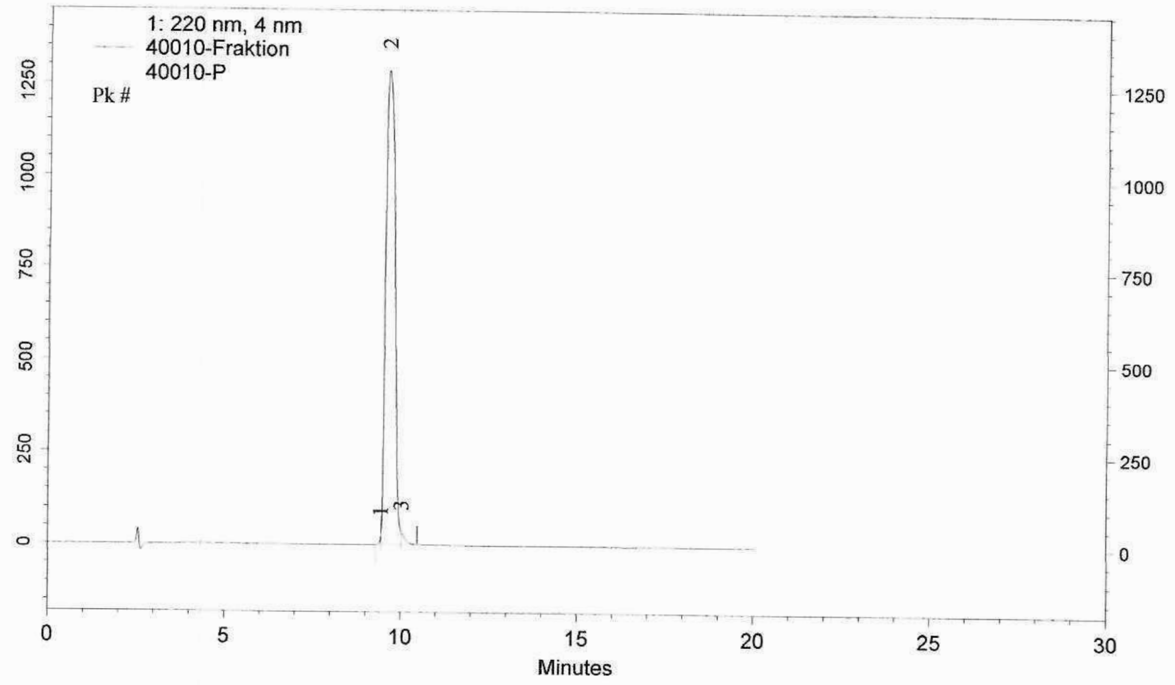

1: 220 nm, 4 nm

Pk #	Retention Time	Area	Area Percent	Name
1	9.4	41616	0.2	
2	9.6	23514935	98.7	
3	10.0	265115	1.1	

Totals				
		23821666	100.0	

TH
CHCA- 40010-P
Data: <Untitled>.K9[c] 25 Jan 2024 12:59 Cal: 25 Apr 2016 11:28
Shimadzu Biotech Axima Assurance 2.9.3.20110624: Mode Linear, Power: 32, Blanked, P.Ext. @ 1485 (bin 58)
%Int. 65 mV[sum= 10620 mV] Profiles 1-163 Smooth Gauss 100 -Baseline 100

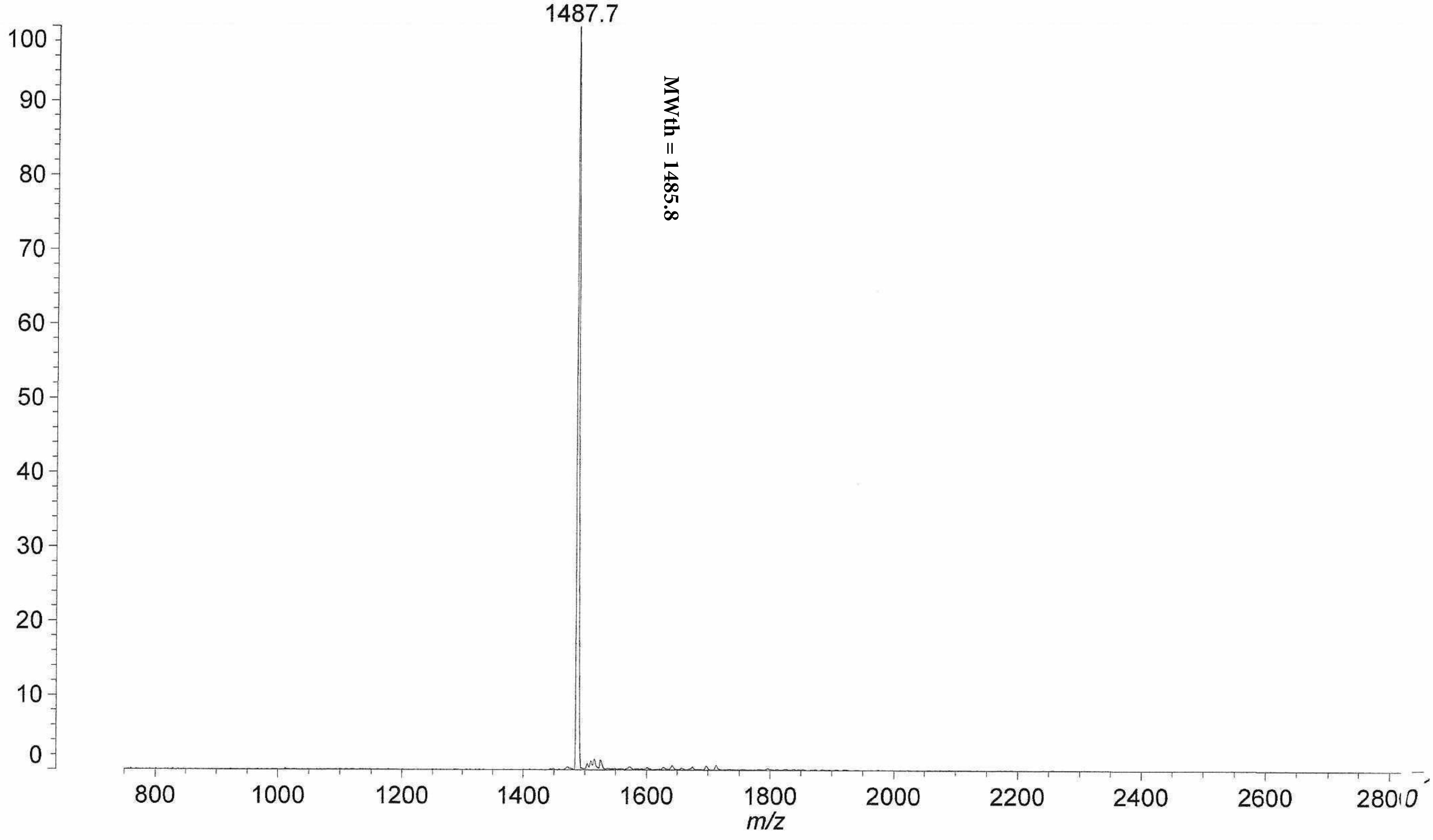

SMAD7 **HPLC-Chromatogramm**

Sample Name : 50297-Fraktion
Sample ID :
Data File Name : 50297- P.lcd
Method File Name : Reprosil C18_250x3mm.lcm
Data Acquired : 14.07.2025 12:59:35
Data Processed : 14.07.2025 13:22:24

gradient: 5-80%B in 30min, Temp55°C
Column: Reprosil ES RP18; 5µm (250mm x 3mm) A79
0,6 ml/min

C:\LabSolutions\Data\PDA 4\50297- P.lcd

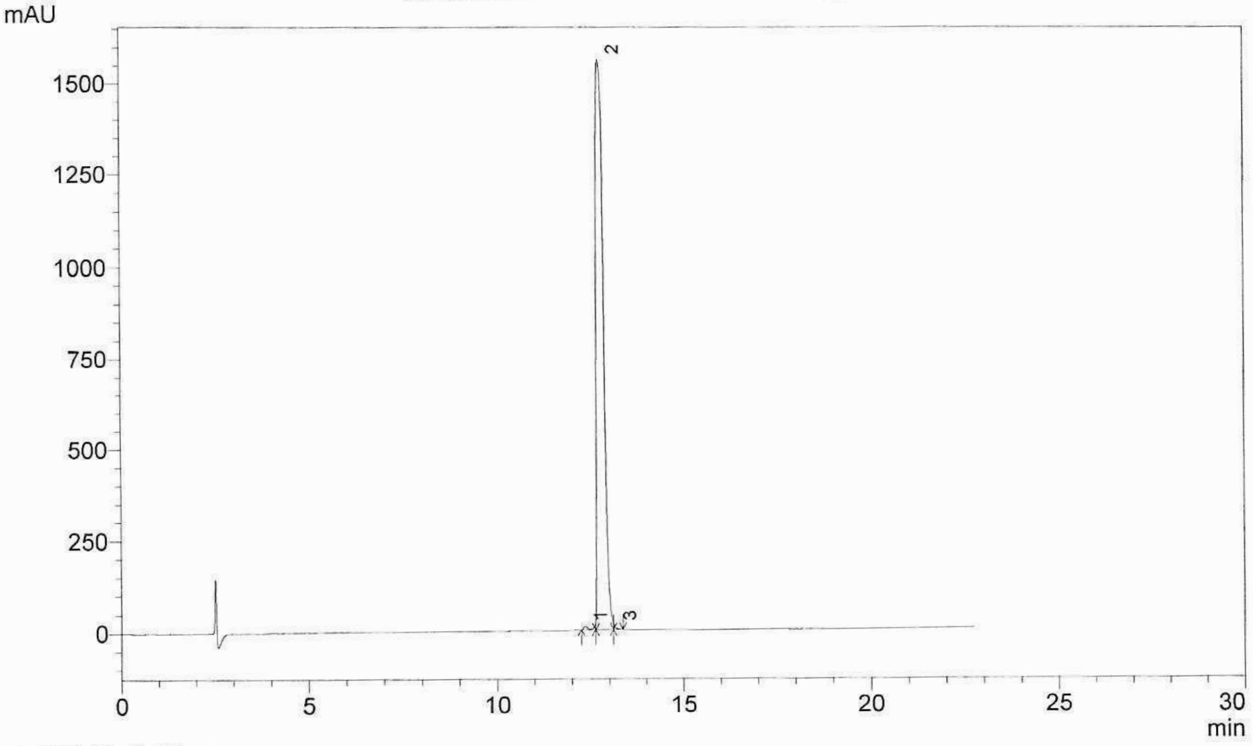

1 PDA Multi 1/220nm 4nm

PeakTable

PDA Ch1 220nm 4nm

Peak#	Ret. Time	Area	Height	Area %
1	12.36	122192	9218	0.6
2	12.76	20962036	1554263	99.2
3	13.15	49741	11079	0.2
Total		21133969		100.0

TH
CHCA 50297-P
Data: <Untitled>.E4[c] 14 Jul 2025 12:39 Cal: MIX CAL Slide 4 10 Jul 2025 15:44
Shimadzu Biotech Axima Assurance 2.9.3.20110624: Mode Linear, Power: 32, Blanked, P.Ext. @ 1443 (bin 57)
%Int. 220 mV[sum= 30121 mV] Profiles 1-137 Smooth Gauss 100 -Baseline 100

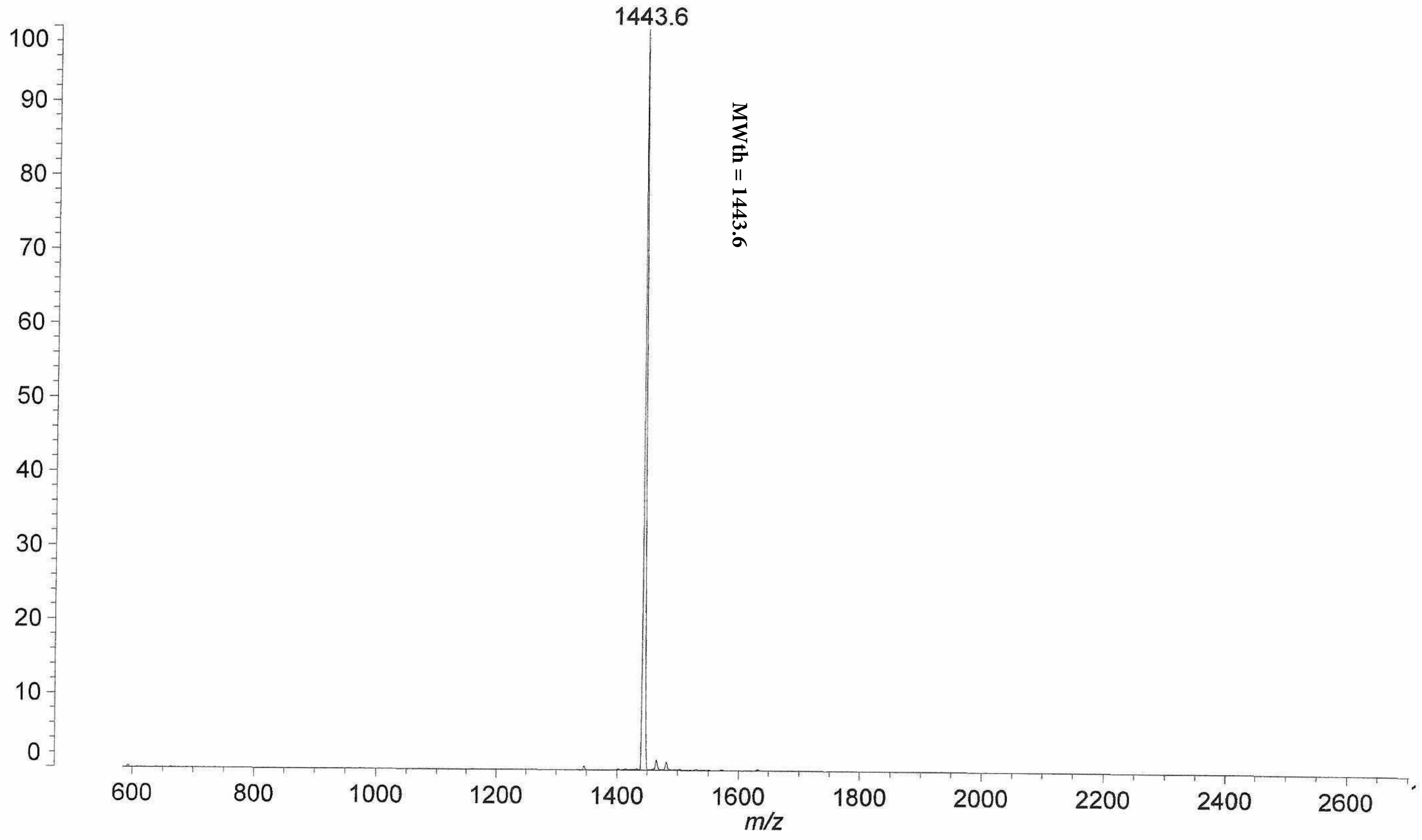

PITCH1_2 HPLC-Chromatogramm

Sample Name : 30681-Fraktion
Sample ID :
Data File Name : 30681-P.lcd
Method File Name : Reprosil C18_250x3mm.lcm
Data Acquired : 05.12.2023 15:46:31
Data Processed : 05.12.2023 16:04:04

gradient: 5-80%B in 30min, 55°C
column: Reprosil Gold RP18; 5µm (250mm x 3mm)
0,6 ml/min

C:\LabSolutions\Data\PDA 4\30681-P.lcd

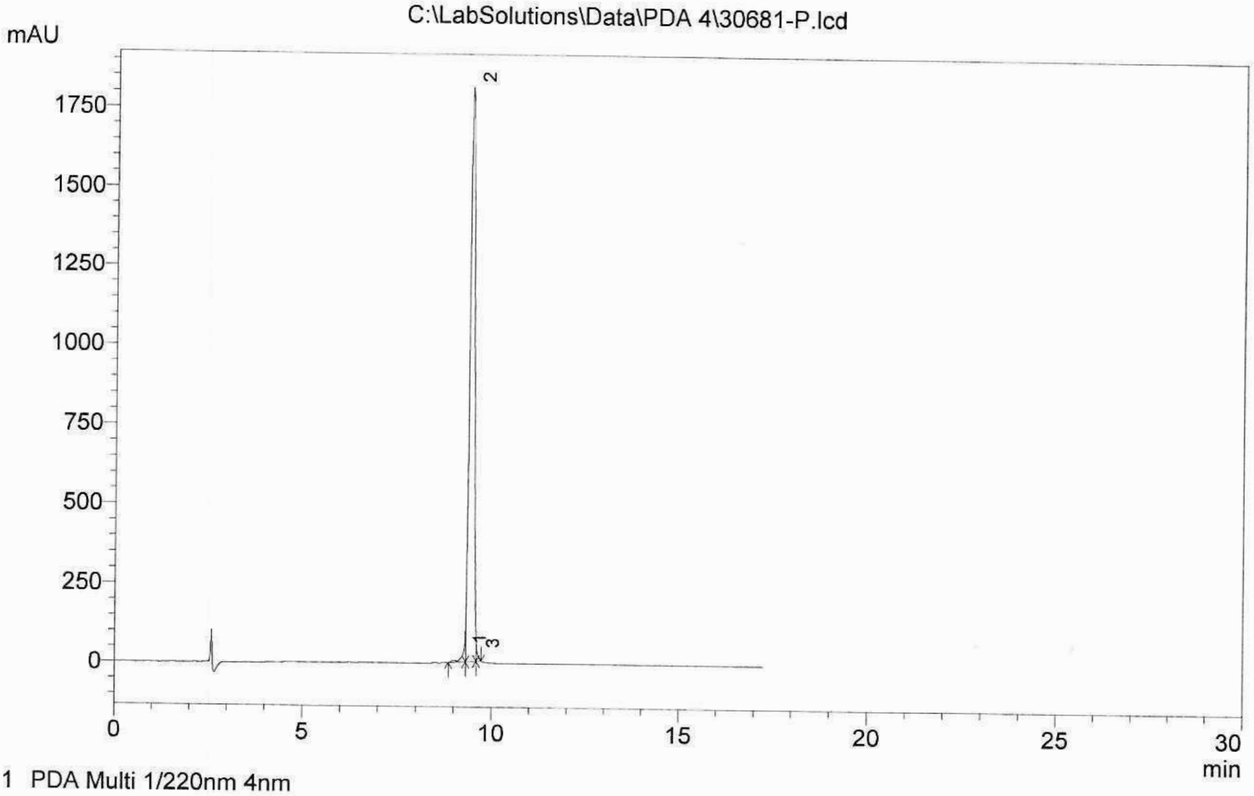

1 PDA Multi 1/220nm 4nm

PeakTable

PDA Ch1 220nm 4nm

Peak#	Ret. Time	Area	Height	Area %
1	9.28	374468	35854	2.1
2	9.41	17287392	1771717	97.3
3	9.63	108299	38063	0.6
Total		17770158		100.0

MK
CHCA- 30681-P
Data: <Untitled>.M20[c] 5 Dec 2023 15:41 Cal: 25 Apr 2016 11:28
Shimadzu Biotech Axima Assurance 2.9.3.20110624: Mode Linear, Power: 35, Blanked, P.Ext. @ 1415 (bin 57)
%Int. 97 mV[sum= 9399 mV] Profiles 1-97 Smooth Gauss 100 -Baseline 100

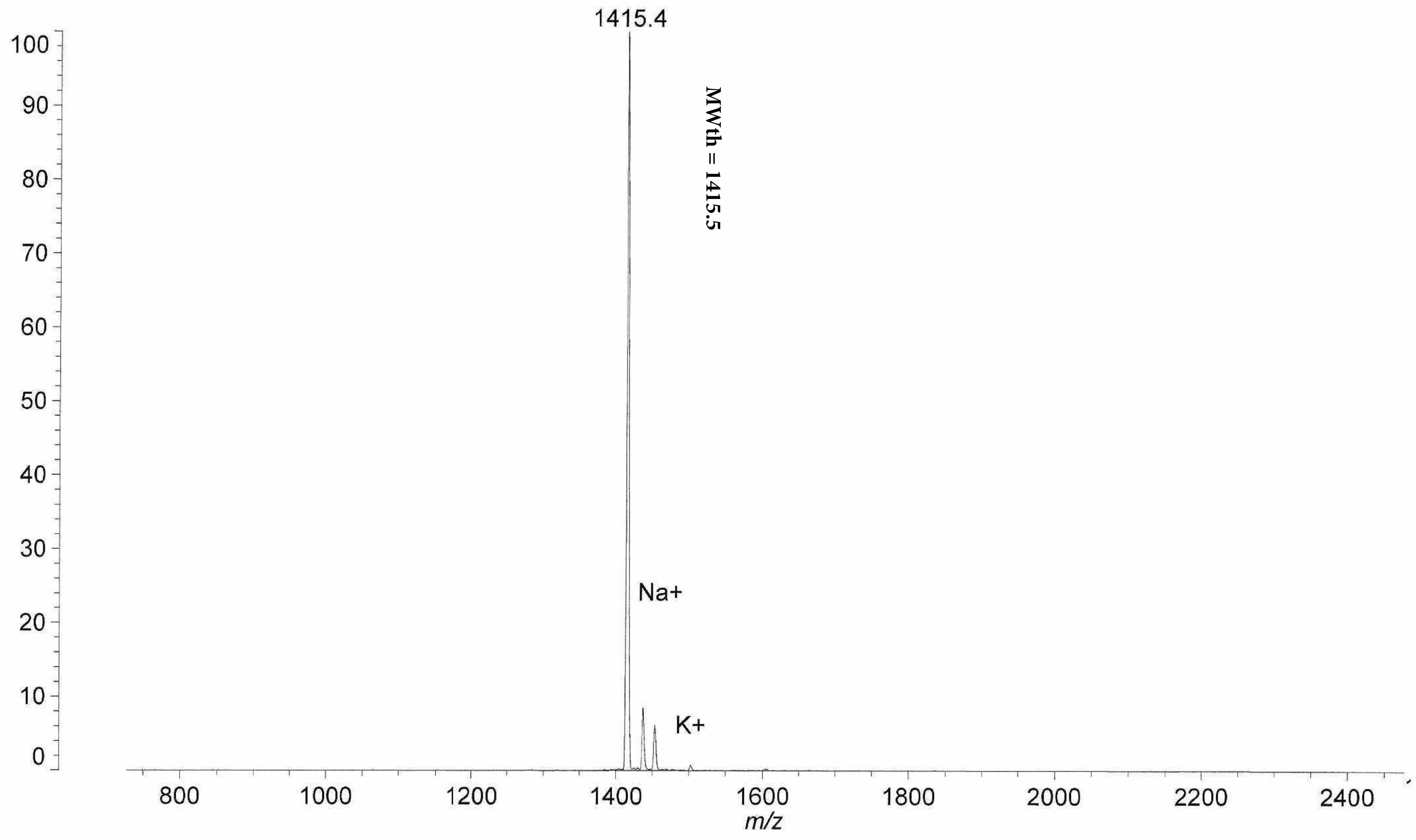

PITCH1_2 HPLC-Chromatogramm

Sample Name	: 30682-Fraktion
Sample ID	:
Data File Name	: 30682-P.lcd
Method File Name	: Reprosil C18_250x3mm.lcm
Data Acquired	: 05.12.2023 17:14:45
Data Processed	: 05.12.2023 17:36:04

gradient: 5-80%B in 30min, 55°C
column: Reprosil Gold RP18; 5µm (250mm x 3mm)
0,6 ml/min

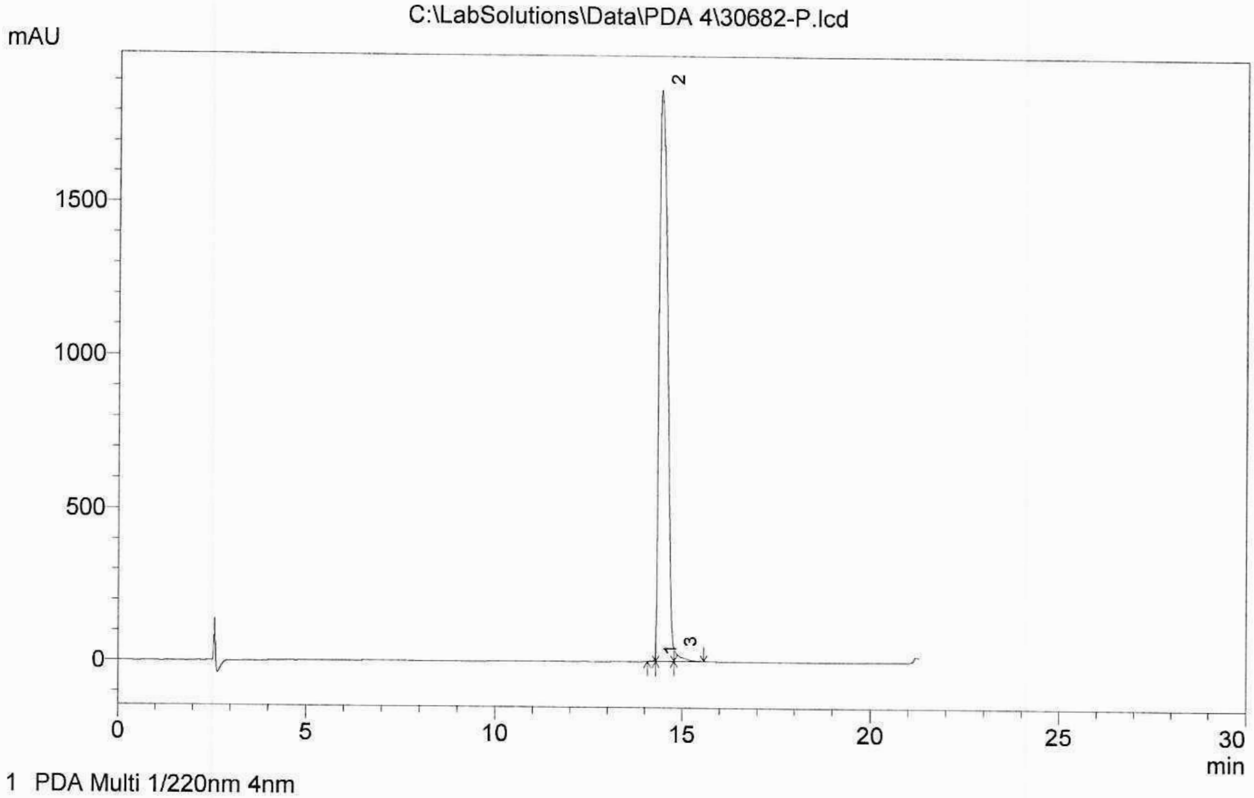

1 PDA Multi 1/220nm 4nm

PeakTable

PDA Ch1 220nm 4nm

Peak#	Ret. Time	Area	Height	Area %
1	14.27	80700	4338	0.3
2	14.41	28681180	1856571	98.3
3	14.82	400802	35150	1.4
Total		29162683		100.0

MK
CHCA- 30682-P
Data: <Untitled>.C21[c] 5 Dec 2023 16:37 Cal: 25 Apr 2016 11:28
Shimadzu Biotech Axima Assurance 2.9.3.20110624: Mode Linear, Power: 33, Blanked, P.Ext. @ 1564 (bin 60)
%Int. 19 mV[sum= 701 mV] Profiles 54-90 Smooth Gauss 100 -Baseline 100

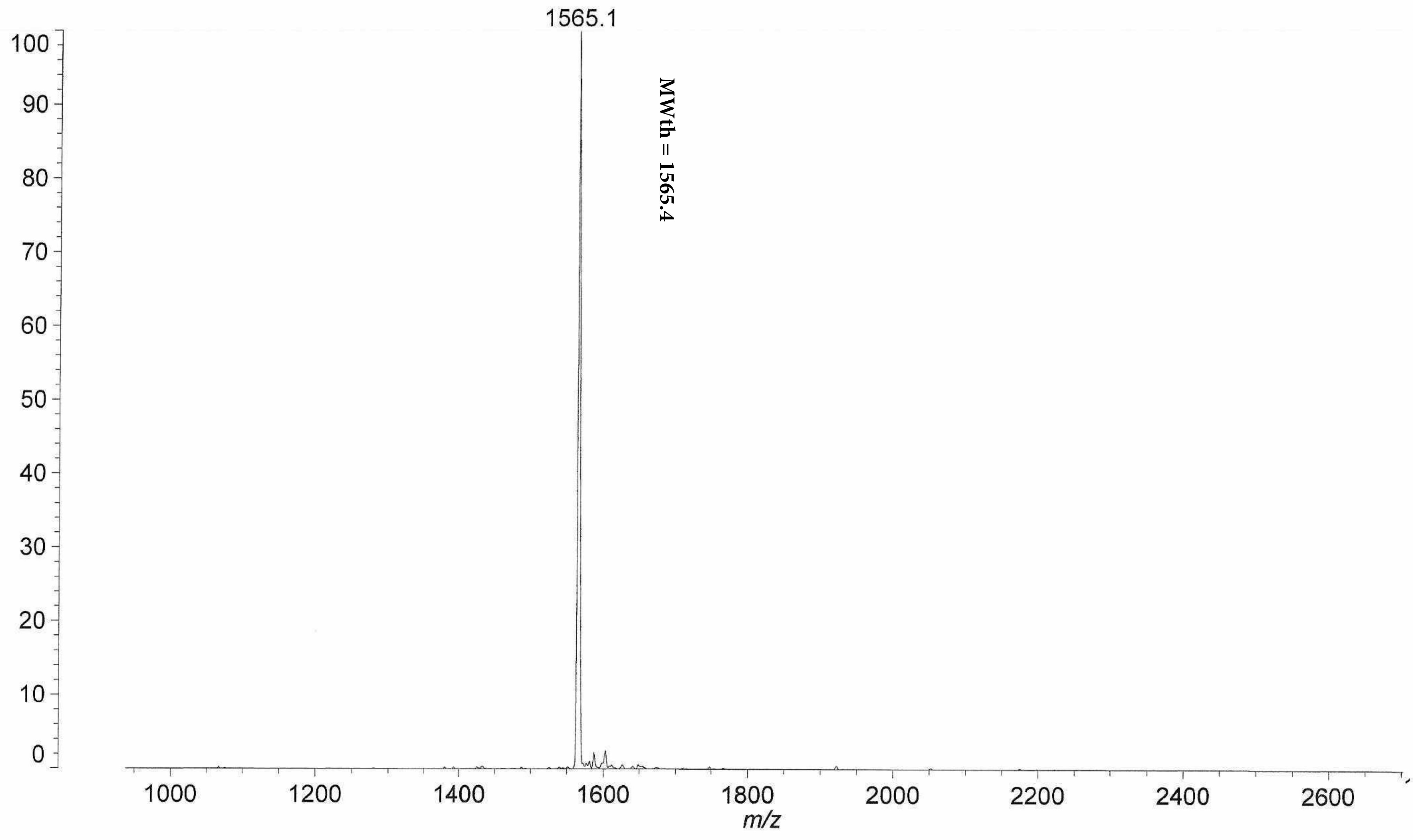

Peptide 1 **HPLC-DATA-SHEET**

File: C:\CLASS-VP\PUBLIC\DAD 3\40199-P001
Method: C:\CLASS-VP\Methods\ana-reprosil.met
Description: gradient: 5-80% B in 30min, Temp 55°C
Aquired: 25.04.2024 14:56:38
Printed: 25.04.2024 15:37:57
Column: Reprosil Gold RP18 250 x 3 mm,
Eluent A: 0,05% TFA/Wasser
Eluent B: 0,05% TFA/80% AcN/Wasser
Flow Rate: 0,6 ml/min

Chromatogramm

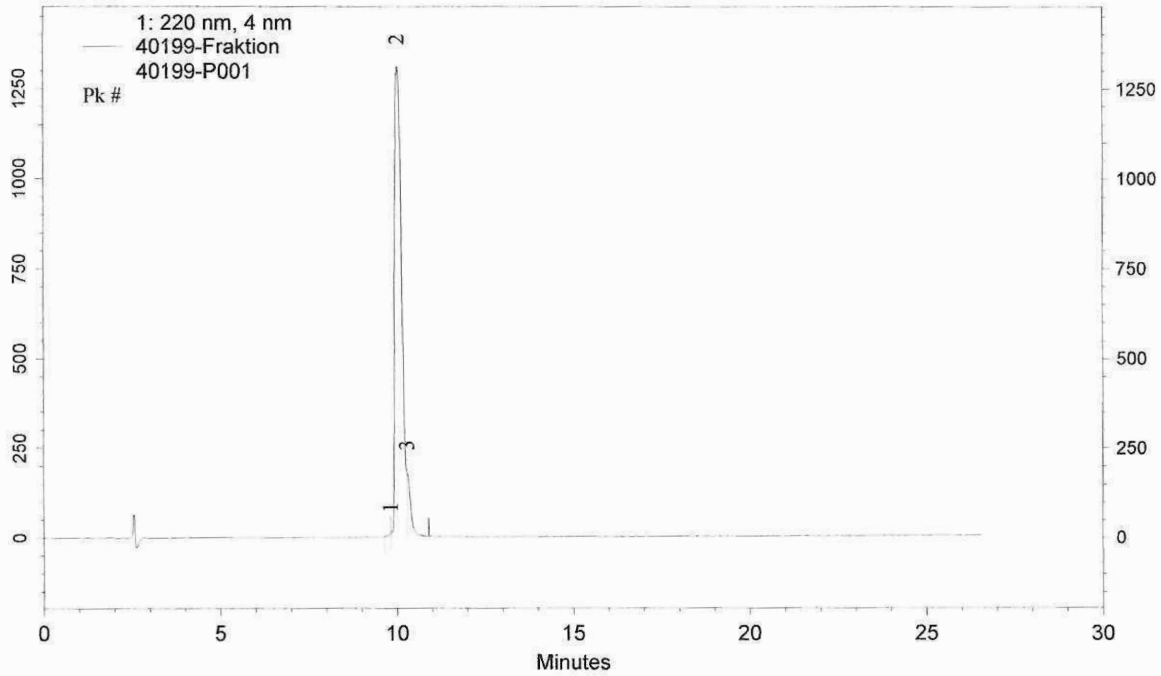

1: 220 nm, 4 nm

Pk #	Retention Time	Area	Area Percent	Name
1	9.8	35989	0.2	
2	10.0	19046502	93.6	
3	10.3	1265252	6.2	

Totals				
		20347743	100.0	

TH
CHCA- 40199-P
Data: <Untitled>.B12[c] 25 Apr 2024 14:19 Cal: 25 Apr 2016 11:28
Shimadzu Biotech Axima Assurance 2.9.3.20110624: Mode Linear, Power: 31, Blanked, P.Ext. @ 1529 (bin 59)
%Int. 83 mV[sum= 4456 mV] Profiles 1-54 Smooth Gauss 100 -Baseline 100

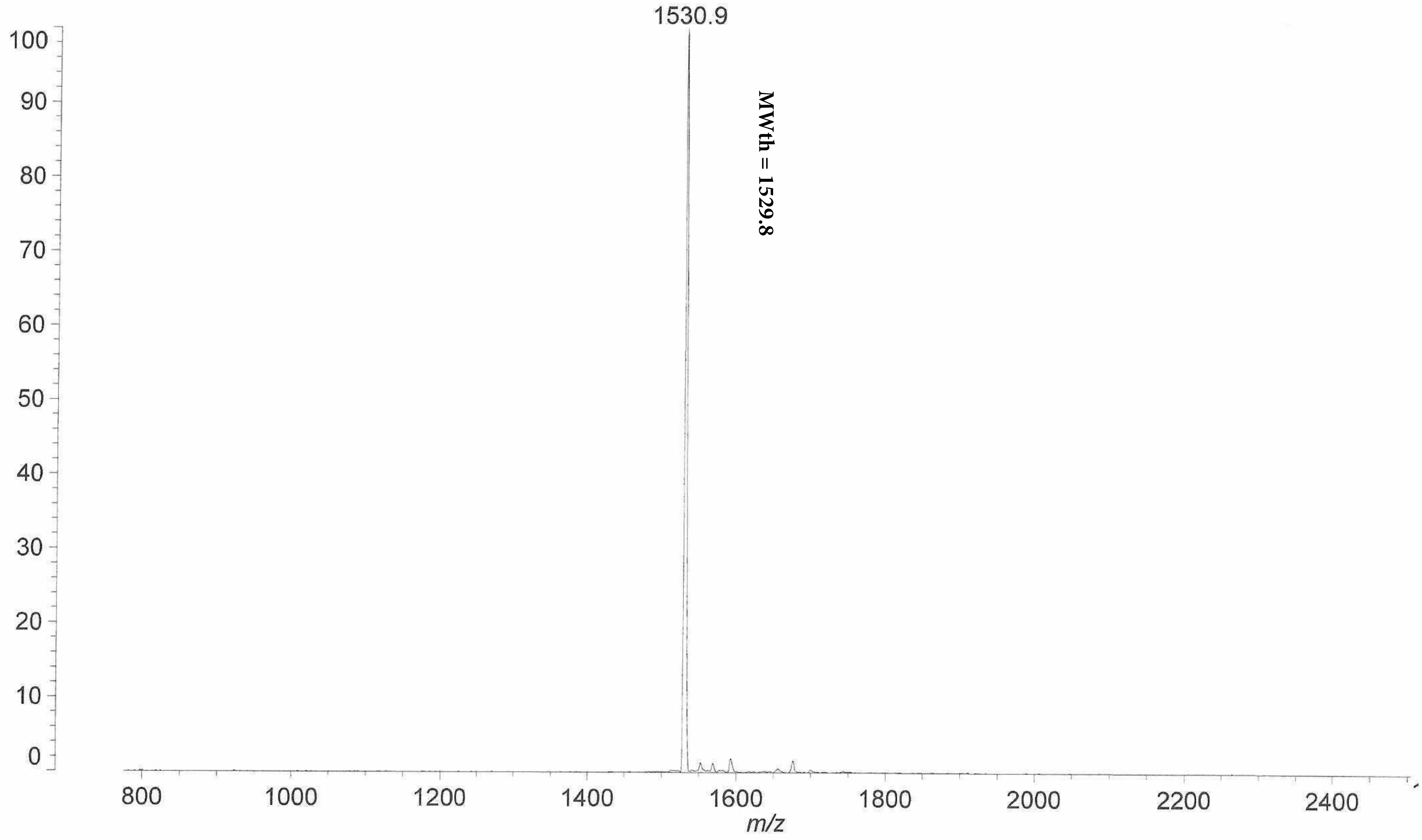

Peptide 2 **HPLC-DATA-SHEET**

File: C:\CLASS-VP\PUBLIC\DAD 3\40200-P001
Method: C:\CLASS-VP\Methods\ana-reprosil.met
Description: gradient: 5-80% B in 30min, Temp 55°C
Aquired: 25.04.2024 16:25:44
Printed: 25.04.2024 16:49:26
Column: Reprosil Gold RP18 250 x 3 mm,
Eluent A: 0,05% TFA/Wasser
Eluent B: 0,05% TFA/80% AcN/Wasser
Flow Rate: 0,6 ml/min

Chromatogramm

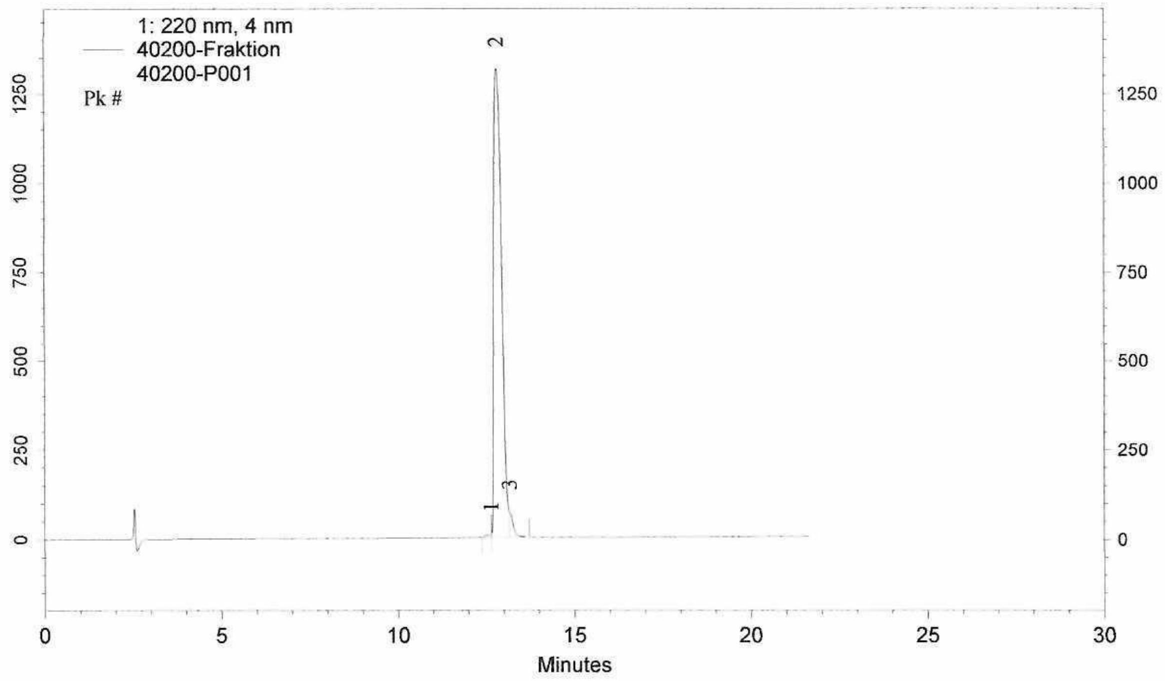

1: 220 nm, 4 nm

Pk #	Retention Time	Area	Area Percent	Name
1	12.6	79226	0.4	
2	12.8	20845203	97.2	
3	13.2	513595	2.4	

Totals				
		21438024	100.0	

TH
CHCA- 40200-P
Data: <Untitled>.L13[c] 29 Apr 2024 13:28 Cal: MIX CAL Slide 4 9 Apr 2024 12:56
Shimadzu Biotech Axima Assurance 2.9.3.20110624: Mode Linear, Power: 48, Blanked, P.Ext. @ 1415 (bin 57)
%Int. 7.4 mV[sum= 1394 mV] Profiles 1-189 Smooth Gauss 100 -Baseline 100

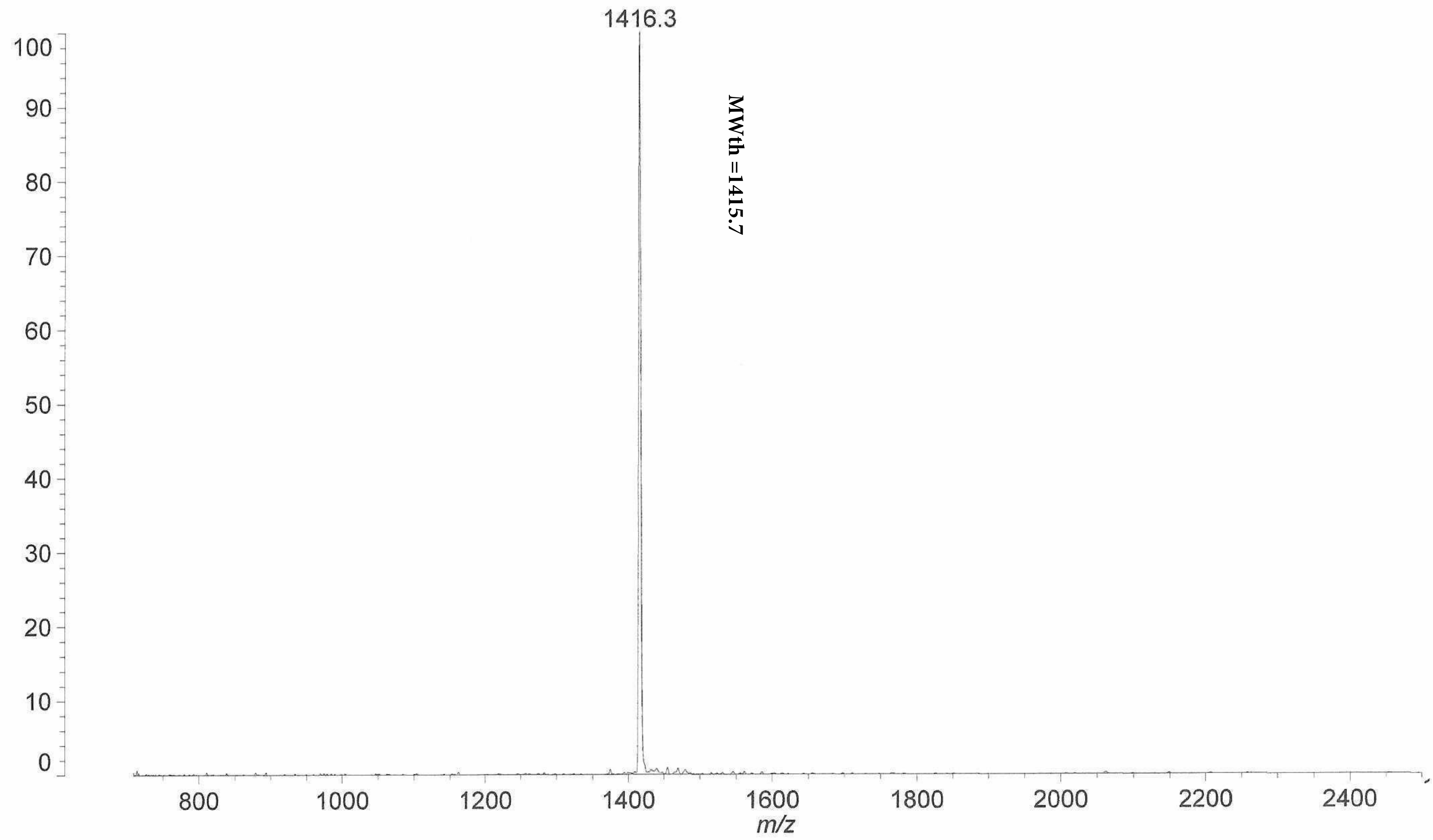

Peptide 3 **HPLC-DATA-SHEET**

File:	C:\CLASS-VP\PUBLIC\DAD 3\40202-P
Method:	C:\CLASS-VP\Methods\ana-reprosil.met
Description:	gradient: 5-80% B in 30min, Temp 55°C
Aquired:	26.04.2024 11:04:20
Printed:	26.04.2024 11:28:05
Column:	Reprosil Gold RP18 250 x 3 mm,
Eluent A:	0,05% TFA/Wasser
Eluent B:	0,05% TFA/80% AcN/Wasser
Flow Rate:	0,6 ml/min

Chromatogramm

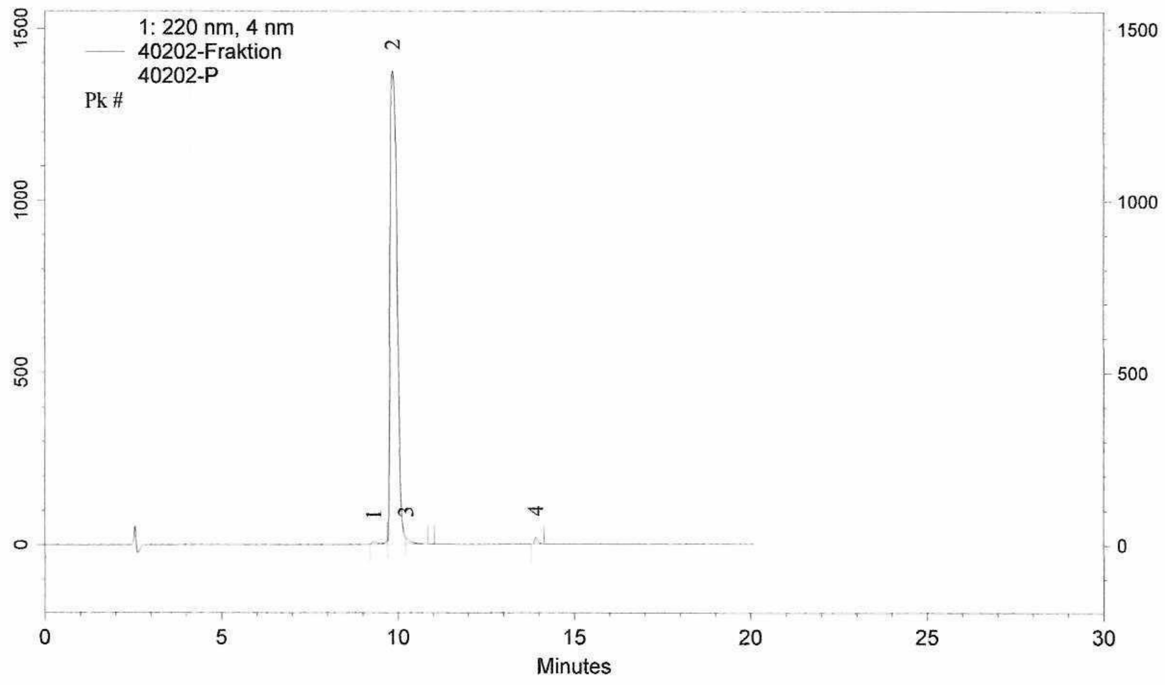

1: 220 nm, 4 nm

Pk #	Retention Time	Area	Area Percent	Name
1	9.3	105078	0.5	
2	9.9	18806781	98.1	
3	10.2	146978	0.8	
4	13.9	115183	0.6	

Totals				
		19174020	100.0	

TH
CHCA- 40202-P
Data: <Untitled>.D10[c] 26 Apr 2024 10:21 Cal: 25 Apr 2016 11:28
Shimadzu Biotech Axima Assurance 2.9.3.20110624: Mode Linear, Power: 30, Blanked, P.Ext. @ 1486 (bin 58)
%Int. 110 mV[sum= 4409 mV] Profiles 1-40 Smooth Gauss 100 -Baseline 100

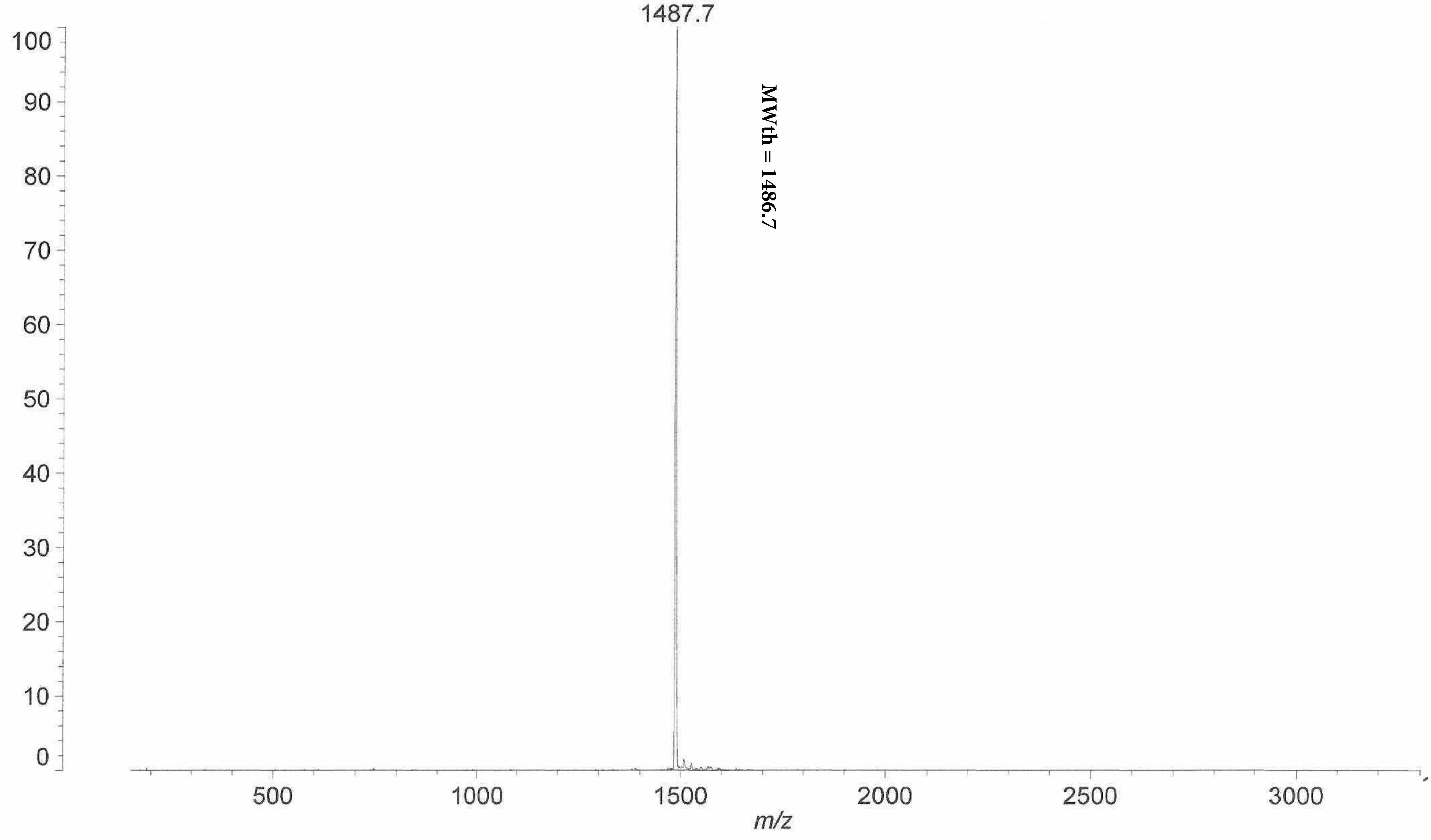

Peptide 4 # HPLC-DATA-SHEET

File:	C:\CLASS-VP\PUBLIC\DAD 3\40203-P
Method:	C:\CLASS-VP\Methods\ana-reprosil.met
Description:	gradient: 5-80% B in 30min, Temp 55°C
Aquired:	26.04.2024 11:55:47
Printed:	26.04.2024 12:23:08
Column:	Reprosil Gold RP18 250 x 3 mm,
Eluent A:	0,05% TFA/Wasser
Eluent B:	0,05% TFA/80% AcN/Wasser
Flow Rate:	0,6 ml/min

Chromatogramm

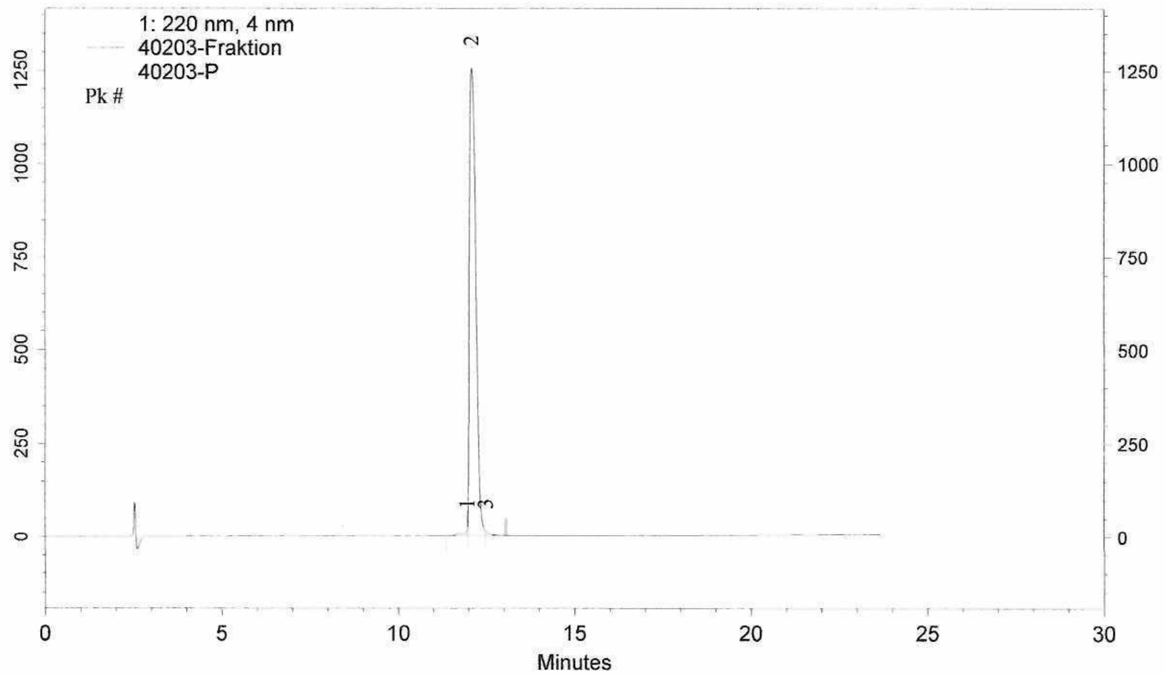

1: 220 nm, 4 nm

Pk #	Retention Time	Area	Area Percent	Name
1	12.0	152524	0.9	
2	12.1	15983183	98.4	
3	12.5	103564	0.6	

Totals		16239271	100.0	

TH
CHCA- 40203-P
Data: <Untitled>.E13[c] 26 Apr 2024 11:40 Cal: 25 Apr 2016 11:28
Shimadzu Biotech Axima Assurance 2.9.3.20110624: Mode Linear, Power: 27, Blanked, P.Ext. @ 1470 (bin 58)
%Int. 129 mV[sum= 8101 mV] Profiles 1-63 Smooth Gauss 100 -Baseline 100

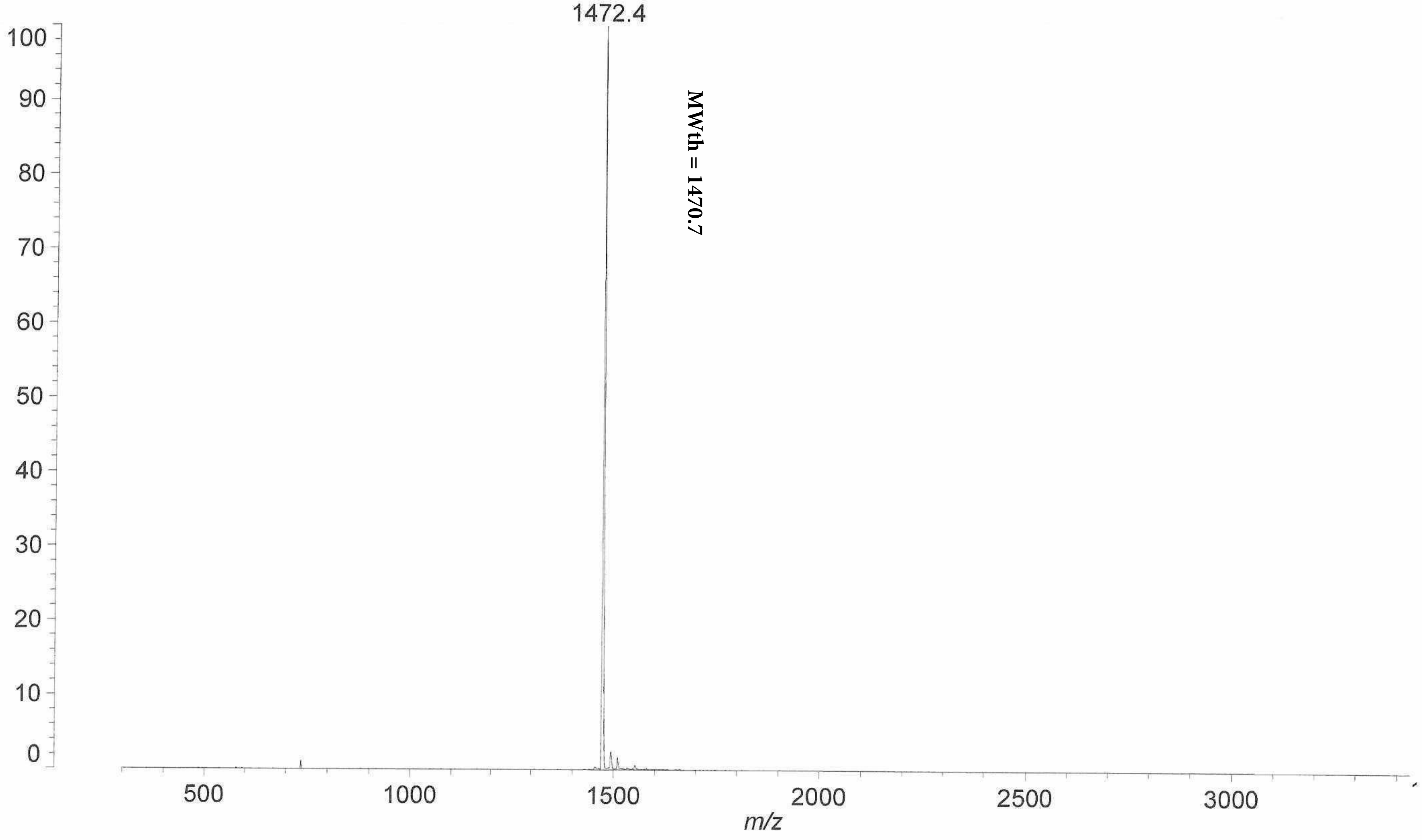

Peptide 5 # HPLC-DATA-SHEET

File:	C:\CLASS-VP\PUBLIC\DAD 3\40204-P
Method:	C:\CLASS-VP\Methods\ana-reprosil.met
Description:	gradient: 5-80% B in 30min, Temp 55°C
Aquired:	26.04.2024 13:23:36
Printed:	26.04.2024 14:03:51
Column:	Reprosil Gold RP18 250 x 3 mm,
Eluent A:	0,05% TFA/Wasser
Eluent B:	0,05% TFA/80% AcN/Wasser
Flow Rate:	0,6 ml/min

Chromatogramm

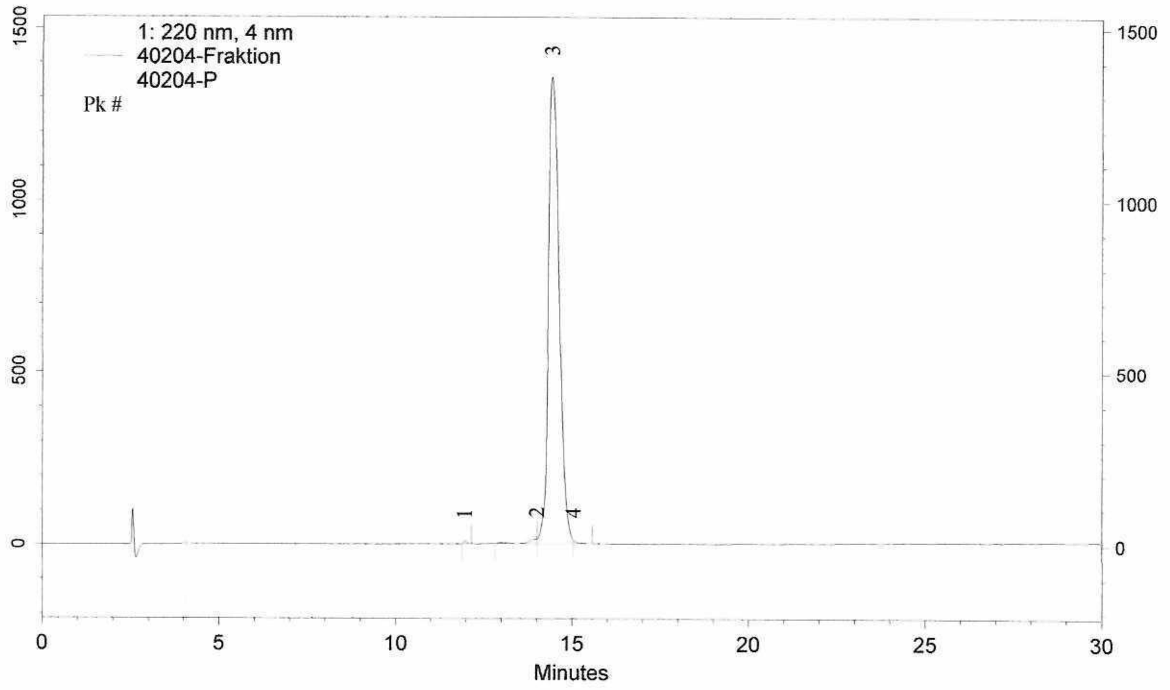

1: 220 nm, 4 nm

Pk #	Retention Time	Area	Area Percent	Name
1	12.0	48509	0.2	
2	14.0	252419	0.9	
3	14.4	29093757	98.7	
4	15.0	68237	0.2	

Totals				
		29462922	100.0	

TH
CHCA- 40204-P
Data: <Untitled>.D11[c] 26 Apr 2024 12:54 Cal: 25 Apr 2016 11:28
Shimadzu Biotech Axima Assurance 2.9.3.20110624: Mode Linear, Power: 27, Blanked, P.Ext. @ 1459 (bin 58)
%Int. 36 mV[sum= 2514 mV] Profiles 1-69 Smooth Gauss 100 -Baseline 100

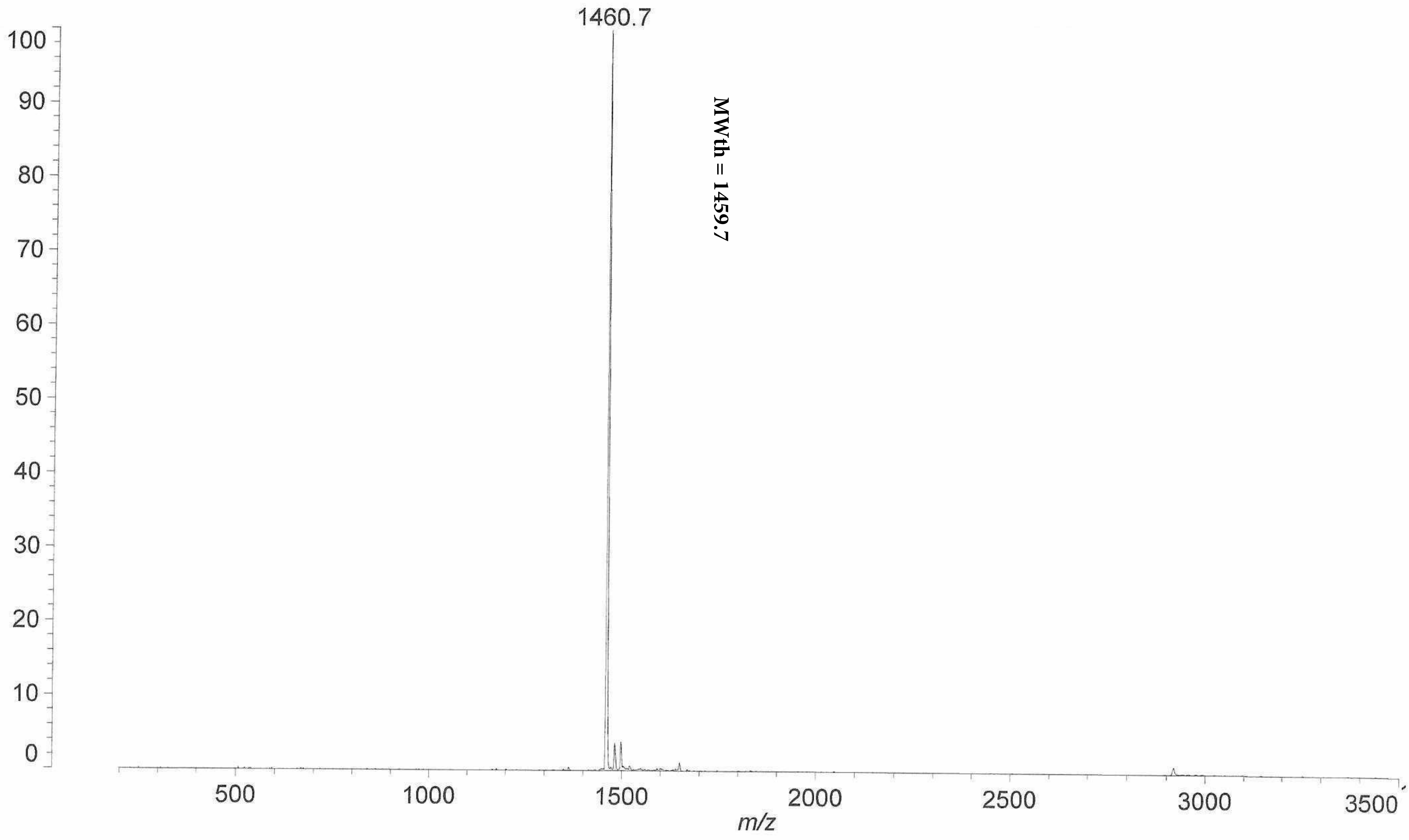

Peptide 6 **HPLC-DATA-SHEET**

File: C:\CLASS-VP\PUBLIC\DAD 3\40205-P
Method: C:\CLASS-VP\Methods\ana-reprosil.met
Description: gradient: 5-80% B in 30min, Temp 55°C
Aquired: 26.04.2024 15:08:49
Printed: 26.04.2024 15:37:08
Column: Reprosil Gold RP18 250 x 3 mm,
Eluent A: 0,05% TFA/Wasser
Eluent B: 0,05% TFA/80% AcN/Wasser
Flow Rate: 0,6 ml/min

Chromatogramm

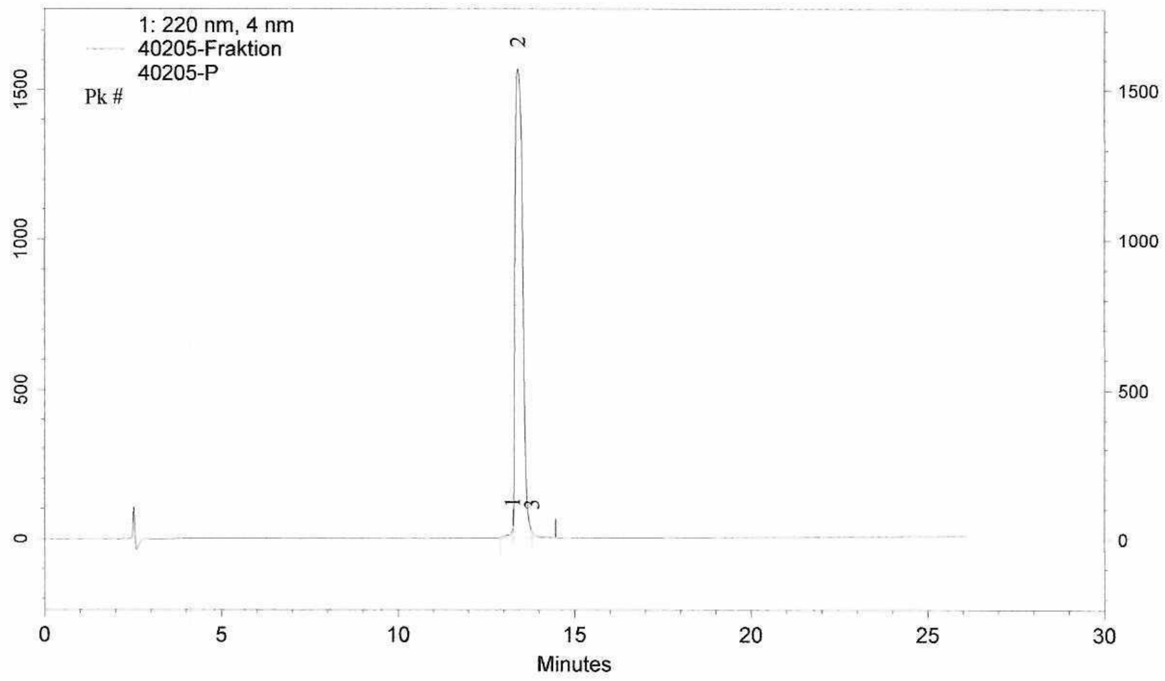

1: 220 nm, 4 nm

Pk #	Retention Time	Area	Area Percent	Name
1	13.2	202235	0.8	
2	13.4	23419310	98.4	
3	13.8	171120	0.7	

Totals				
		23792665	100.0	

TH
CHCA- 40205-P
Data: <Untitled>.O12[c] 26 Apr 2024 14:59 Cal: 25 Apr 2016 11:28
Shimadzu Biotech Axima Assurance 2.9.3.20110624: Mode Linear, Power: 29, Blanked, P.Ext. @ 1453 (bin 57)
%Int. 24 mV[sum= 1767 mV] Profiles 1-74 Smooth Gauss 100 -Baseline 100

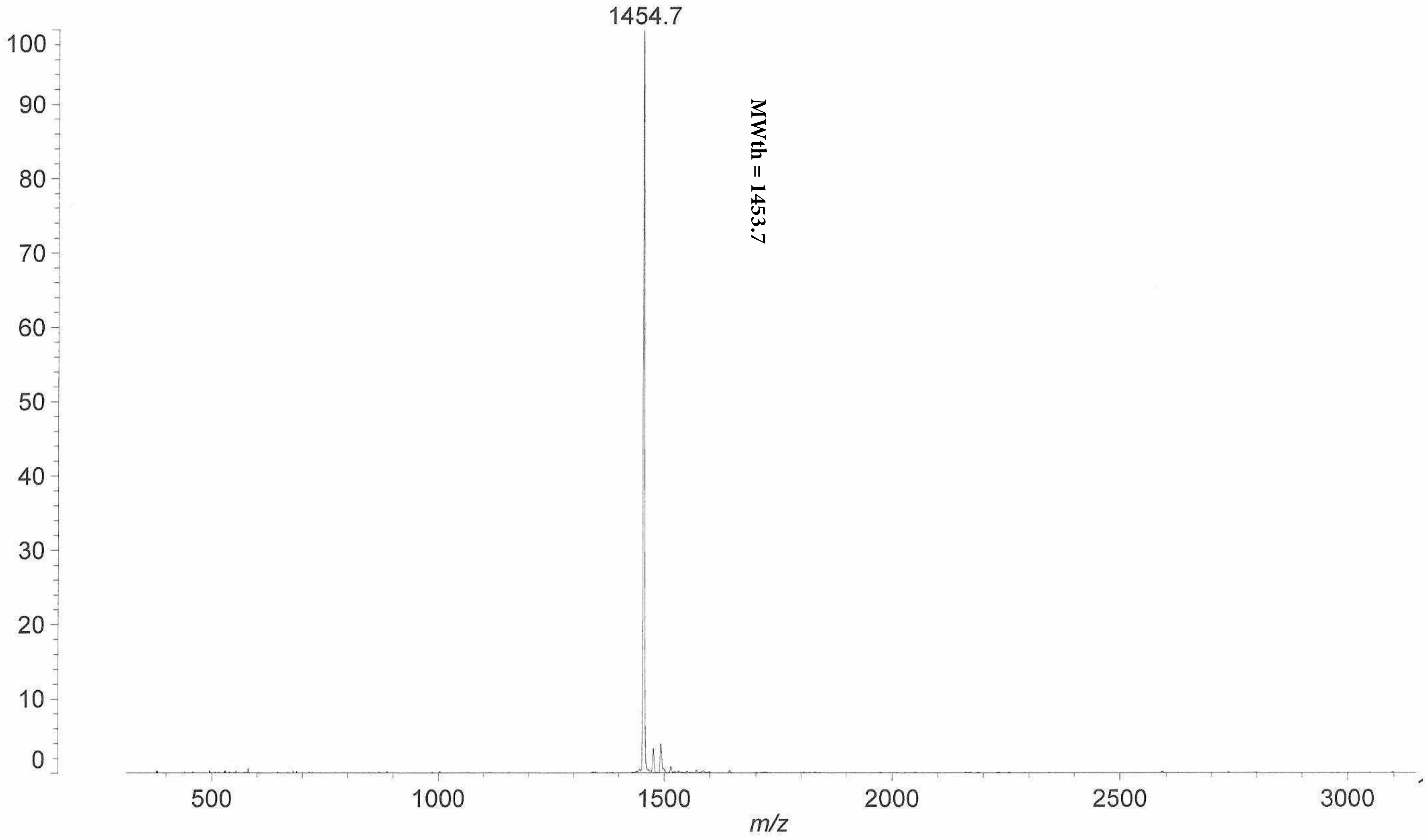

Peptide 7 HPLC-Chromatogramm

Sample Name	: 50662-Fraktion
Sample ID	:
Data File Name	: 50662- P.lcd
Method File Name	: Reprosil C18_250x3mm.lcm
Data Acquired	: 16.12.2025 10:47:21
Data Processed	: 16.12.2025 11:07:16

gradient: 5-80%B in 30min, Temp55°C
Column: Reprosil ES RP18; 5µm (250mm x 3mm) A79
0,6 ml/min

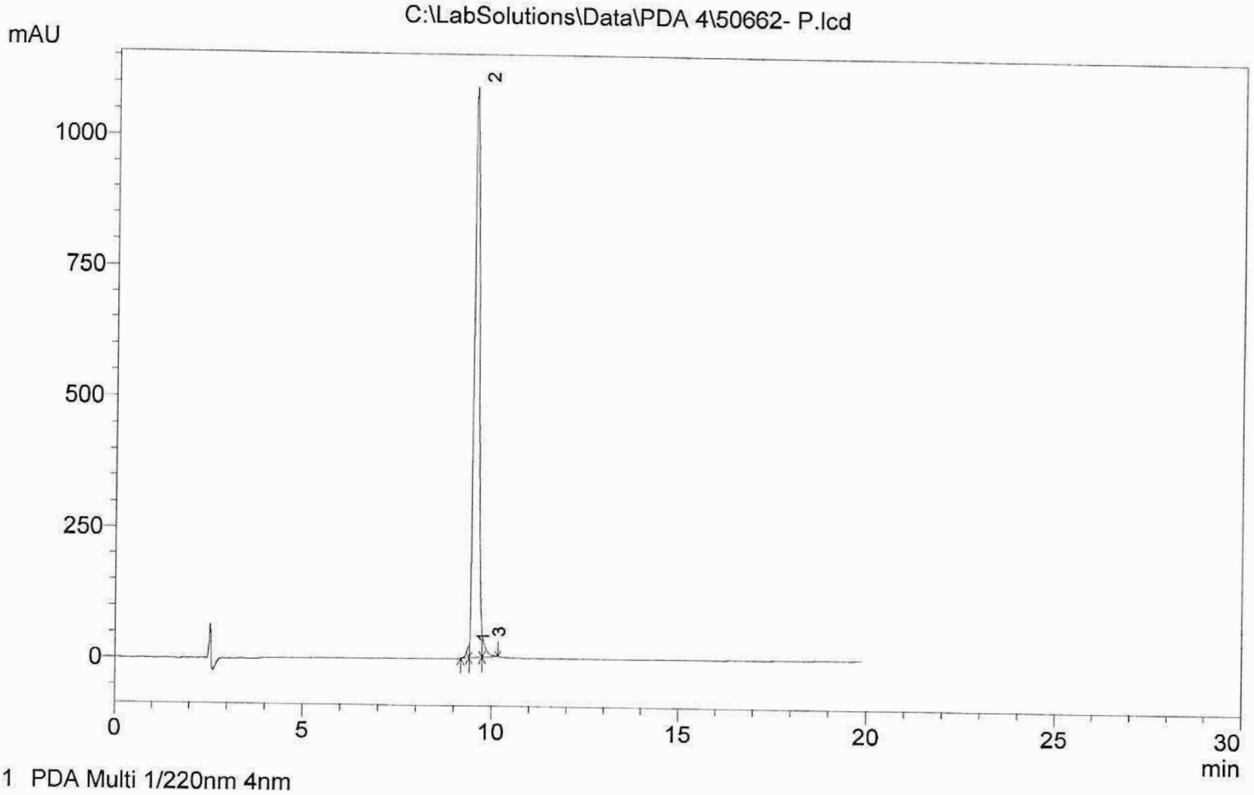

1 PDA Multi 1/220nm 4nm

PeakTable

PDA Ch1 220nm 4nm

Peak#	Ret. Time	Area	Height	Area %
1	9.38	115873	18746	1.1
2	9.52	10498991	1081377	96.9
3	9.79	216815	31993	2.0
Total		10831679		100.0

TH
CHCA 50662-P
Data: <Untitled>.J17[c] 16 Dec 2025 9:24 Cal: 16 Dec 2025 9:25
Shimadzu Biotech Axima Assurance 2.9.3.20110624: Mode Linear, Power: 32, Blanked, P.Ext. @ 1491 (bin 58)
%Int. 83 mV[sum= 11719 mV] Profiles 1-142 Smooth Gauss 100 -Baseline 100

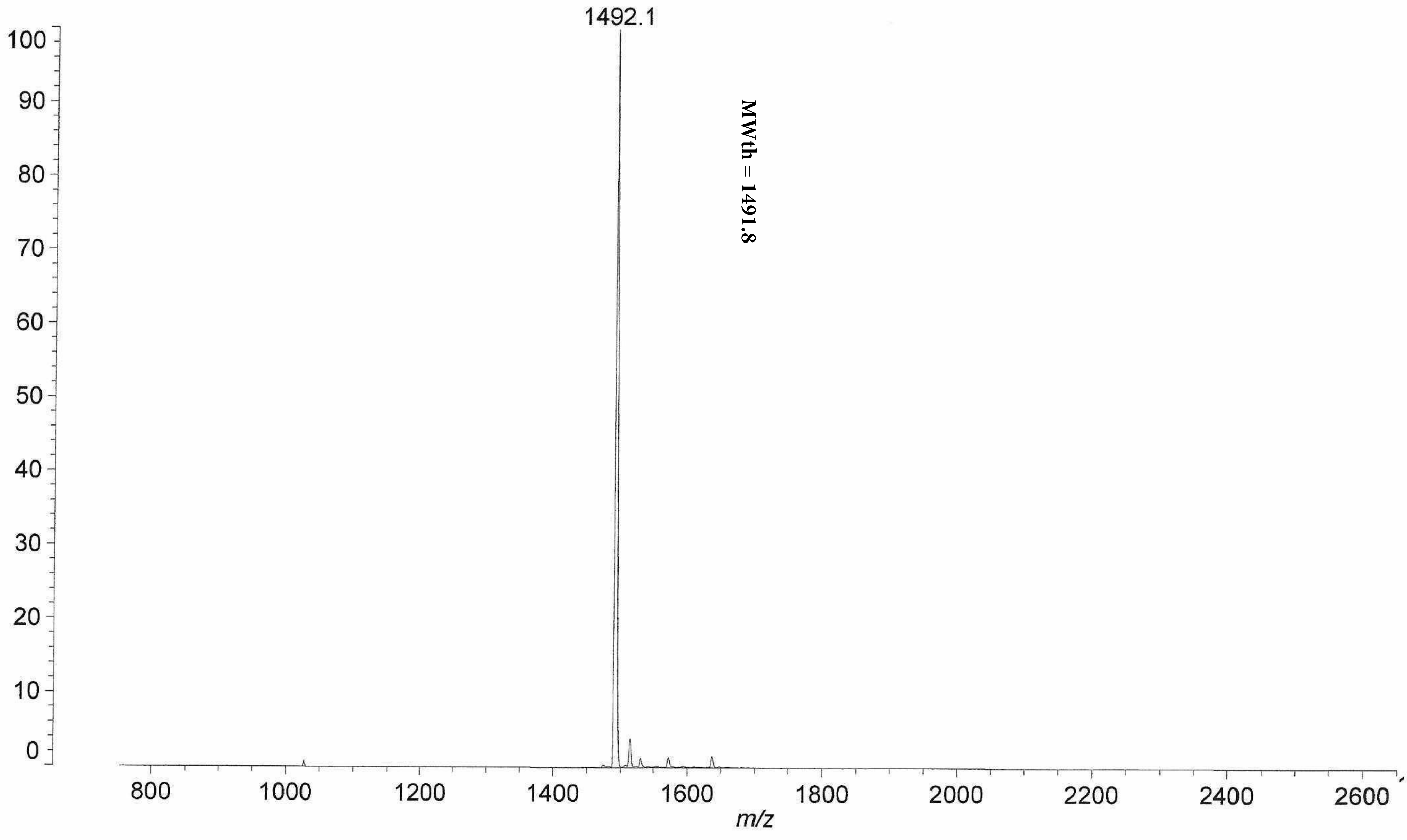

Supplementary Figure S12

Peptides	Name	n=1	n=2	n=3	n=4	n=5	Average (nM)	Standard Deviation (nM)	95% Confidence Interval (± nM)		
		YAP-Avi - Kd (µM)								KdTAZ/KdYAP	Propagated errors
Ac-LESPPPPYSRYP-NH2	**SMAD7**	5.65E-05	6.39E-05	7.13E-05	6.96E-05	6.66E-05	66	6	7	4.4	0.7
Ac-RRCPPPPYSRYP-NH2	**1**	1.11E-05	1.12E-05	1.13E-05			11.2	0.1	0.3	3.0	0.5
Ac-LESPPPPYPKHL-NH2	**2**	3.97E-05	4.12E-05	3.57E-05			39	3	7	2.2	0.2
Ac-RESPPPPYSRYP-NH2	**3**	3.43E-05	4.08E-05	3.68E-05			37	3	8	3.3	0.5
Ac-LRSPPPPYSRYP-NH2	**4**	2.16E-05	2.99E-05	2.43E-05	2.72E-05		26	4	6	3.5	0.7
Ac-LECPPPPYSRYP-NH2	**5**	4.88E-05	5.97E-05	4.88E-05			52	6	16	3.5	0.5
Ac-LESPPPPYPRYP-NH2	**6**	6.24E-05	6.83E-05	6.64E-05	6.71E-05		66	3	4.1	2.3	0.2
Ac-RRCPPPPYSKHL-NH2	**7**	1.34E-05	1.33E-05	1.37E-05			13.5	0.2	0.5	2.4	0.0
Ac-RRCPPPPYPKHL-NH2	**LATS2**	4.54E-06	5.63E-06	6.32E-06	5.32E-06		5	1	1	2.1	0.3
		TAZ-Avi - Kd (µM)									
Ac-LESPPPPYSRYP-NH2	**SMAD7**	2.94E-04	2.45E-04	3.19E-04			286	38	94		
Ac-RRCPPPPYSRYP-NH2	**1**	2.62E-05	3.69E-05	3.65E-05	3.68E-05		34	5	8		
Ac-LESPPPPYPKHL-NH2	**2**	8.84E-05	7.98E-05	8.60E-05	8.48E-05		85	4	6		
Ac-RESPPPPYSRYP-NH2	**3**	1.17E-04	1.16E-04	1.40E-04			124	13	33		
Ac-LRSPPPPYSRYP-NH2	**4**	1.03E-04	8.95E-05	7.79E-05			90	13	31		
Ac-LECPPPPYSRYP-NH2	**5**	2.02E-04	1.63E-04	1.94E-04	1.77E-04		184	17	27		
Ac-LESPPPPYPRYP-NH2	**6**	1.54E-04	1.70E-04	1.40E-04	1.50E-04		154	12	20		
Ac-RRCPPPPYSKHL-NH2	**7**	3.18E-05	3.19E-05	3.26E-05			32.1	0.4	1.1		
Ac-RRCPPPPYPKHL-NH2	**LATS2**	1.11E-05	1.10E-05	1.21E-05	1.13E-05		11	1	1		
